# Generating, visualizing and tuning π–π interactions in metal-induced supramolecular assembly

**DOI:** 10.1039/d6ce00611f

**Published:** 2026-09-04

**Authors:** Giau Le-Hoang, Laure Guénée, Claude Piguet

**Affiliations:** a Department of Inorganic and Analytical Chemistry, University of Geneva 30 Quai E. Ansermet CH-1211 Geneva 4 Switzerland Hoang.Le@unige.ch Claude.Piguet@unige.ch; b Laboratory of Crystallography, University of Geneva 24 Quai E. Ansermet CH-1211 Geneva 4 Switzerland

## Abstract

This work emphasizes the role of metal complexation for controlling and tuning static/dynamic π-stacked organizations responsible for the programmed light emission of the bichromophoric pyrene–terimine ligand **L2** upon photoexcitation. Its binding to the closed-shell trivalent yttrium guest [Y(hfac)_3_] (hfac = 1,1,1,5,5,5-hexafluoro-pentane-2,4-dione) provides a diamagnetic heteroleptic [**L2**Y(hfac)_3_] adduct for which the static intramolecular pyrene–pyrene excimer emission operating in the free ligand **L2** is transformed into a dynamic intramolecular pyrene–pyrene excimer emission in the resulting complex. The transition from static-to-dynamic intramolecular excimers results in an enhanced sensitivity toward interactions with electron-poor nitrobenzene guests, which is supported by a considerable Stern–Volmer quenching constant measured for [**L2**Y(hfac)_3_]. This demonstrates that the metal-directed organization of the polyaromatic ligand in [**L2**Y(hfac)_3_] can boost the excimeric chemical sensing of target aromatic analytes.

## Abstract 2

The *trans*–*trans* to *cis*–*cis* conformational change upon metal binding disrupts the intramolecular π–π interaction between two stacked pyrene units, leading to the static-to-dynamic excimer switching in the resulting complex.

## Graphical abstract

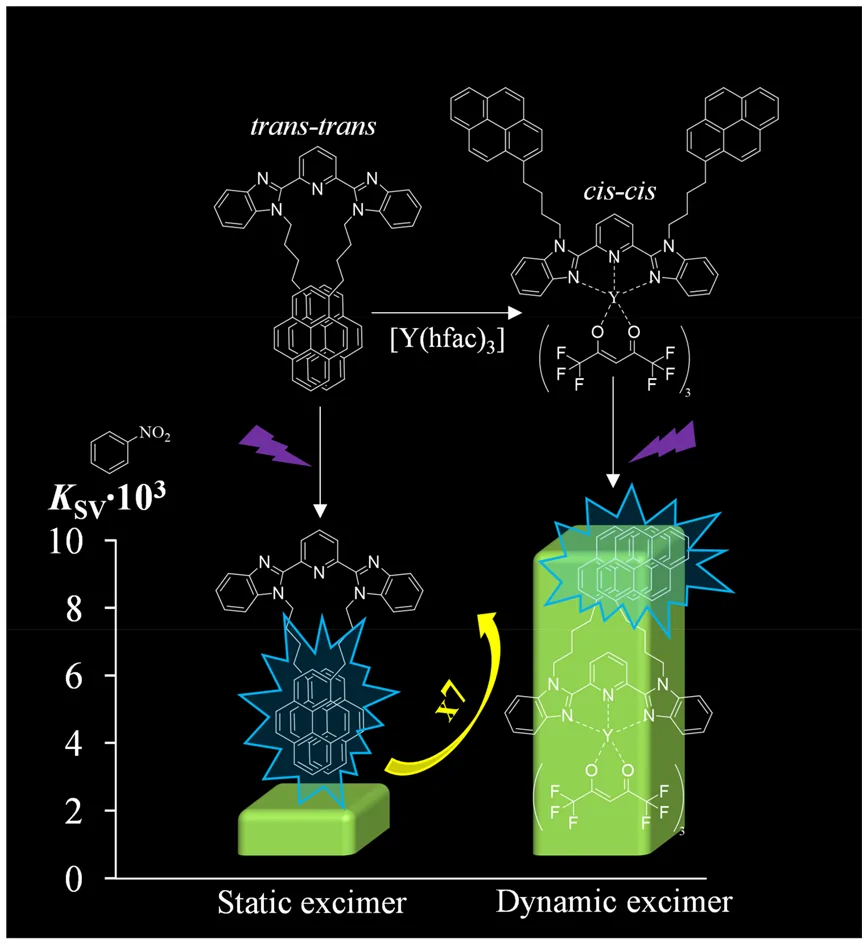

## 1. Introduction

Non-covalent π–π interactions have attracted intense research interest for (i) building innovative supramolecular organization, (ii) tuning molecular recognition, (iii) adjusting photophysical/catalytic properties of conjugated molecules/materials and (iv) developing chemical sensors for external analytes.^1–3^ In order to rationalize the qualitative geometry (face-to-face, parallel-displaced and T-shaped) required for effective dimeric arrangements between two aromatic systems (Fig. 1a), Sanders and Hunter proposed a simple model in 1990, in which each aromatic carbon atom carries one positive *δ* charge and two negative 1/2 π charges located above and below the plane of the aromatic framework.^4–6^ Based on their model, the geometry of π-stacked assemblies is a result of competition between attractive *δ*–π interaction on one side and π–π and/or *δ*–*δ* repulsion on the other side where interplanar distances cover the 3–4 Å range. For example, the T-shaped and the parallel-displaced organizations of [benzene]_2_ dimer are more favorable than the perfect face-to-face stacking due to the significant repulsion between the two monomers found in the latter configuration (Fig. 1a). Their prediction was further supported by theoretical modeling, in which the binding energies for the face-to-face, parallel-displaced and T-shaped geometries of the benzene-based dimer are estimated to be around 1.8, 2.8 and 2.7 kcal mol^−1^, respectively.^6^ Alternatively, a face-to-face arrangement could be observed in the crystal structure of the (electron-rich mesitylene):(electron-poor hexafluorobenzene) solid adduct (Fig. 1b).^7,8^ In agreement with Sanders–Hunter's rules, one molecule of mesitylene is bound directly to one molecule of hexafluorobenzene due to maximized *δ*–π attractions, while the unavoidable repulsive contribution between the two aromatic units is minimized by charge polarization.

**Fig. 1:**
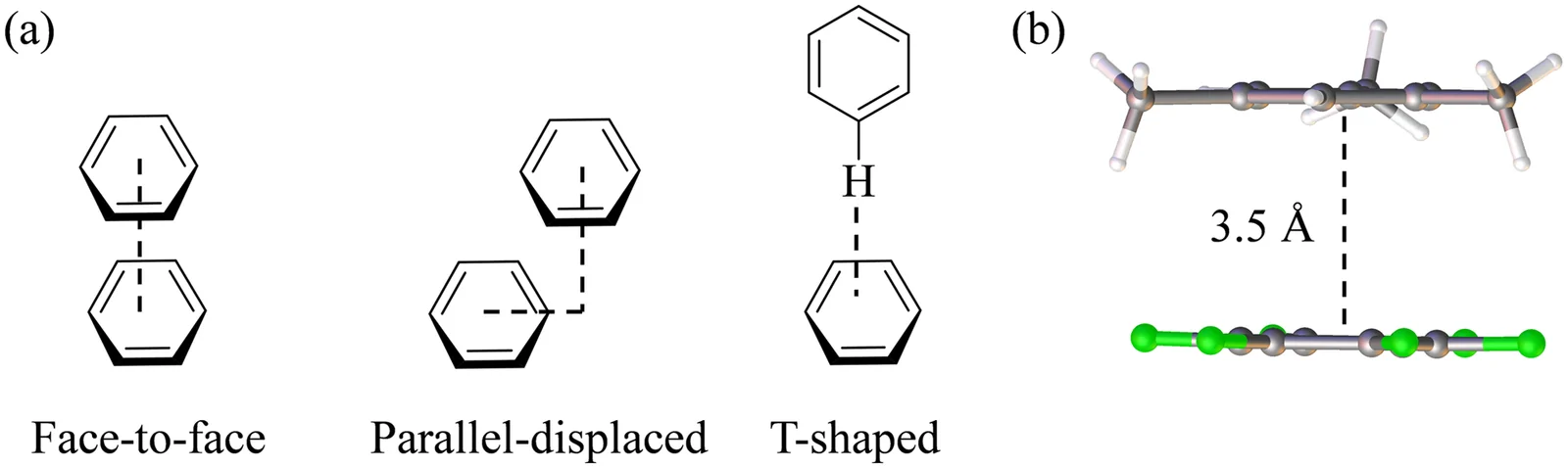
(a) Three characteristic geometries of [benzene]_2_ dimers, (b) face-to-face stacking observed between mesitylene and hexafluorobenzene.^8^

Within the frame of inter-aromatic interactions, assigned explicitly in terms of electrostatic, dispersion, desolvation, induction, and exchange-repulsion (EDDIE),^9^ planar pyrene is a well-known polycyclic aromatic luminophore for its ability to generate structureless broad and significantly red-shifted excited-dimer (so-called excimer) emission centered at around 480 nm, as compared to its well-structured monomer fluorescence in the 370–400 nm range.^10–13^ The pyrene-based excimer emission is highly sensitive to the local environment controlled by solvent polarity, medium viscosity, temperature, pH, supra/molecular confinement, light, and guest molecules, thereby enabling its applications as medical biosensors, organic chemical sensors and optoelectronic devices through the building or the quenching of the excimer emission.^14–19^ Based on the mechanism of dimeric association, the excimer emission can be categorized into two different types: dynamic and static emissions. Firstly, dynamic diffusion-controlled dimerization occurs upon photoexcitation through non-covalent π–π interactions between an excited-state pyrene monomer and another ground-state pyrene molecule. The photo-driven dimeric organization may also be formed by two different aromatic molecules, such as pyrene and 1,2-dimethylindole or pyrene and *N*,*N*-dimethylaniline, resulting in hetero-excimer (exciplex) emissions.^20,21^ Secondly, the static excimer emission originates from ground-state pre-associated dimers, in which two binding pyrene units are already forced to be in close proximity before photoexcitation and therefore the static excimer is directly generated without requiring molecular diffusion. Interestingly, some ground state pre-stacked organizations can thus reduce inter/intramolecular motion, conformational adaptability and sensitivity of the static excimer to external disturbances.^22,23^ Therefore, the interconversion between the static and the dynamic excimers through molecular design strategy for tuning its environment-sensitive photophysical behaviors has increasingly attracted the attention of organic chemists. In this context, the terimine 2,6-bis-(benzimidazol-2-yl)pyridine derivatives, for which **L0** represents the basic unit and **L1** is a decorated member of the family (Fig. 2), are well-studied extended polyaromatic ligands with programmable structural characteristics (*N*-substituted, flexible/rigid, planar/distorted).^24,25^ Upon coordination to trivalent lanthanides, the *trans* → *cis* reorganization of the terimine building block required for the chelate complexation of the entering metal induces major structural and electronic changes which have been exploited for boosting ground-state chemical reactions occurring in the second-sphere coordination.^26–29^

**Fig. 2:**
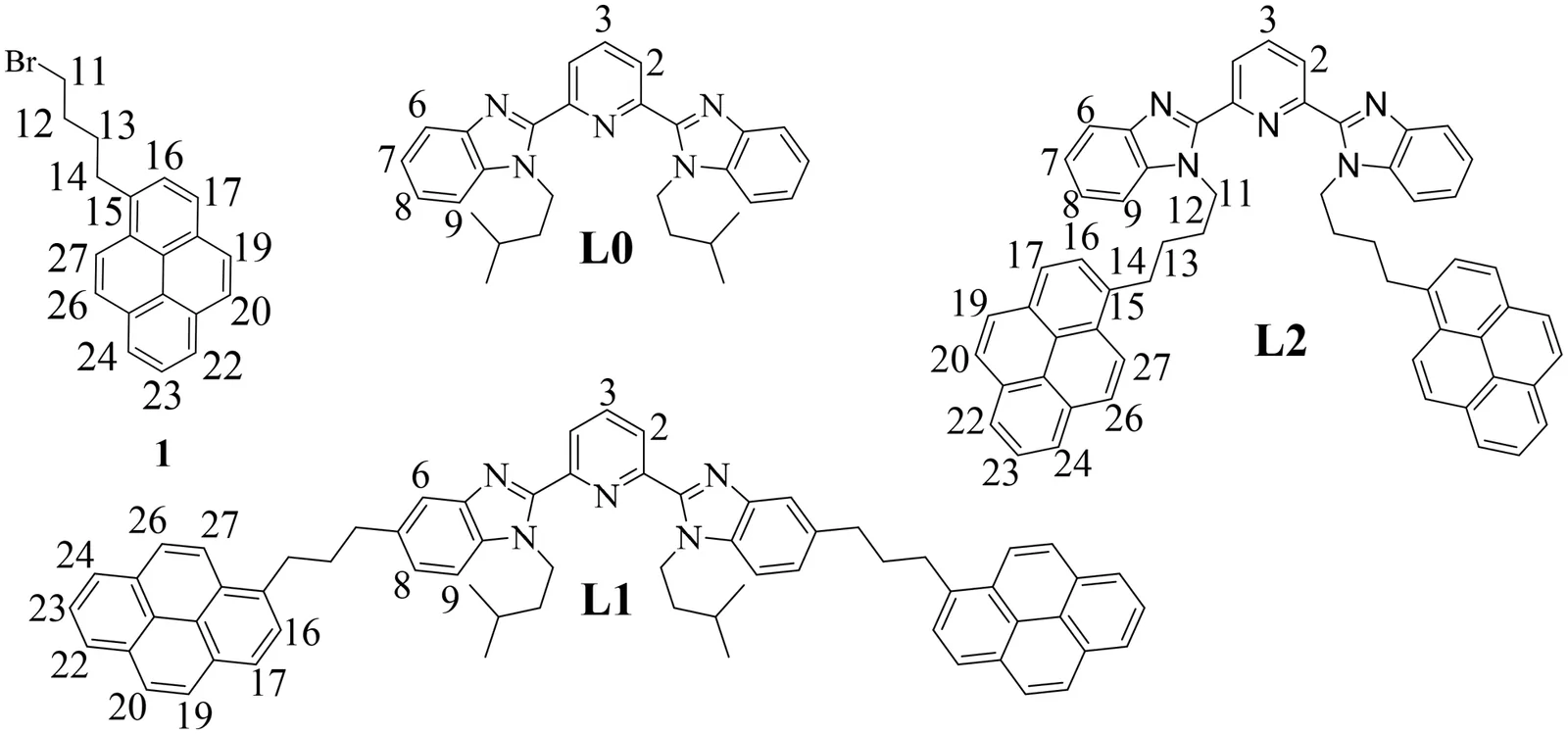
Chemical structures of target compounds considered in this report, with numbering schemes used for ^1^H NMR analysis.

In this study, we report the synthesis, characterization and photophysical properties of the pyrene–terimine ligand **L2** (Fig. 2) and of its lanthanide complexes [**L2**Ln(hfac)_3_] (Ln = Eu and Y) (Scheme 1), in which two close pyrene units are selected as fluorescence signaling side arms. The nitrogen-donor terimine core is exploited as the binding unit to catch the lanthanide ion which induces the metal-assisted supramolecular (re)organization responsible for selectively inducing ground/excited state dynamic/static dimerization processes.

**Scheme 1:**
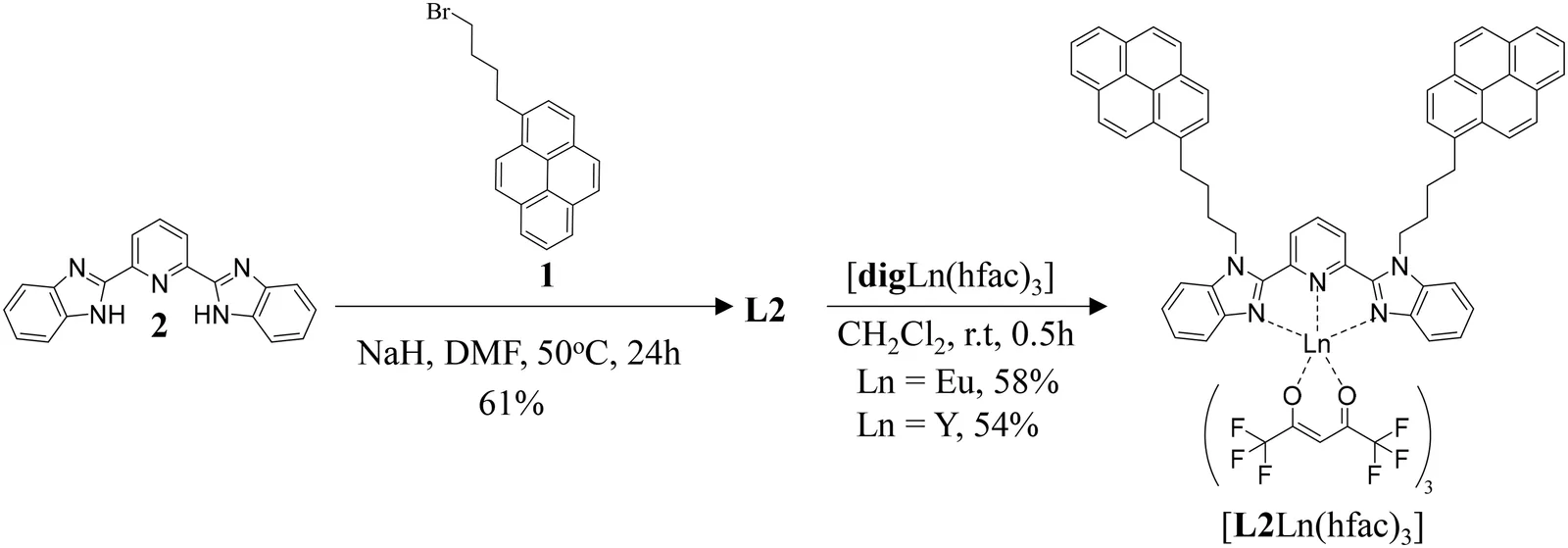
Synthesis of **L2** and its lanthanide complexes.

## 2. Results and discussion

### Synthesis and characterization of the free ligand **L2** and its lanthanide complexes

Covalent attachment of the pyrene-bearing model 1 (ref. 30) onto the 2,6-bis-(benzimidazol-2-yl)pyridine building block 2 (ref. 31) produces ligand **L2** in 61% yield (Scheme 1, left). Treatment of the terimine ligand **L2** with an excess of [**dig**Ln(hfac)_3_] (Ln = Eu (ref. 32) and Y (ref. 33) with **dig** = diglyme, and hfac = 1,1,1,5,5,5-hexafluoro-pentane-2,4-dione) in dichloromethane, followed by precipitation in pentane to remove the excess of [**dig**Ln(hfac)_3_], afford the heteroleptic [**L2**Eu(hfac)_3_] and [**L2**Y(hfac)_3_] adducts in 58% and 54% yield, respectively (Scheme 1, right). Only a single molecule of the terimine **L2** ligand could be bound to the lanthanide center in the resulting complexes thanks to a programmed control of the coordination number by using three bidentate hfac^−^ co-ligands.

Single crystals of **L2**, [**L2**Y(hfac)_3_] and [**L2**Eu(hfac)_3_] suitable for X-ray analysis (Fig. 3a and b and Fig. A2-1, A2-3, and A2-5 in SI Appendix 2) were obtained by slow evaporation of dichloromethane/*n*-hexane solutions. Experimental parameters and crystal structure analysis are detailed in SI Appendix 2. The crystal structure of **L2** (Fig. 3a, left) displays a *trans*–*trans* orientation of the two fused di-imine units with an average benzimidazole–pyridine dihedral angle of 22.82° (Table 1, entry 1) which minimizes (i) the dipole–dipole repulsion between the nitrogen lone pairs of the distal benzimidazole and the central pyridine rings and (ii) the steric hindrance between the *N*-substituent groups of the benzimidazole side arms and the aromatic proton connected at the *meta* position of the central pyridine ring. The molecular packing of **L2** displays intermolecular pairs of ground state parallel-displaced π-stacked pyrene units with an interplanar distance of 3.48 Å (Fig. 3a, right; ∼50% of the aromatic areas are overlapped corresponding to ∼8 carbon atoms). In agreement with Sanders–Hunter rules,^4^ which predict that the π–π interaction between the two neutral pyrene units is more efficient than that between the electron-rich terimine and the neutral pyrene core or between the two electron-rich terimine units, only a homoaromatic π-stacked arrangement between the two neutral pyrene units is detected in the crystal structure of the bichromophoric ligand **L2**.

**Fig. 3:**
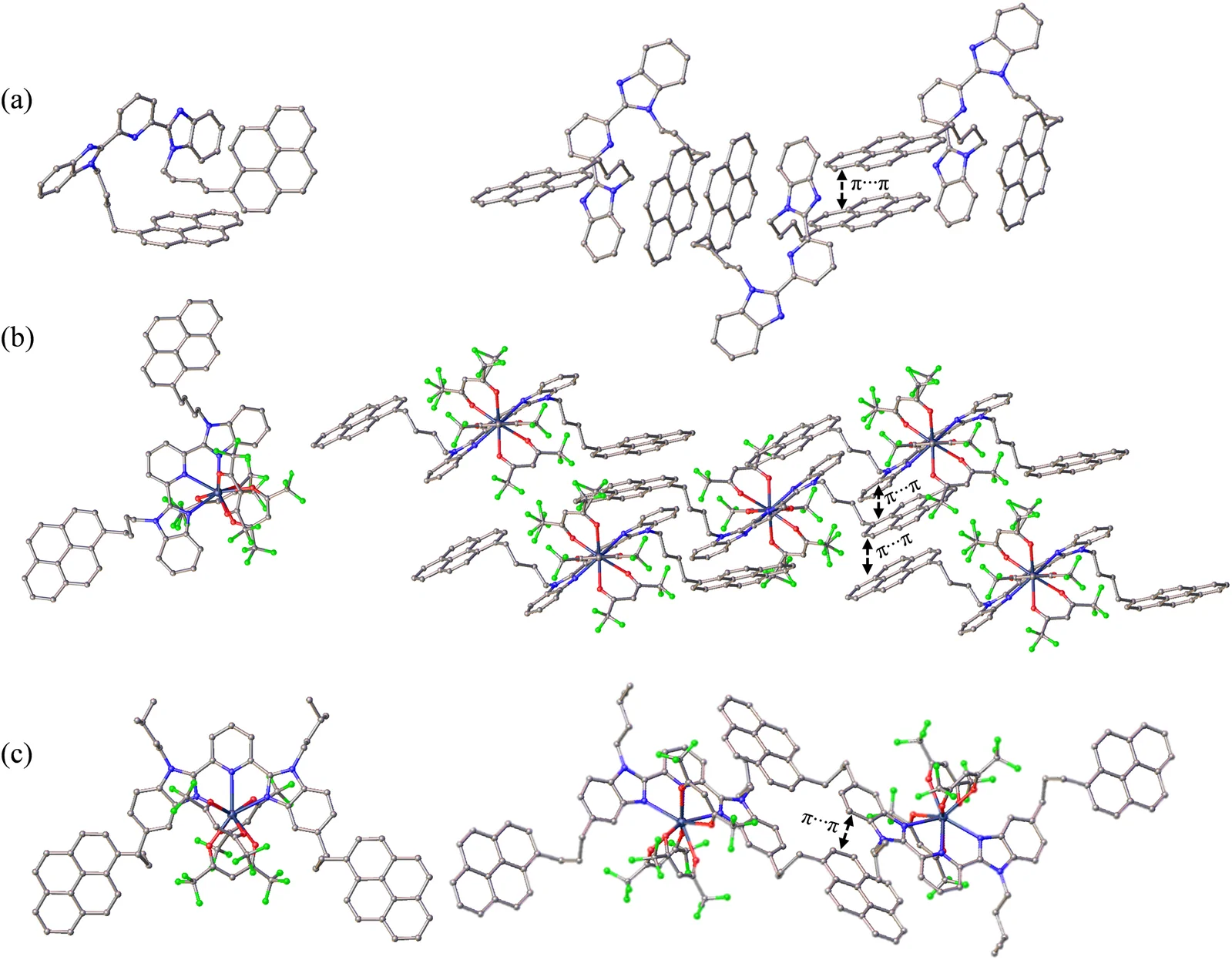
Molecular structure (left) and molecular packing (right) found in the crystal structures of (a) **L2**, (b) [**L2**Y(hfac)_3_]_3_ and (c) [**L1**Y(hfac)_3_]_3_ (CCDC 2531611).^34^ Color codes: C = gray, N = light blue, O = red, F = green, and Y = dark blue. Hydrogen atoms and solvent molecules have been omitted for clarity.

**Table 1:** Dihedral angles between the pyridine center and the benzimidazole side arms in **L2** and [**L*k***Ln(hfac)_3_] complexes (**L*k*** = **L0**–**L2**, and Ln = Eu, Y)

Entry	Compound	Dihedral angle py-bzim1/°	Dihedral angle py-bzim2/°
1	**L2**	20.84(4)	24.81(4)
2	[**L0**Eu(hfac)_3_]^24^	14.08	30.55
3	[**L1**Eu(hfac)_3_]^34^	12.92(12)	12.92(12)
4	[**L1**Y(hfac)_3_]^34^	12.12(8)	12.12(8)
5	[**L2**Eu(hfac)_3_]	4.44(10)	28.68(12)
6	[**L2**Y(hfac)_3_]	4.48(7)	27.37(8)

The binding of the trivalent lanthanide to the terimine core in the complexes [**L2**Ln(hfac)_3_] gives rise to (i) *trans*–*trans* to *cis*–*cis* conformational switching in the bound ligand **L2** in the resulting complexes due to the rotation of the benzimidazole side arms with respect to the pyridine center upon binding reaction (Fig. 3b, left, and A2–4 and A2–6) and (ii) intermolecular supramolecular rearrangement of the interaromatic π–π interactions in the solid state due to the local charge polarization induced by the bound metal (Fig. 3b, right). The molecular packing of [**L2**Y(hfac)_3_] (Fig. 3b, right) indeed reveals multiple non-covalent organizations in which a neutral pyrene unit is sandwiched between (i) a second neutral pyrene (with an average interplanar distance of 3.50 Å and an average π–π overlap of 33%, Table A2-11 in SI Appendix 2) and (ii) an electron-poor bound terimine unit (with a spacing of 3.49 Å and an average overlap of 30%, Table A2-11 in SI Appendix 2). The latter π–π interactions between the pyrene and the bound terimine cores in [**L2**Ln(hfac)_3_] (Ln = Eu and Y) complexes are promoted by the decreased electrostatic π–π repulsion resulting from the binding of the terimine unit to the lanthanide ion; this is in complete agreement with related observations reported for [**L1**Ln(hfac)_3_] complexes (Ln = Eu and Y, Fig. 3c).^34^ The [**L2**Ln(hfac)_3_] complexes also display (i) a distortion between the pyridine center and the benzimidazole side arm (bzim2) with an average dihedral angle of 28.0(10)° (entries 5 and 6 in Table 1) comparable with those found in [**L1**Ln(hfac)_3_] (entries 3 and 4 in Table 1) and [**L0**Eu(hfac)_3_] (entry 2 in Table 1) complexes, but (ii) a close-to-planar molecular geometry between the central pyridine and the second benzimidazole (bzim1; average torsion angle of 4.5(1)° gathered in entries 5 and 6 in Table 1).

The average distance between the *meta* hydrogen atom of the pyridine ring and those of the N–CH_2_ group of the adjacent benzimidazole ring in [**L2**Ln(hfac)_3_] amounts to 2.1 Å, which is smaller than the sum of two non-covalent hydrogen atomic radii (2.4 Å), indicating some steric congestion induced by the hetero-π-stacked arrangement. Importantly, the average dihedral angle between the stacked pyrene unit and the terimine core in the dimeric organization of [**L2**Ln(hfac)_3_] (12.3°, Tables A2-7 and A2-11 in SI Appendix 2) is larger than that reported for [**L1**Ln(hfac)_3_] (6.6°).^34^ This confirms an increased steric repulsion around the terimine units upon dimerization to give [**L2**Ln(hfac)_3_]_2_. It results in a competition between homo-π-stacked arrangement involving two planar pyrene units (0° dihedral angle, Tables A2-7 and A2-11) and hetero-stacked organization implying the planar pyrene and the wrapped terimine cores (dihedral angle of 12.3°) in the solid-state molecular packing of [**L2**Ln(hfac)_3_].

The qualitative ground state self-association and structural properties of ligand **L2** as well as its diamagnetic yttrium complex [**L2**Y(hfac)_3_] were also investigated in solution using NMR spectroscopy. The structural characterization and the metal-centered photophysical behaviors of the paramagnetic complex [**L2**Eu(hfac)_3_] in solution will be discussed in a subsequent publication. Let us start at diluted concentration (∼5 × 10^−3^ M) to unravel the intramolecular interactions operating in the free ligand **L2**, in its analogues **1**,^34^ and **L1**,^34^ and in the diamagnetic complexes [**L2**Y(hfac)_3_] and [**L1**Y(hfac)_3_] (dichloromethane solution; Fig. 4). Firstly, the covalent attachment of the pyrene unit onto the benzene core of the benzimidazole side arm in **L1** through aliphatic linkers does not affect the ^1^H NMR chemical shifts of the hydrogen atoms (*i.e.* H^27^) bound to the pyrene (compound 1 is taken as the reference, Fig. 4a and b). However, the connection of the pyrene unit to the nitrogen atom of the benzimidazole ring in **L2** (Fig. 4c) leads to significant upfield shifts of the pyrene aromatic protons (*i.e.* H^27^), suggesting an intramolecular non-covalent π–π interaction involving two nearby-located pyrene units where **L2** adopts a ‘folded-pyrene’ geometry (Fig. 5a, center). This analysis is further supported by the upfield shift of the aromatic protons (*i.e.* H^27^) of compound 1 upon increasing concentration due to the intermolecular π-driven pyrene–pyrene self-association (green trace in Fig. 6a).^34^

**Fig. 4:**
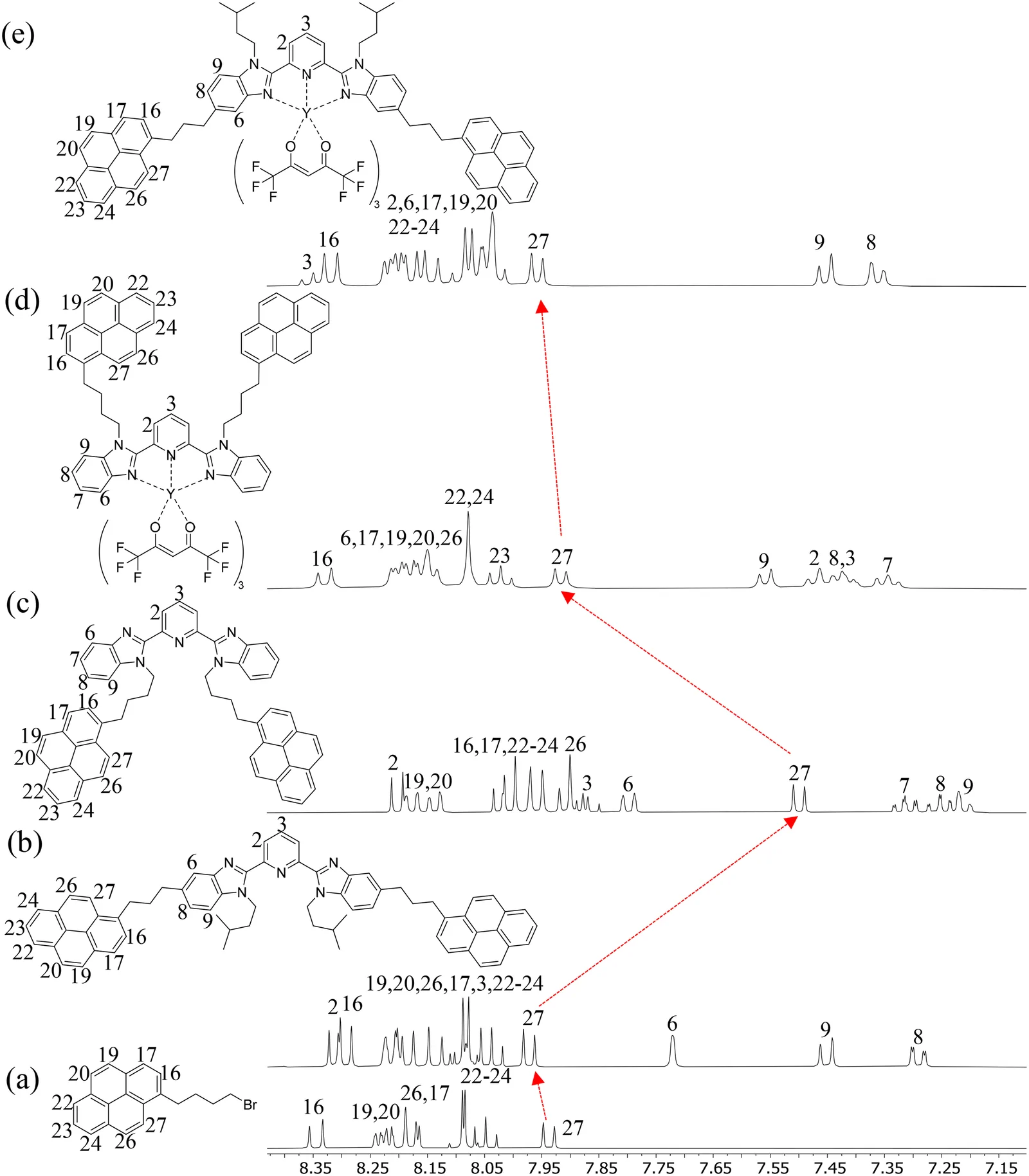
^1^H NMR spectra recorded at 5 × 10^−3^ M in CD_2_Cl_2_ solution at 298 K of (a) 1, (b) **L1**, (c) **L2**, (d) [**L2**Y(hfac)_3_], and (e) [**L1**Y(hfac)_3_].

**Fig. 5:**
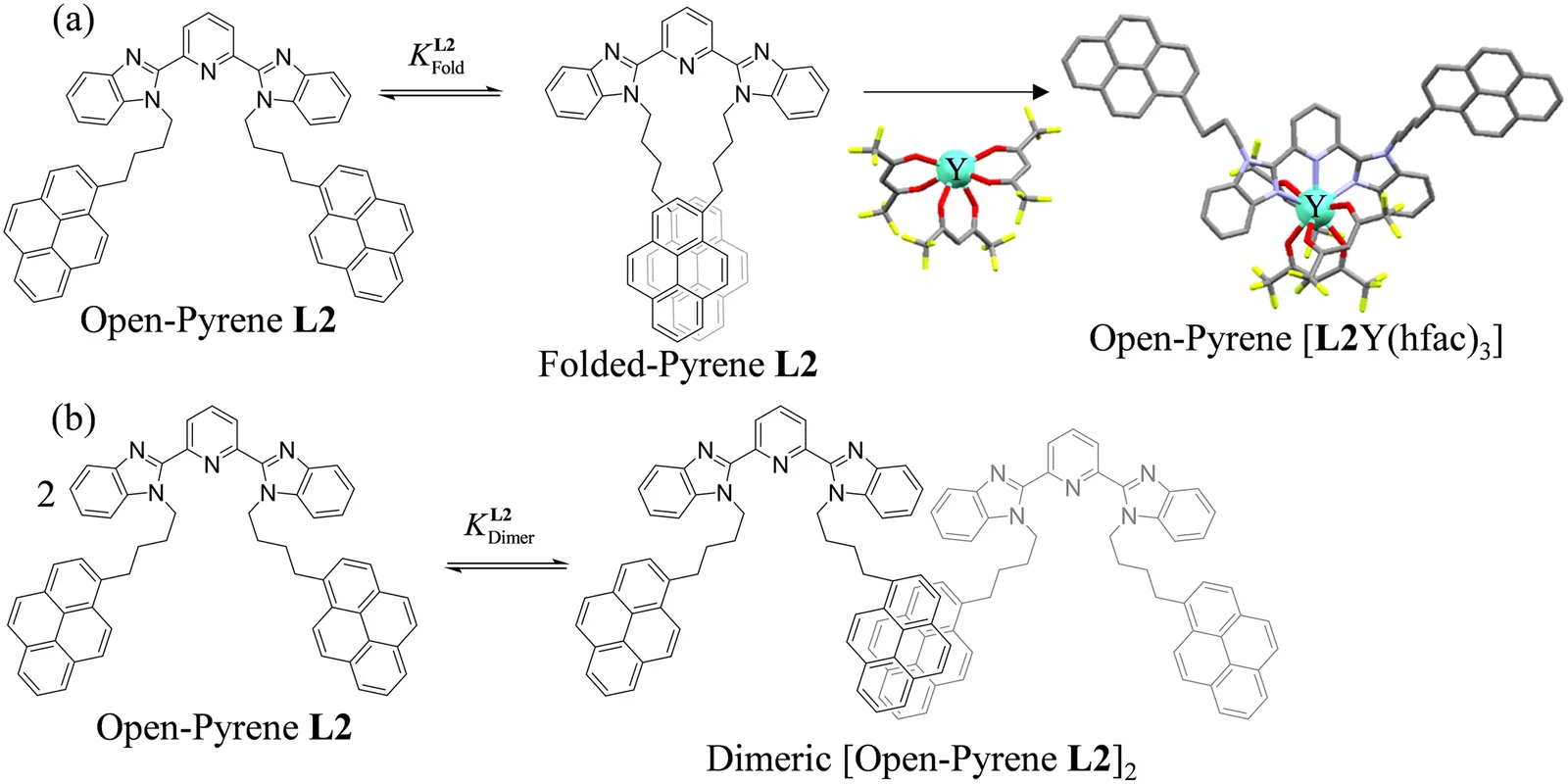
Schematic representations of (a) the intramolecular communication (left) and proposed geometric rearrangement of **L2** upon binding to the trivalent yttrium (right) and (b) the intermolecular dimeric [open-pyrene **L2**]_2_ association between the two pyrene units.

**Fig. 6:**
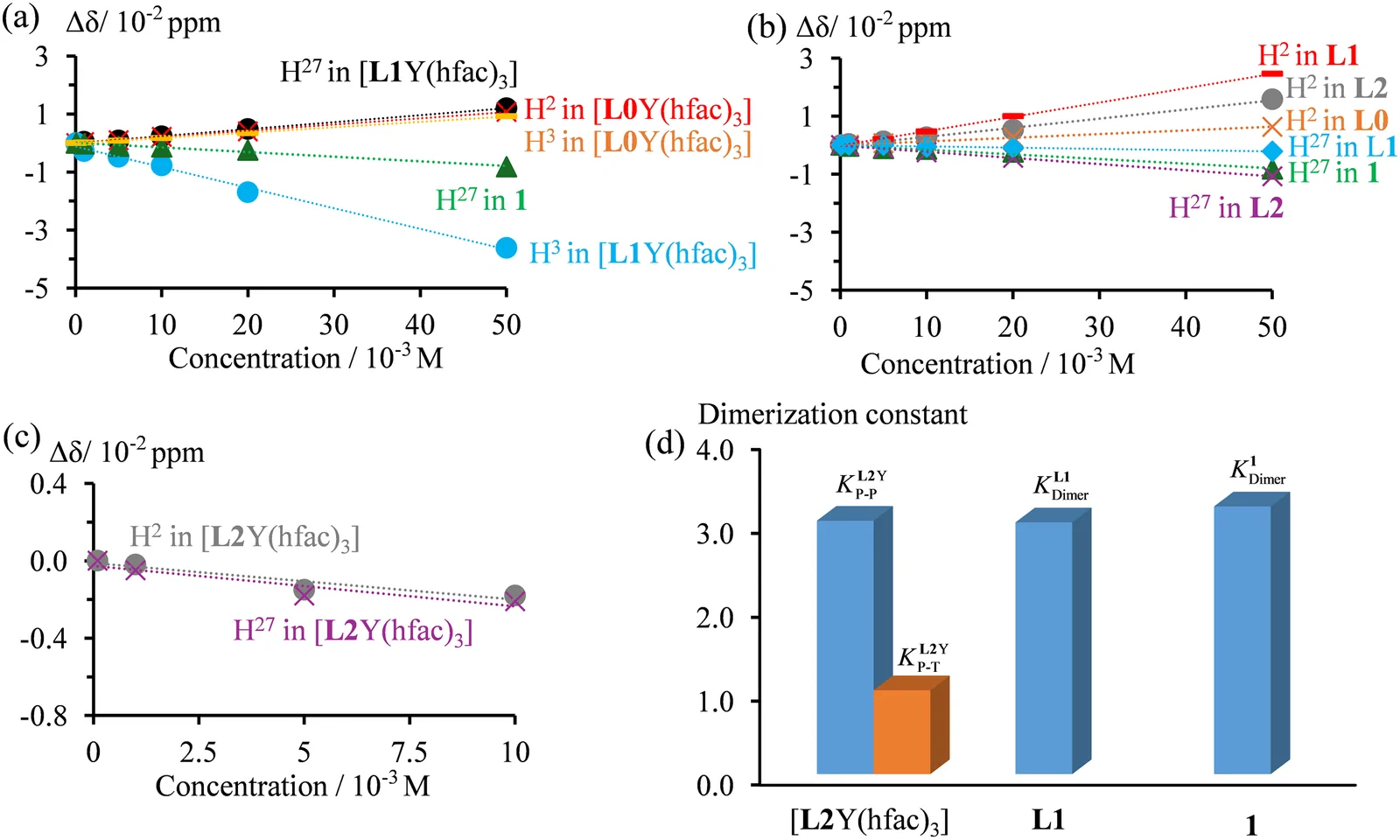
(a)–(c) Difference in chemical shifts with respect to that recorded at 10^−4^ M plotted upon increasing concentrations for 1,^34^ [**L0**Y(hfac)_3_],^34^ [**L1**Y(hfac)_3_],^34^**L2**, and [**L2**Y(hfac)_3_], (d) dimerization constants for 1,^34^**L1**,^34^ and [**L2**Y(hfac)_3_].

Secondly, the binding of the lanthanide ion to the *trans*–*trans* terimine unit in the free ligand **L2** induces a structural rearrangement to give the bound *cis*–*cis* terimine observed in the final [**L2**Ln(hfac)_3_] (Ln = Y and Eu) complexes. This can be visualized by the NOESY correlations (Fig. A1-13 and A1-19) between the protons (H^11^ and H^12^) of the aliphatic chain attached to the benzimidazole side arms and the aromatic proton H^2^ of the central pyridine ring (numbering in Fig. 2). The *trans*–*trans* to *cis*–*cis* conformational change disrupts the intramolecular π–π interaction between the two pyrene units so that the ‘folded-pyrene’ free ligand **L2** transforms into the ‘open-pyrene’ geometry upon complexation to give [**L2**Y(hfac)_3_] (Fig. 5a, right). This is ascertained by the comparable chemical shifts of the pyrene aromatic protons (*i.e.* H^27^) observed in 1 (Fig. 4a), **L1** (Fig. 4b), [**L2**Y(hfac)_3_] (Fig. 4d) and [**L1**Y(hfac)_3_] (Fig. 4e). Finally, we investigated the intermolecular interactions in the target compounds by recording the ^1^H NMR spectra at various concentrations (Fig. 6a–c, A1-20, and A1-21). The pyrene units in the free ligand **L2** and in compound 1 exhibit comparable concentration-dependent behaviors (violet and green traces in Fig. 6b). Similarly, the terimine cores in **L2** and **L0** show identical responses with increasing concentration (grey and orange traces in Fig. 6b). Altogether, these data indicate no ground state intermolecular communication between the pyrene and the tridentate units in the free ligand **L2** in complete agreement with previous observations reported for **L1** (red and blue traces in Fig. 6b).^34^

The situation is more complicated in the lanthanide adducts and we showed, in a previous report,^34^ that the [**L1**Ln(hfac)_3_] (Ln = Y and Eu) complexes displayed an intermolecular hetero-π-stacked arrangement between the pyrene and the terimine units (Fig. 3c, right), visualized in solution by the different behaviors of the chemical shifts of the aromatic protons (H^27^, H^2^ and H^3^) compared with those of the pyrene-bearing model compounds 1 (H^27^), ligand **L0** (H^2^ and H^3^) and complex [**L0**Y(hfac)_3_] (Fig. 6a). For [**L2**Y(hfac)_3_], a π-stacked competition between the pyrene–pyrene homo-interaction and the pyrene–terimine hetero-interaction is detected upon increasing concentration. Firstly, the aromatic protons of the terimine unit in [**L2**Y(hfac)_3_] (grey trace in Fig. 6c) are shifted upfield as found in [**L1**Y(hfac)_3_] (blue trace in Fig. 6a), which is the signature of a pyrene–terimine π-stacked arrangement.^34^ Secondly, the aromatic protons of the pyrene moiety in [**L2**Y(hfac)_3_] are shifted upfield (violet trace in Fig. 6c) as similarly observed for the aromatic protons of the pyrene fragments in the free ligands **L2** (violet trace in Fig. 6b) and **L1** (blue trace in Fig. 6b) and in the model pyrene 1 (green trace in Fig. 6b). This is taken as the signature for a π-stacked pyrene–pyrene organization. The latter observations confirm the coexistence of two independent hetero- and homo-stacked organizations in the bichromophoric adduct [**L2**Y(hfac)_3_], mimicking its supramolecular π-packing in the solid state (Fig. 3b, right).

### Thermodynamic behavior of **L2** with [Y(hfac)_3_] container in dichloromethane

The thermodynamic association constant leading to [**L2**Y(hfac)_3_] was investigated by ^1^H NMR titration (Fig. 7) of the nitrogen-donor terimine **L2** host with the [**dig**Y(hfac)_3_] guest according to eqn (1).

1 [**dig**Y(hfac)_3_] + **L2** ⇌ [**L2**Y(hfac)_3_] + **dig** *β*^**L2**,Y^_1,1,exch_

The ^1^H NMR titration is conducted in dichloromethane solution at 293 K using an excess of diglyme (|**dig**|_tot_ = 0.142 M) to stabilize the activity coefficients at millimolar concentrations during the host–guest titration. This transforms the exchange constant *β*^**L2**,Y^_1,1,exch_ (eqn (1)) into the conditional binding constant *β*^**L2**,Y^_1,1,cond_ (eqn (2)).^35^2
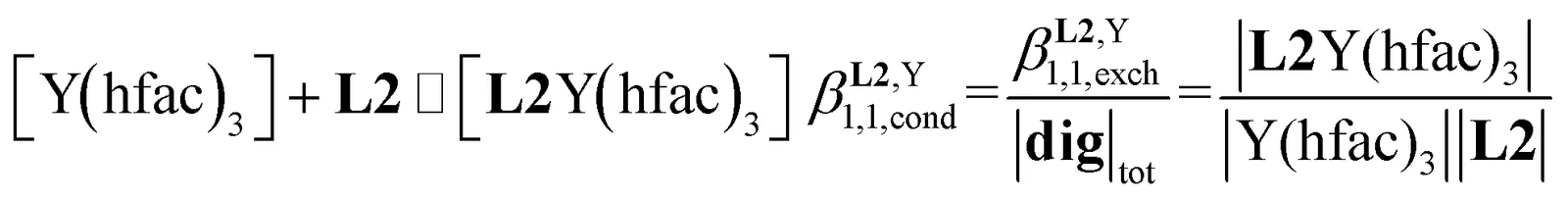
3
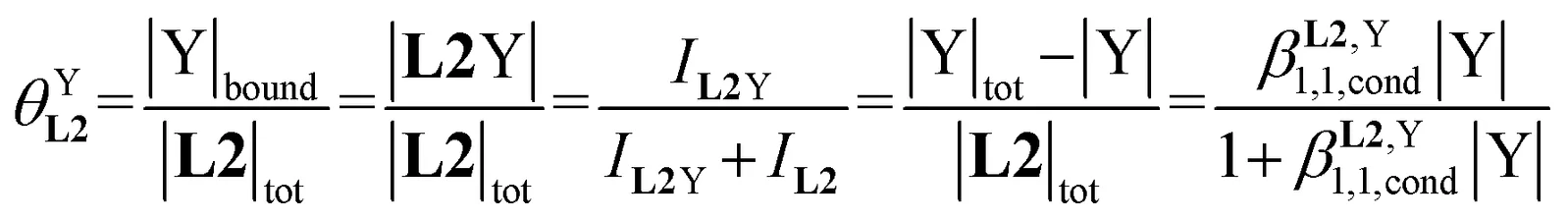
In practice, the ^1^H NMR signals of the protons are detected under the slow exchange regime on the NMR time scale at each titration point (Fig. 7), which provides reliable integrations for the same proton bound to the coordinated (*I*_**L2**Y_) and to the free (*I*_**L2**_) ligand. The associated occupancy factors 
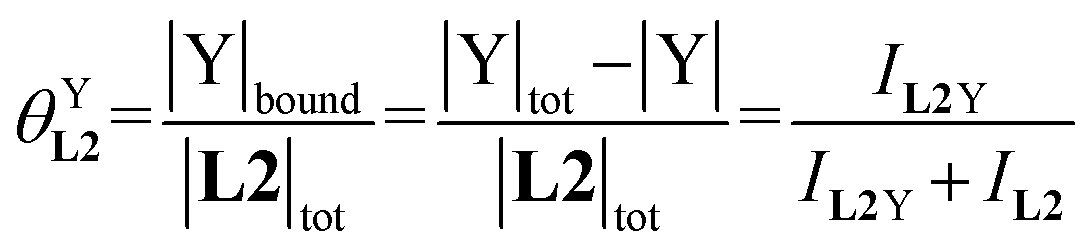
 (eqn (3), left) and free concentrations of the trivalent lanthanide guest in solution |Y| = |Y|_tot_ − *θ*^Y^_**L2**_|**L2**|_tot_ are computed at each titration point and plotted as experimental binding isotherms (black diamonds in Fig. 8a). The subsequent non-linear least-square fitting of *θ*^Y^_**L2**_*versus* |Y| with the help of eqn (3) (right) provides the conditional stability constant *β*^**L2**,Y^_1,1,cond_ gathered in Table 2. Since *β*^**L2**,Y^_1,1,exch_ = *β*^**L2**,Y^_1,1,cond_·|**dig**|_tot_ with |**dig**|_tot_ = 0.142 M, the exchange constant *β*^**L2**,Y^_1,1,exch_ is easily deduced (Table 2).

**Fig. 7:**
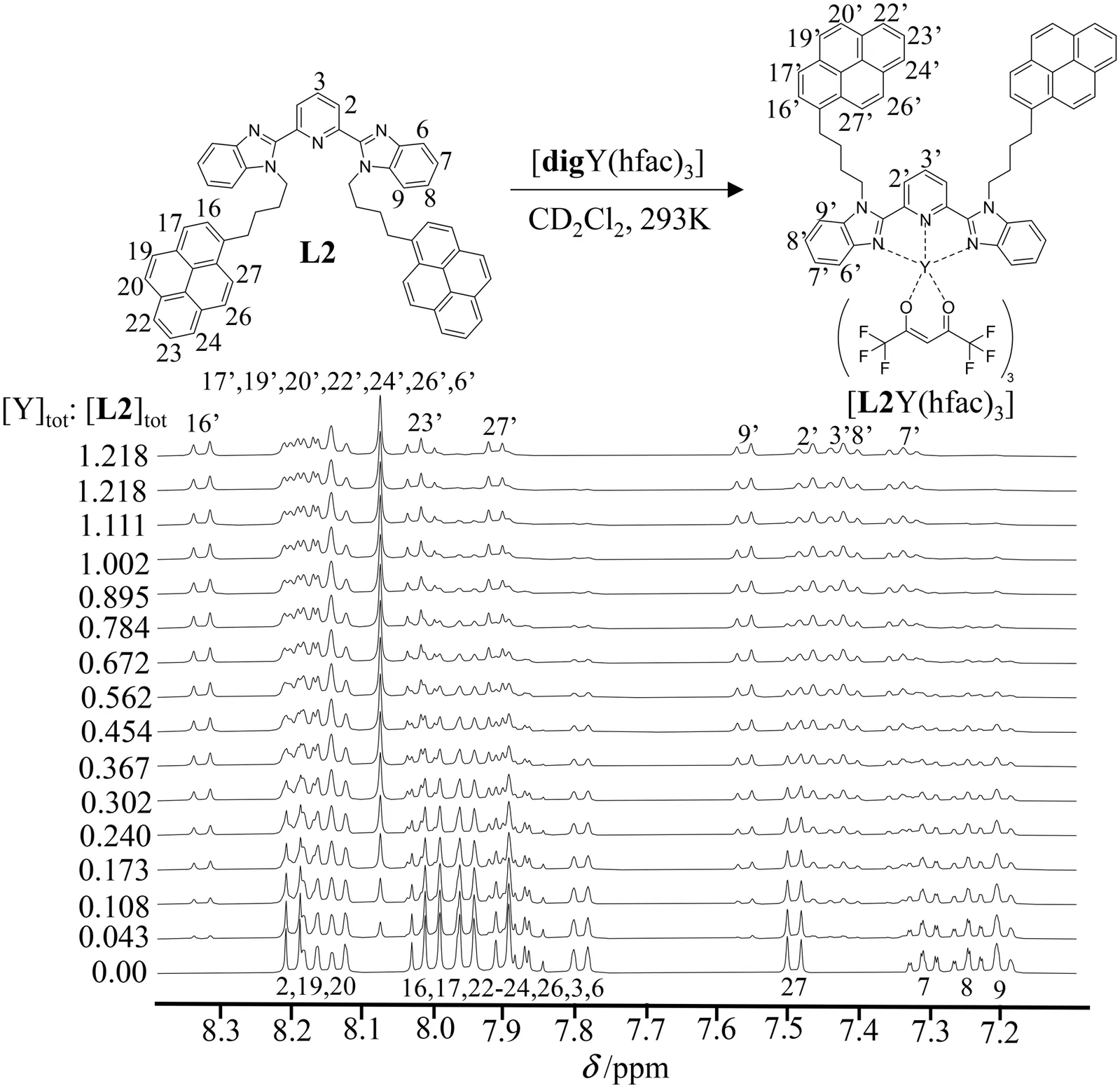
^1^H NMR titration of **L2** (3.37 mM) with [**dig**Y(hfac)_3_] + 0.142 M diglyme at 293 K.

**Fig. 8:**
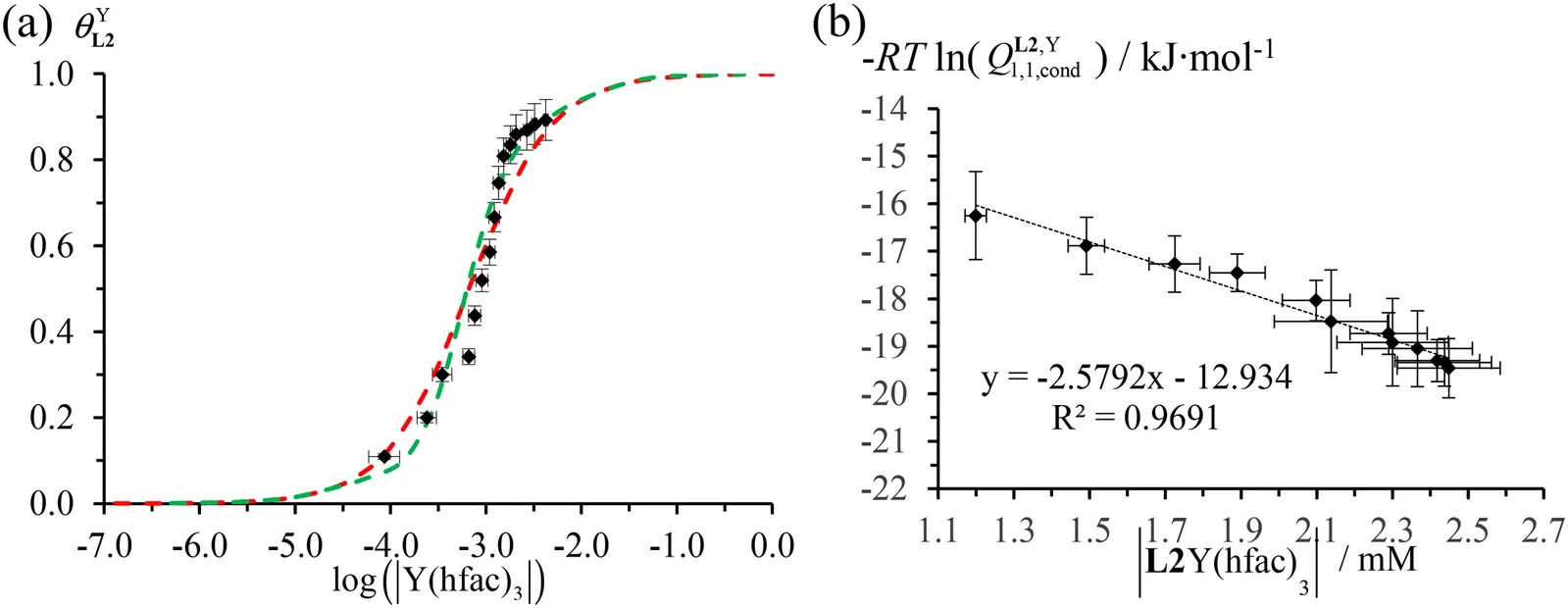
(a) Experimental (black diamonds) and fitted (dashed traces) binding isotherms for the titration of **L2** with [**dig**Y(hfac)_3_] in CD_2_Cl_2_ + 0.142 M diglyme at 293 K, and (b) plot of −*RT* ln(*Q*^**L2**,Y^_1,1,cond_) with respect to the concentration of [**L2**Y(hfac)_3_].

**Table 2:** Thermodynamic parameters determined from the titration of **L2** with [**dig**Y(hfac)_3_] in CD_2_Cl_2_ + 0.142 M dig at 293 K

*β* ^**L2**,Y^_1,1,cond_ a	*β* ^**L2**,Y^_1,1,exch_ a	Δ*G*^**L2**,Y,∞^_1,1,cond_/kJ mol^−1^	Δ*G*^**L2**,Y,S^_1,1,cond_/kJ mol^−1^
1502(179)	213(25)	−15.9(5)	−1.2(3) × 10^3^

a At 298 K with the reference state being fixed at *c*^θ^ = 1 M and using ideal activity coefficients *γ*_*i*_ = 1.^35–37^

However, the binding isotherm rebuilt using the association constant *β*^**L2**,Y^_1,1,cond_ and eqn (3) (red trace in Fig. 8a) displays some systematic deviations from the experimental data (black diamonds in Fig. 8a) due to the residual variation in the activity coefficients *γ*_*i*_ occurring in non-ideal organic solution according to eqn (4), where *c*^θ^ = 1 M stands for the concentration of the reference state.^35^ Eggers proposed eqn (5) to catch the change in activity coefficients during the binding process.^36,37^4
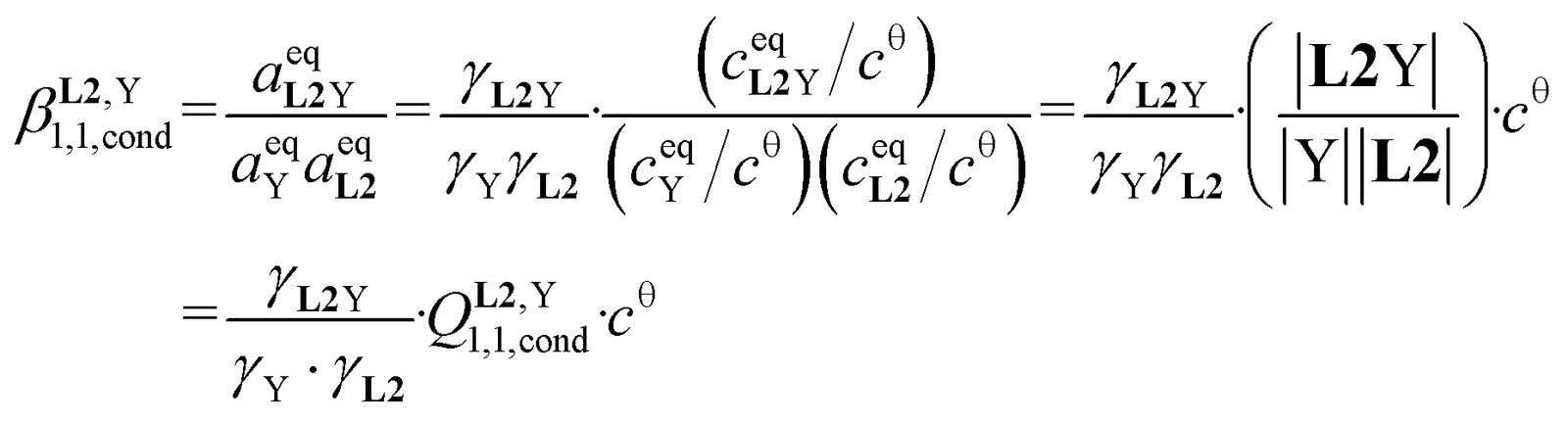

5 −*RT* ln(*Q*^**L2**,Y^_1,1,cond_) = −*RT* ln(*β*^**L2**,Y,∞^_1,1,cond_) + Δ*G*^**L2**,Y,S^_1,1,cond_·(|**L2**Y|/*c*^θ^)

In agreement with eqn (5), the plot of −*RT* ln(*Q*^**L2**,Y^_1,1,cond_) with respect to the concentration of the final complex |**L2**Y| is close to linear (Fig. 8b) and affords free energy change at infinite dilution Δ*G*^**L2**,Y,∞^_1,1,cond_ = −*RT* ln(*β*^**L2**,Y,∞^_1,1,cond_) and solvation free energy change Δ*G*^**L2**,Y,S^_1,1,cond_ (Table 2). Satisfying occupancy factors *θ*^Y^_**L2**_ could be then rebuilt with eqn (6) (green trace in Fig. 9a).6
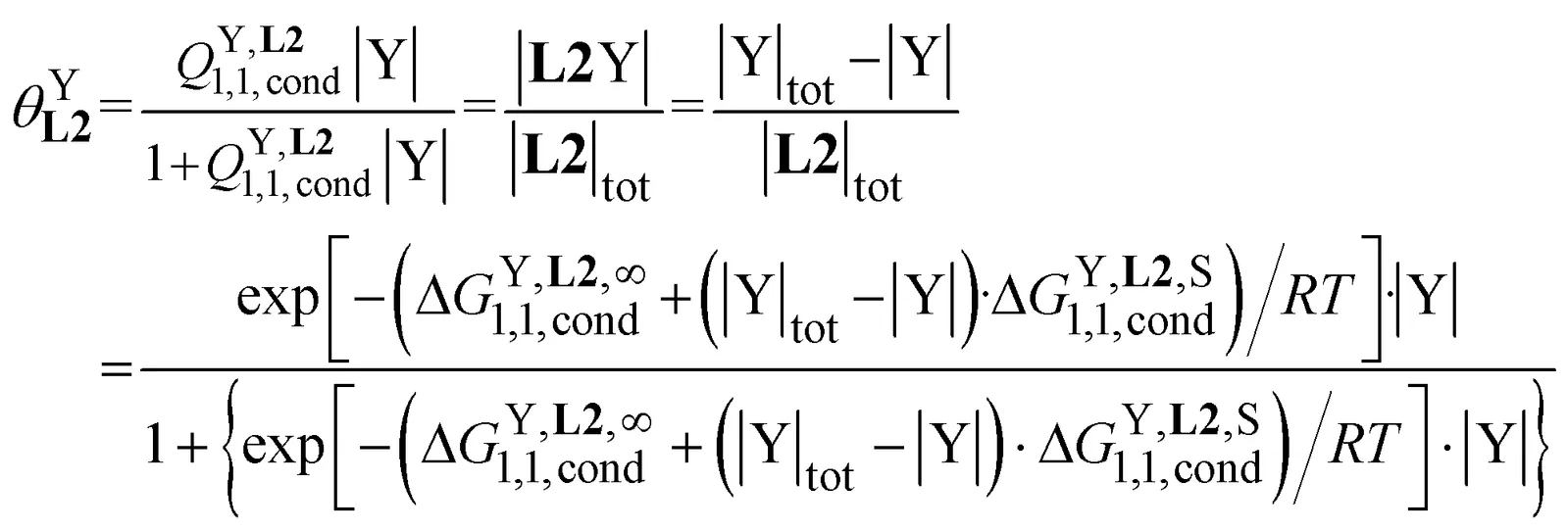
Finally, considering *β*^**L2**,Y^_1,1,exch_ = 213 (Table 2), one can estimate that 94% of the ligand exists as bound in the [**L2**Y(hfac)_3_] adduct at the total concentrations in the 5 × 10^−6^ to 10^−2^ M range. Ligand decomplexation can be therefore safely neglected upon considering the photophysical measurements and the subsequent thermodynamic analysis of π-stacking interactions.

**Fig. 9:**
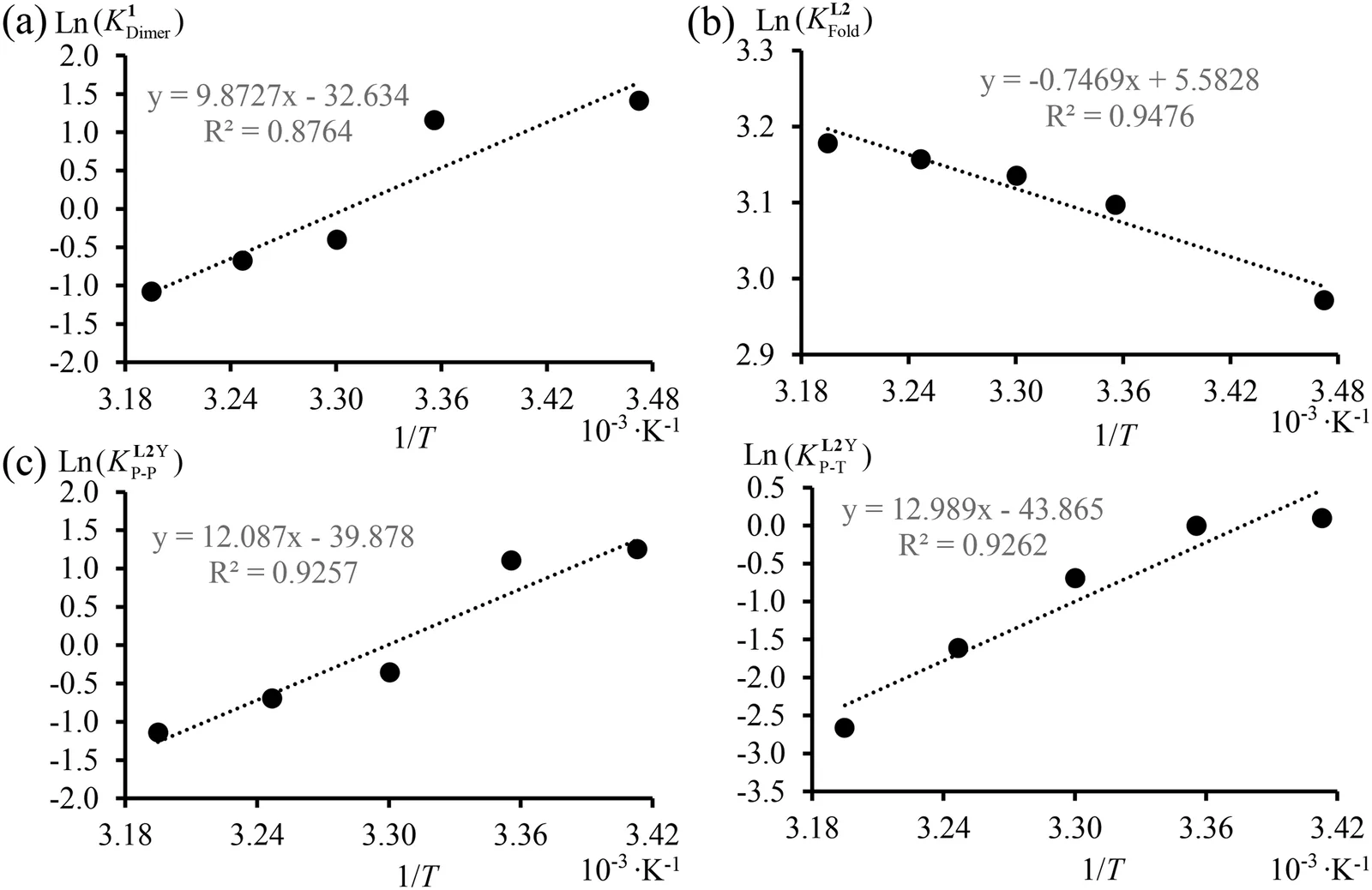
Van't Hoff plot for the π-stacking process of (a) compound 1, (b) the free ligand **L2**, and (c) the complex [**L2**Y(hfac)_3_].

### Thermodynamic parameters of the intramolecular and intermolecular π–π interactions in dichloromethane

The association constant *K*^**1**^_Dimer_ characterizing the non-covalent pyrene–pyrene dimerization of compound 1 to give [1]_2_ (eqn (7)) is determined by concentration-dependent ^1^H NMR titrations at various temperatures (SI Appendix 3) and using eqn (8),^38^ in which *δ*^**1**^_M_ and *δ*^**1**^_D_ correspond to the chemical shifts measured for the free monomer at highly diluted concentration (10^−4^ M) and for the pure dimer, respectively. *δ*^**1**^_obs_ and |1|_tot_ are the observed experimental chemical shift and total concentration of the substrate at each titration point, respectively.7
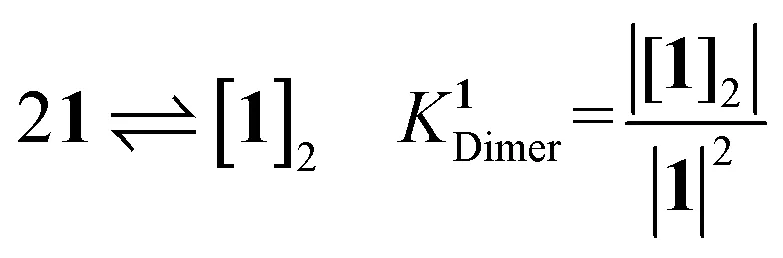
8
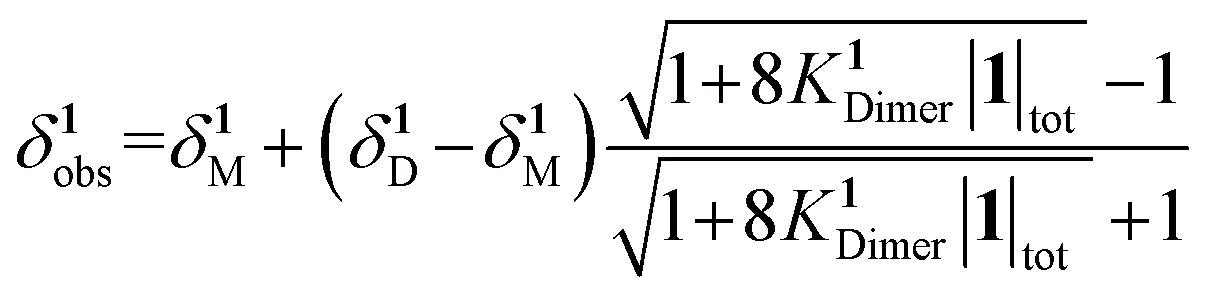
Non-linear least-squares fitting of *δ*^**1**^_obs_ recorded for H^27^ at various |1|_tot_ and using eqn (8) provides the aggregation constants *K*^**1**^_Dimer_ and the chemical shifts of the pure dimer *δ*^**1**^_D_ (Table 3, entry 1). Repeating this procedure at various temperatures is collected in Table A3-1 (SI Appendix 3).

**Table 3:** Thermodynamic parameters determined by ^1^H NMR chemical shift change of H^27^ upon dilution in dichloromethane solution

Compound	Aggregation constant^a^	Δ*H*/kJ mol^−1^	Δ*S*/J K^−1^ mol^−1^	EM
1	*K* ^**1**^_Dimer_ = 3.2(1)^b^	−82(18)	−271(59)	—
**L2**	*K* ^**L2**^_Fold_ = 22.1(4)	6.2(8)	46(3)	7
[**L2**Y(hfac)_3_]	*K* ^**L2**Y^_P–P_ = 3.0(1)	−100(16)	−332(54)	—
	*K* ^**L2**Y^_P–T_ = 1.01(8)	−108(18)	−365(58)	—

a At 298 K with the reference state being fixed at *c*^θ^ = 1 M and using ideal activity coefficients *γ*_*i*_ = 1.^35–37^

b Obtained from ^1^H NMR data reported in our previous study.^34^

The van't Hoff plot of Ln(*K*^**1**^_Dimer_) *versus* 1/*T* (Fig. 9a) according to eqn (9) results in both negative enthalpy Δ*H*^**1**^_Dimer_ and entropy Δ*S*^**1**^_Dimer_ (Table 3). The pyrene–pyrene dimerization of compound 1 is thus an enthalpically driven process limited by a considerable entropic cost accompanying the intermolecular π–π stacked organization of two molecules of 1. This implies that the dimerization of 1 can be boosted by lowering the reaction temperature to compensate for the entropy loss.9
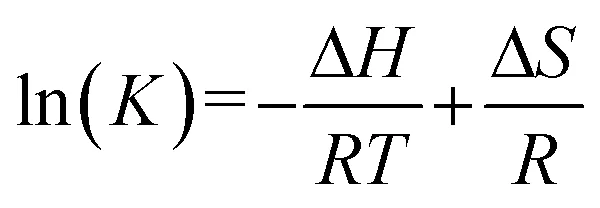
Switching to extended **L2**, in which two pyrenes 1 are now connected to a central terimine 2,6-bis-(benzimidazol-2-yl)pyridine unit, an intramolecular cyclic-like organization providing folded-pyrene **L2** (F) pertinent to *K*^**L2**^_Fold_ = |F|/|O| (Fig. 5a) competes with an intermolecular dimerization transforming the open monomer (O) into a dimer D = [open-pyrene **L2**]_2_, characterized by *K*^**L2**^_Dimer_ = |D|/|O|^2^ (Fig. 5b). Both equilibria operate under a fast-exchange regime on the NMR time scale and the concentration-dependent ^1^H NMR titrations (SI Appendix 3) display an observed experimental chemical shift *δ*^**L2**^_obs_ which corresponds to a weighted average of the chemical shifts of three contributing components: (i) the free (open) monomer *δ*^**L2**^_O_, (ii) the folded monomer *δ*^**L2**^_F_ and (iii) the dimer *δ*^**L2**^_D_ according to eqn (10).10
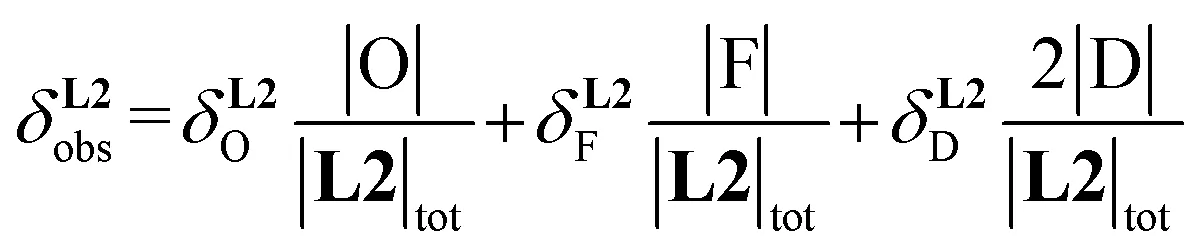
|O| and |F| correspond to the concentration of the open-pyrene monomer and the folded-pyrene monomer **L2**, respectively, |D| stands for the concentration of the dimeric form [open-pyrene **L2**]_2_ and |**L2**|_tot_ is the total concentration of ligand at each titration step. Since |F| = *K*^**L2**^_Fold_|O| and |D| = *K*^**L2**^_Dimer_|O|^2^, eqn (10) can be transformed into eqn (11).11
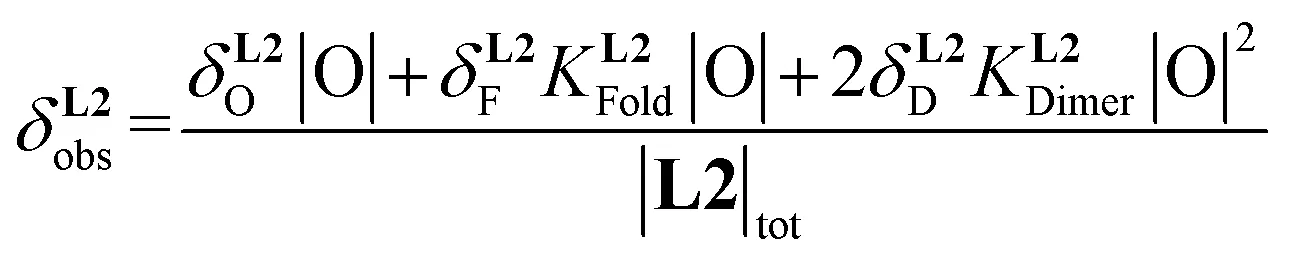
From the mass balance |**L2**|_tot_ = |O| + |F| + 2|D| = |O| + *K*^**L2**^_Fold_|O| + 2*K*^**L2**^_Dimer_|O|^2^, the resolution of the quadratic expression provides eqn (12) for determining the concentration |O|.12
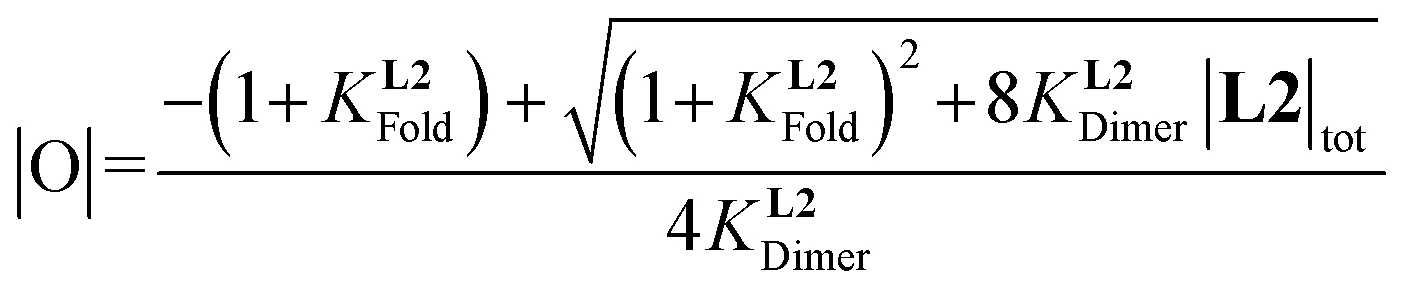
Some reasonable assumptions consider that the intermolecular pyrene–pyrene process occurring in **L2** is like the intermolecular pyrene–pyrene dimerization characterized for compound 1 (Table 3), and one can write *K*^**L2**^_Dimer_ = *K*^**1**^_Dimer_, *δ*^**L2**^_O_ = *δ*^**1**^_M_, and *δ*^**L2**^_D_ = *δ*^**1**^_D_. The subsequent non-linear least-squares fit of *δ*^**L2**^_obs_*versus K*^**L2**^_Fold_ and *δ*^**L2**^_F_ for H^27^ with the help of eqn (11) and (12) provides the searched intramolecular aggregation constant together with the chemical shift of the pure dimer listed in Tables 3 and A3-6 (SI Appendix 3).

The ratio *K*^**L2**^_Fold_/*K*^**L2**^_Dimer_ corresponds to the concept of effective molarity (EM) depicted in eqn (13), which represents the limit concentration of **L2** below which the intramolecular π-stacked arrangement is more favorable than the intermolecular dimerization (Tables 3 and A3-6 in SI Appendix 3).^39,40^13
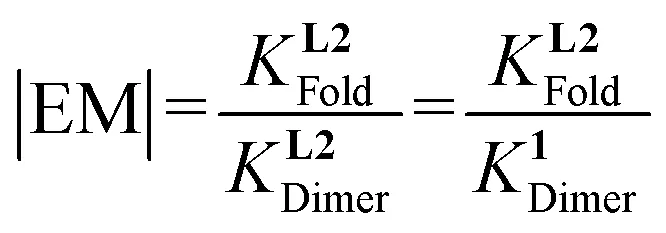
A high value of EM indicates that the intramolecular cyclic-like organization found in the folded-pyrene **L2** dominates the linear dimerization process. Using *K*^**L2**^_Dimer_ = *K*^**1**^_Dimer_ = 3.2 and *K*^**L2**^_Fold_ = 22.1 at 298 K provides EM = 7 (Table 3), in agreement with negligible dimerization at accessible concentrations. One concludes that **L2** exists almost exclusively under the form of the folded-pyrene conformer in dichloromethane solution in the 10^−2^–5 × 10^−6^ M concentration range (Table 4).

**Table 4:** Ligand speciation (%) of the open-pyrene **L2**, the folded-pyrene **L2** and the dimer [open-pyrene **L2**]_2_ computed at various concentrations in CD_2_Cl_2_ at 298 K

|**L2**|_tot_/M	10^−2^	10^−3^	10^−4^	10^−5^	5 × 10^−6^
Open-pyrene **L2**/%	4.33	4.33	4.33	4.33	4.33
Folded-pyrene **L2**/%	95.66	95.67	95.67	95.67	95.67
Dimer [open-pyrene **L2**]_2_/%	0.011	0.00	0.00	0.00	0.00

The van't Hoff plot of Ln(*K*^**L2**^_Fold_) *versus* 1/*T* (Fig. 9b) according to eqn (9) yields both positive enthalpy Δ*H*^**L2**^_Fold_ = 6.2(8) kJ mol^−1^ and entropy Δ*S*^**L2**^_Fold_ = 46(3) J mol^−1^ K^−1^ (Table 3). The expected enthalpic gain accompanying the pyrene–pyrene interaction is probably overcome by some structural reorganization of the polyaromatic bridge. The global entropically-driven process is tentatively assigned to a considerable release of weakly coordinated solvent molecules upon π–π interactions.

Focusing now on the bound ligand in the yttrium complex [**L2**Y(hfac)_3_], which is quantitatively formed in solution (see section above), one expects a competition between the homo-π-stacked pyrene–pyrene arrangement (P–P) and the hetero-π-stacked pyrene–terimine (P–T) packing in [**L2**Y(hfac)_3_], both corresponding to intermolecular dimerization processes. The observed chemical shift *δ*^**L2**Y^_obs_ is therefore considered to be a weighted average of the chemical shifts of three species: (i) the free monomer *δ*^**L2**Y^_M_, the value of which is determined at high dilution for [**L2**Y(hfac)_3_], (ii) the homo-dimer *δ*^**L2**Y^_P–P_ and (iii) the hetero-dimer *δ*^**L2**Y^_P–T_ according to eqn (14). |M| and |**L2**Y|_tot_ correspond to the concentration of the monomer form and the total concentration of [**L2**Y(hfac)_3_], respectively, and *K*^**L2**Y^_P–P_ Ch *K*^**L2**Y^_P–T_ denote the association constant for the pyrene–pyrene and the pyrene–terimine dimerization, respectively.14
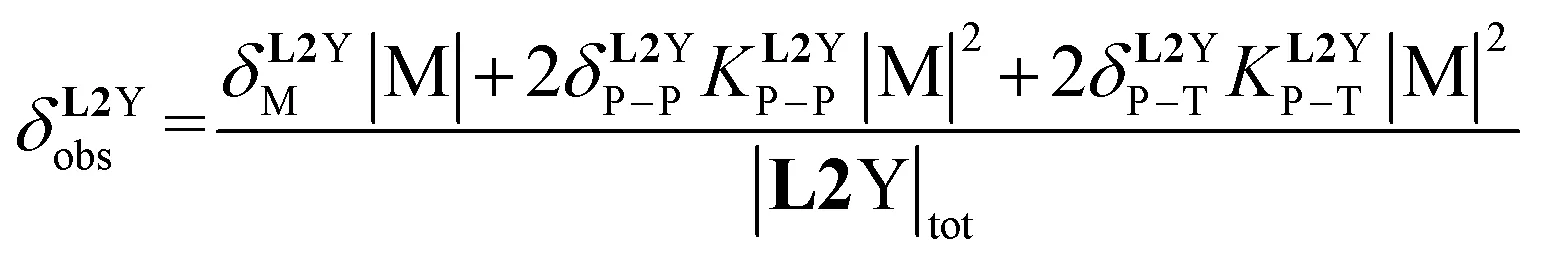
From the mass balance |**L2**Y|_tot_ = |M| + 2|P–P| + 2|P–T| = |M| + 2*K*^**L2**Y^_P–P_|M|^2^ + 2*K*^**L2**Y^_P–T_|M|^2^, in which |P–P| and |P–T| are the concentrations of the pyrene–pyrene and the pyrene–terimine dimers, respectively, the concentration of monomer |M| can be calculated at each titration step with the help of eqn (15).15
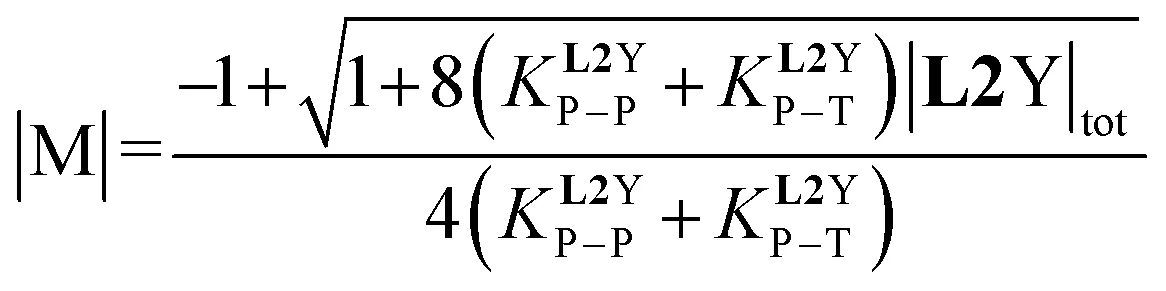
The non-linear least-squares fitting of *δ*^**L2**Y^_obs_*versus K*^**L2**Y^_P–P_, *K*^**L2**Y^_P–T_, *δ*^**L2**Y^_P–P_ and *δ*^**L2**Y^_P–T_ for H^27^ with the help of eqn (14) and (15) provides the homo- and the hetero-dimerization constants, together with the chemical shifts of the pure dimers collected in Tables 3 and A3-12. One can note that [**L2**Y(hfac)_3_] displays a larger dimerization constant for the homo-packing between two planar pyrene units than for the hetero-organization between the planar pyrene and the twisted terimine cores (*K*^**L2**Y^_P–P_ > *K*^**L2**Y^_P–T_) due to the steric congestion around the non-planar terimine moiety (Table 3 and Fig. 6d). Moreover, the comparable pyrene–pyrene dimerization constants found for 1, **L1** and [**L2**Y(hfac)_3_] (Fig. 6d) support our reliable non-linear least-squares fittings. Taking *K*^**L2**Y^_P–P_ = 3.02 and *K*^**L2**Y^_P–T_ = 1.01 at 298 K in Table 3, the percentage of monomer |M| can be estimated with eqn (15) at various concentrations, from which the amounts of dimers P–P-[**L2**Y(hfac)_3_]_2_ (|P–P| = *K*^**L2**Y^_P–P_|M|^2^) and P–T-[**L2**Y(hfac)_3_]_2_ (|P–T| = *K*^**L2**Y^_P–T_|M|^2^) are easily calculated and collected in Table 5.

**Table 5:** Amount (%) of the monomer [**L2**Y], the dimer P–P-[**L2**Y]_2_ and the dimer P–T-[**L2**Y]_2_ at various concentrations in CD_2_Cl_2_ at 298 K

|**L2**Y|_tot_/M	10^−2^	10^−3^	10^−4^	10^−5^	5 × 10^−6^
Monomer [**L2**Y] (%)	93.0	99.2	99.9	100	100
Dimer P–P-[**L2**Y]_2_ (%)	5.2	0.6	0.1	0	0
Dimer P–T-[**L2**Y]_2_ (%)	1.8	0.2	0.0	0	0

We can thus safely conclude that no significant ground-state excimer-like dimerization of [**L2**Y(hfac)_3_] occurs in the 5 × 10^−6^ to 10^−2^ M range. The van't Hoff plots of Ln(*K*^**L2**Y^_P–P_) and Ln(*K*^**L2**Y^_P–T_) *versus* 1/*T* (Fig. 9c) according to eqn (9) provide both negative enthalpy and entropy values (Table 3).

### Photophysical behaviors of the free ligand **L2** and its yttrium complex [**L2**Y(hfac)_3_]

The UV-vis absorption and emission spectra of the free ligand **L2**, which mainly exists in its folded monomeric form in the ground state (Table 4), and of its yttrium complex [**L2**Y(hfac)_3_], also mainly found in the open monomeric form in the ground state (Table 5 and Fig. 5a, right) are recorded in degassed dichloromethane solution at room temperature and collected in Fig. 10. The broad absorption band attributed to the terimine core, as ascertained in **L0** (orange trace in Fig. 10a, left), is also detected in the bichromophoric ligand **L2** (black trace in Fig. 10a, left) but combined with the absorption profile of the tethered pyrene unit which exhibits the well-known spin-allowed 
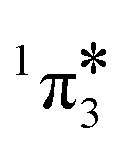
 ← ^1^π (267–279 nm), 
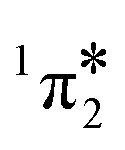
 ← ^1^π (328–345 nm), and 
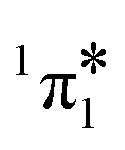
 ← ^1^π (376 nm) transitions, as ascertained by the pyrene model 1 (blue trace in Fig. 10a, left).^34^ The binding of the [Y(hfac)_3_] container to the terimine unit from the free ligands **L0** and **L2** leads to the *trans*–*trans* to *cis*–*cis* conformational rearrangement in the resulting complexes [**L0**Y(hfac)_3_] and [**L2**Y(hfac)_3_], giving rise to the detection of the red-shifted terimine-centered 
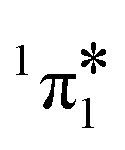
 ← ^1^π bands in the 350–400 nm range (orange and black traces Fig. 10a, right), which are obviously lacking for [**dig**Y(hfac)_3_] (green trace in Fig. 10a, right). Therefore, selective excitation into the terimine core in [**L2**Y(hfac)_3_] can be achieved upon irradiation at *λ*_exc_ = 380 nm while avoiding contributions from both the pyrene and the hfac^−^ chromophores to the overall photoexcitation process.

**Fig. 10:**
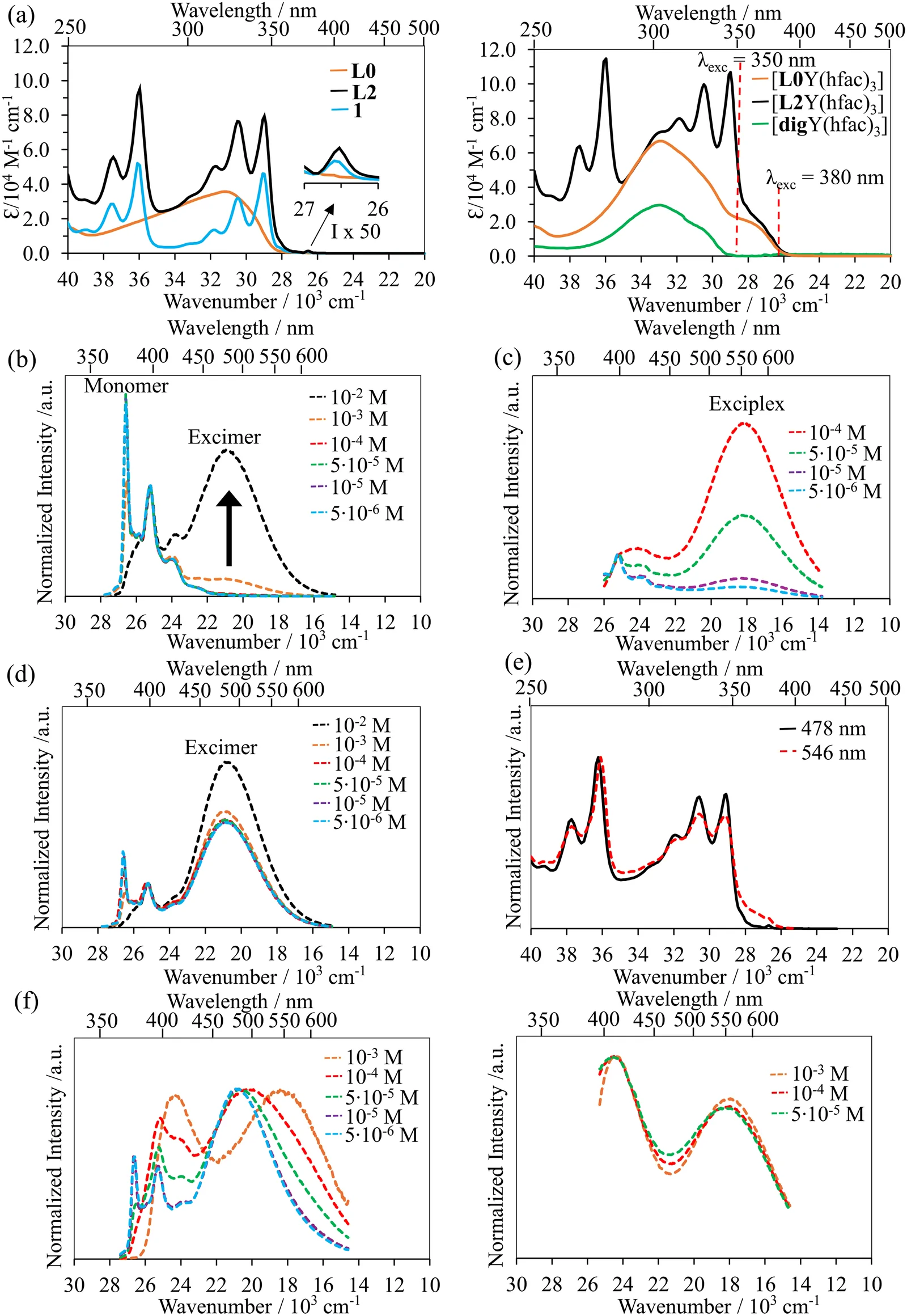
(a) UV-vis absorption spectra of 1, **L0, L2** (left) and [**L*k***Y(hfac)_3_] (**L*k*** = **L0**, **L2** and **dig** (right) in dichloromethane. Emission spectra of (b) 1,^34^ (*λ*_exc_ = 345 nm), (c) [**L1**Y(hfac)_3_] (*λ*_exc_ = 375 nm),^34^ (d) **L2** (*λ*_exc_ = 345 nm) and (f) [**L2**Y(hfac)_3_] at *λ*_exc_ = 350 nm (left) and *λ*_exc_ = 380 nm (right) in dichloromethane. (e) Excitation spectra of [**L2**Y(hfac)_3_] monitored *λ*_em_ = 478 nm (black trace) and *λ*_em_ = 546 nm (red trace) in dichloromethane.

Alternatively, recording fluorescence spectra upon excitation at *λ*_exc_ = 350 nm involves the excited states of both the pyrene and the terimine cores, but with a still negligible contribution of the hfac^−^ co-ligand due to its faint absorption at this excitation wavelength. The room-temperature fluorescence spectra of the mono-pyrene model 1 displays (i) only the 370–390 nm monomer emission band at diluted solutions (10^−6^–10^−4^ M) and (ii) an additional structureless broad excimer band at 477 nm at higher concentrations approaching 10^−2^ M (Fig. 10b).^34^ Upon photoexcitation at *λ*_exc_ = 345 nm, the bis-pyrene **L2** exhibits both the monomer emission at 376–398 nm and the excimer emission centered at 479 nm with an excimer/monomer intensity ratio which is concentration-independent in the range of 10^−4^–5 × 10^−6^ M (blue, violet, green and red traces in Fig. 10d and A4-1b). This confirms the intramolecular folding of **L2** found in the ground state solution and the static nature of the intramolecular excimer formation between the two nearby-attached pyrene units. The gradual growth of the excimer band for **L2** at higher concentration (orange and black traces in Fig. 10d) is tentatively attributed to the presence of additional dynamic intermolecular excimers in line with those observed in 1 (orange and black traces in Fig. 10b).

We then investigated the excited state aggregation properties of [**L2**Y(hfac)_3_] by monitoring its concentration-dependent emission profiles upon photoexcitation at *λ*_exc_ = 350 nm (Fig. 10f). At diluted concentration (10^−5^–5 × 10^−6^ M), [**L2**Y(hfac)_3_] displays both the pyrene monomer emission in the range of 376–396 nm, which fully masks the emission of the terimine core in line with our previous study,^34^ and the pyrene excimer emission at 478 nm, together with the observed concentration-independent behavior of the excimer/monomer intensity ratio (blue and violet traces in Fig. 10f, left, and A4-1a), indicating the intramolecular nature of the dynamic excimer generated from the two pyrene units attached to the benzimidazole side arms in [**L2**Y(hfac)_3_] in the excited state. The dynamic excimer emission band at 478 nm (red trace in Fig. 10f, left) is then gradually red-shifted toward 546 nm (red and orange traces in Fig. 10f, left) with increasing concentrations of [**L2**Y(hfac)_3_] from 10^−5^–10^−3^ M due to the additional contribution of intermolecular hetero-excimer (exciplex) emission produced by communication between the pyrene and the terimine units in the excited state. One notices that the relative decrease of the monomeric pyrene emission (376–396 nm) at high concentration results in the detection of a ‘new’ short wavelength band at around 400 nm assigned to the monomeric emission of the terimine core (red and orange traces in Fig. 10f left).^34^ Selective excitation of the 
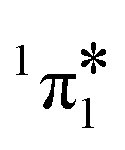
 ← ^1^π transition of the terimine unit at *λ*_exc_ = 380 nm in [**L2**Y(hfac)_3_] yields predominantly exciplex emission at 546 nm (Fig. 10f, right), thus confirming the intermolecular photophysical interaction between the excited state terimine donor and the ground state pyrene acceptor in agreement with the light-driven dimerization found in [**L1**Y(hfac)_3_] (Fig. 10c).^34^ The excitation spectrum of [**L2**Y(hfac)_3_] (Fig. 10e) monitored at (i) *λ*_em_ = 546 nm is found to be near-identical to its absorption profile, reinforcing that the 546 nm dimeric emission is formed by the pyrene and the terimine units, while that monitored at (ii) *λ*_em_ = 478 nm mimics the absorption spectrum of the pyrene-containing 1, thus confirming that the 478 nm excimeric band is produced from the two pyrene units without contribution of the terimine core. Based on these results, it is worth noting that the nature of the dimeric emission in [**L2**Y(hfac)_3_] can be tuned from the intramolecular homo-excimer to the intermolecular hetero-excimer by varying the concentration and selecting the excitation wavelength.

The sensitivity of the intramolecular static excimer in **L2** and the intramolecular dynamic excimer in [**L2**Y(hfac)_3_] towards nitrobenzene (NB), tested as a quencher, is then investigated by fluorescence titrations under similar experimental conditions (Fig. 11). The addition of NB (1.01 × 10^−2^ M) to a highly diluted concentration (6.8 × 10^−6^ M) of **L2** (to prevent the formation of the dynamic excited dimer) leads to stepwise reduced intensities of the static excimer resulting from the competitive π–π interactions involving the electron-poor aromatic NB and the neutral pyrene in **L2** (Fig. 11a, left). The fluorescence quenching efficiency by NB is determined from the Stern–Volmer eqn (16),^41^ in which *I*_0_ and *I* correspond to the excimer/exciplex emission intensities in the absence and presence of NB quencher, respectively, [Q] stands for the concentration of NB quencher, and *K*_SV_ is the Stern–Volmer quenching constant:16
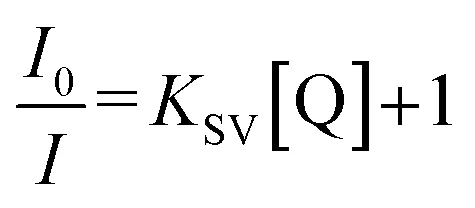
The titration of **L2** with NB exhibits a linear Stern–Volmer plot shown in Fig. 11a (right), from which the quenching constant *K*^**L2**^_SV_ = 1307(54) (Fig. 11a, right) can be estimated.

**Fig. 11:**
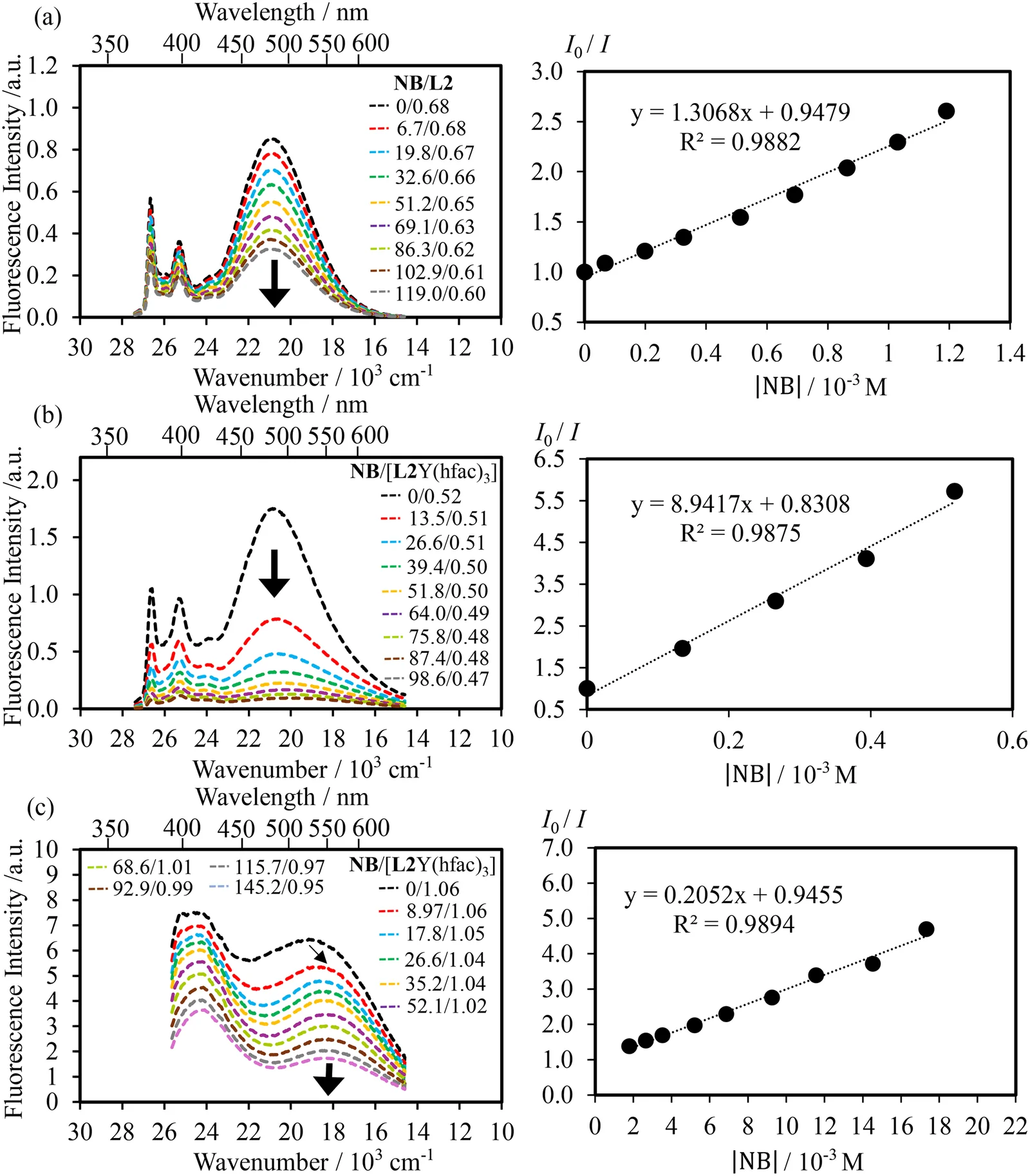
Fluorescence titration (left) and Stern–Volmer plot (right) of (a) **L2** (6.8 × 10^−6^ M) at *λ*_exc_ = 350 nm, (b) [**L2**Y(hfac)_3_] (5.2 × 10^−6^ M) at *λ*_exc_ = 350 nm, and (c) [**L2**Y(hfac)_3_] (1.06 × 10^−4^ M) at *λ*_exc_ = 380 nm with nitrobenzene (NB).

Interestingly, the addition of NB (1.01 × 10^−2^ M) to the diluted solution (5.2 × 10^−6^ M) of complex [**L2**Y(hfac)_3_] (to avoid the formation of the intermolecular exciplex) similarly leads to a decrease of the intensity of the intramolecular dynamic excimer due to the competitive π–π interactions between the electron poor aromatic NB and the neutral pyrene in [**L2**Y(hfac)_3_] (Fig. 11b, left). The associated linear Stern–Volmer plot (Fig. 11b, right) estimates a quenching constant of *K*^**L2**Y^_SV_ = 8942(581), a value seven times larger than that of the free ligand **L2**. This behavior could be explained by the ground-state π-stacked arrangement found in the folded-pyrene **L2** conformer, making the static excimer stable enough so that it cannot be easily disrupted by the external aromatic NB guest. Therefore, the ground-state intramolecular π-stacked organization in the folded-pyrene **L2** ensures the stability of the static excimer for its application in organic optoelectronic materials, while the open-pyrene [**L2**Y(hfac)_3_] can act as a candidate for chemical sensor due to its enhanced sensitivity to external perturbations.

The sensitivity of the dimeric emission in [**L2**Y(hfac)_3_] to the addition of NB was also studied at 10^−4^ M (Fig. 11c) upon photoexcitation at 380 nm into the terimine units to minimize the contribution of the pyrene–pyrene excimer to the pyrene–terimine exciplex formation. Upon addition of small aliquots of a highly concentrated solution (1.29 × 10^−1^ M) of NB (to prevent the emission shift due to the dilution) to the sample of [**L2**Y(hfac)_3_] at 1.06 × 10^−4^ M concentration (to promote the dynamic exciplex formation), the intensity of the dimeric emission at 525 nm is decreased accompanied by a bathochromic shift of the dimeric emission band to 538 nm (Fig. 11c, left). This phenomenon reveals that NB quenches preferentially the pyrene–pyrene excimer due to the competitive π–π interactions between the electron-poor aromatic NB and the neutral pyrene in [**L2**Y(hfac)_3_]. The intensity of the excimer–exciplex mixture at 538 nm (red trace in Fig. 11c, left) decreases stepwise upon addition of NB, resulting in predominantly exciplex at 544 nm (blue trace in Fig. 11c, left) which is subsequently quenched by NB with a small quenching constant *K*^**L**2Y,exciplex^_SV_ = 205(8) (Fig. 11c, right). This implies a weak competitive interaction between the electron-poor NB guest and the neutral pyrene in the presence of the electron poor terimine unit in the [**L2**Y(hfac)_3_] host.

## Conclusion

This work highlights the role of metal for tuning the nature of π-stacked organization in the bichromophoric pyrene–terimine ligand **L2** and its yttrium complex [**L2**Y(hfac)_3_]. The planar aromatic pyrene unit is involved in the intra/intermolecular π-stacked arrangements, while the nitrogen-donor terimine core is utilized as the metal-binding center for controlling the static/dynamic nature of the excited dimeric emission. In the ground state, the free ligand **L2** displays an intramolecular enthalpically favored π-stacked arrangement between the two tethered pyrene units with *K*^**L2**^_Fold_ = 22.1 at 298 K determined by ^1^H NMR titration. The ground state intramolecular π-stacked organization in the folded-pyrene **L2** leads to the decreased sensitivity of its static excimer to the external aromatic nitrobenzene quencher, ensuring the stability of the static excimer for its application in organic optoelectronic materials. The binding of **L2** to the yttrium ion leads to the dissociation of the intramolecular π-stacked interactions, together with the detection of novel weak intermolecular entropically governed ground state pyrene–pyrene (*K*^**L2**Y^_P–P_ = 3.02, 298 K) and pyrene–terimine (*K*^**L2**Y^_P–T_ = 1.01, 298 K) dimerization in the resulting complex [**L2**Y(hfac)_3_]. This is fully consistent with the observation found in its crystal structure and results from the *trans*–*trans* to *cis*–*cis* conformational change required for the host–guest interaction as well as the intramolecular polarization induced upon nitrogen-metal binding reaction. Interestingly, the intermolecular pyrene–pyrene homo-packing can be removed upon photoexcitation of the terimine core in [**L2**Y(hfac)_3_] complex, resulting in the pure hetero-dimerization between the pyrene and the terimine units in the excited state. This paves the way for selectively producing hetero-coupling in the presence of two different chromophores in photochemical reactions in organic synthesis, a process that is difficult to achieve in the absence of metal-templated strategy. The sensitivity of this dynamic excited dimer in [**L2**Y(hfac)_3_] complex to the electron poor nitrobenzene guest is higher than that of the static excimer of the free ligand **L2**, visualized by a higher Stern–Volmer quenching constant (*K*^**L2**Y^_SV_ = 8942(581)) for [**L2**Y(hfac)_3_] in comparison to that determined for the free ligand **L2** (*K*^**L2**^_SV_ = 1307(54)), indicating that [**L2**Y(hfac)_3_] can act as a candidate for chemo/biosensor due to its enhanced sensitivity to the external guest.

## Experimental section

### General

Dichloromethane was distilled over calcium hydride. All other chemicals and reagents were purchased from Alfa Aesar, FluroChem, and Sigma-Aldrich and used as received. ^1^H and ^13^C NMR spectra were recorded at 298 K on a Bruker Avance 400 MHz spectrometer equipped with BCU temperature control for variable temperature measurements. Electrospray (ESI-MS) mass spectra were recorded on an Applied Biosystems API 150EX LC/MS System equipped with a Turbo Ionspray source. Elemental analyses were performed by K. L. Paglia from the Microchemical Laboratory of the University of Geneva. Electronic spectra in the UV-vis region were recorded at 293 K from solutions in CH_2_Cl_2_ using a 1 cm path-length cuvette with a Perkin-Elmer Lambda 1050 instrument. The emission spectra were recorded in a 1 cm path-length cuvette using a Fluorolog (Horiba Jobin Yvon) instrument equipped with an iHR320 imaging spectrometer, a 450 W xenon lamp illuminator (FL-1039A/40A) and a water-cooled photomultiplier tube (PMT Hamamatsu R928P) with a wavelength increment of 1 nm and an integration time of 1 s*.* The emission spectra were corrected for the wavelength-dependent sensitivity of the PMT. The mathematical analyses were performed by using Origin 2017 (OriginLab Corporation) and Excel® (Microsoft) software. Single-crystal X-ray crystallography: crystals were mounted on Hampton cryoloops with protection oil. X-ray data collections were performed with a XtaLAB Synergy-S diffractometer equipped with a hybrid pixel array “hypix arc 150” detector (Cu[Kα] radiation) and under a N_2_ cooling system. The structures were solved by using dual-space methods (ShelXT).^42^ Full-matrix least-squares refinements on *F*^2^ were performed with SHELXL^42,43^ and all other calculations were performed with OLEX2 (ref. 44) and ORTEP^45^ programs. The interplanar distances and angles between the aromatic planes were determined from the single-crystal X-ray structures using the geometrical analysis routines implemented in OlEX2.^44^ The degree of aromatic overlap was estimated by projecting one aromatic unit onto the mean plane of the other and then determining the percentage of overlapping carbon atoms.

### Syntheses of ligand **L2** and its lanthanide complexes

#### L2

A mixture of **2** (0.5 g, 1.61 mmol) and NaH 60% (0.20 g, 5.0 mmol) in dry DMF (10 mL) was stirred at rt for 30 min. 1 (1.63 g, 4.83 mmol) was added. The mixture was stirred at 50 °C for 24 h and then poured into water (20 mL). The mixture was extracted with CH_2_Cl_2_ (3 × 50 mL), dried over Na_2_SO_4_ and evaporated to dryness. The residue was purified by column chromatography (SiO_2_, CH_2_Cl_2_/MeOH 100/0 to 100/5) to afford **L2** (0.81 g, 61%) as a white solid. ^1^H NMR (400 MHz, CD_2_Cl_2_): 8.21 (d, *J* = 7.9 Hz, 2H), 8.17 (d, *J* = 7.6 Hz, 2H), 8.13 (d, *J* = 7.7 Hz, 2H), 8.05–7.90 (m, 10H), 7.90–7.84 (m, 3H), 7.81 (dt, *J* = 8.0, 1.0 Hz, 2H), 7.50 (d, *J* = 7.8 Hz, 2H), 7.35–7.29 (m, 2H), 7.28–7.23 (m, 2H), 7.23–7.19 (m, 2H), 4.56 (t, *J* = 7.4 Hz, 4H), 3.07 (t, *J* = 7.5 Hz, 4H), 1.82–1.72 (m, 4H), 1.63–1.51 (m, 4H). ^13^C NMR (101 MHz, CD_2_Cl_2_): 150.1, 150.0, 142.9, 137.8, 136.4, 136.0, 131.3, 130.8, 129.7, 128.4, 127.4, 127.1, 127.1, 126.5, 125.8, 125.4, 124.8, 124.8, 124.7, 124.6, 123.2, 123.0, 122.4, 120.1, 110.4, 44.7, 32.7, 29.8, 28.34. ESI-MS calculated for [C_59_H_45_N_5_ + H]^+^ ([M + H]^+^): *m*/*z* 824.4; found: 824.8. Elemental analysis calculated for C_59_H_45_N_5_ (**L2**) (%): C 86.00, H 5.50, N 8.50; found (%): C 85.69, H 5.38, N 8.35.

### General procedure for the synthesis of [**L2**Ln(hfac)_3_] (Ln = Y and Eu)

A mixture of **L2** and [**dig**Ln(hfac)_3_] (1.2 eq.) in CH_2_Cl_2_ was stirred at rt for 30 min and then evaporated to dryness. The residue was purified by precipitation in pentane to afford [**L2**Ln(hfac)_3_] complexes.

#### [**L2**Eu(hfac)_3_]

Yield: 58%. ^1^H NMR (400 MHz, CD_2_Cl_2_): 21.99 (s, 2H), 11.07 (s, 2H), 9.92–9.83 (m, 2H), 8.81 (d, *J* = 8.3 Hz, 2H), 8.31 (d, *J* = 9.2 Hz, 2H), 8.22 (dd, *J* = 7.5, 1.2 Hz, 2H), 8.14–8.00 (m, 10H), 7.97 (d, *J* = 7.7 Hz, 2H), 7.86 (d, *J* = 9.2 Hz, 2H), 6.38 (t, *J* = 7.7 Hz, 1H), 6.11 (d, *J* = 7.8 Hz, 2H), 5.76 (t, *J* = 8.1 Hz, 4H), 3.74 (t, *J* = 7.5 Hz, 4H), 3.26–3.12 (m, 4H), 2.71 (t, *J* = 7.8 Hz, 4H). ^13^C NMR (101 MHz, CD_2_Cl_2_): 156.2, 152.9, 145.6, 143.1, 135.7, 132.1, 131.4, 131.2, 130.8, 130.0, 128.7, 128.3, 127.4, 127.4, 127.4, 126.8, 126.0, 125.1, 124.9, 124.8, 123.1, 118.2, 111.5, 99.1, 59.9, 58.8, 55.9, 45.58, 32.9, 32.0, 29.2. ESI-MS calculated for [C_74_H_48_N_5_F_18_O_6_Eu–hfac]^+^ ([M–hfac]^+^): *m*/*z* 1390.3; found: 1390.0. Elemental analysis calculated for C_74_H_48_N_5_F_18_O_6_Eu ([**L2**Eu(hfac)_3_]) (%): C 55.65, H 3.03, N 4.38; found (%): C 55.47, H 3.04, N 4.15.

#### [**L2**Y(hfac)_3_]

Yield: 54%. ^1^H NMR (400 MHz, CD_2_Cl_2_): 8.33 (d, *J* = 9.3 Hz, 2H), 8.23–8.05 (m, 14H), 8.02 (t, *J* = 7.6 Hz, 2H), 7.92 (d, *J* = 7.8 Hz, 2H), 7.56 (d, *J* = 8.0 Hz, 2H), 7.47 (d, *J* = 8.1 Hz, 2H), 7.45–7.39 (m, 3H), 7.37–7.31 (m, 2H), 5.89 (s, 3H), 4.48 (d, *J* = 7.8 Hz, 4H), 3.59–3.46 (m, 4H), 2.26–2.10 (m, 8H). ^13^C NMR (101 MHz, CD_2_Cl_2_): 175.5, 175.2, 148.6, 147.2, 140.1, 139.9, 135.8, 135.5, 131.4, 130.8, 130.0, 128.6, 127.5, 127.4, 126.8, 126.0, 125.2, 125.1, 124.9, 124.9, 123.8, 123.1, 122.3, 119.1, 116.2, 109.8, 89.6, 45.9, 32.6, 29.4, 28.7. ESI-MS calculated for [C_74_H_48_N_5_F_18_O_6_Y–hfac]^+^ ([M–hfac]^+^): *m*/*z* 1326.3; found: 1326.9. Elemental analysis calculated for C_74_H_48_N_5_F_18_O_6_Y ([**L2**Y(hfac)_3_]) (%): C 57.94, H 3.15, N 4.57; found (%): C 57.81, H 3.13, N 4.57.

## Author contributions

G. L. H. designed the synthetic strategies, performed all the experiments and wrote the first draft of the contribution, L. G. solved the crystal structures, and C. P. managed the whole project, obtained funding and finalized the version to be published.

## Conflicts of interest

The authors declared no conflict of interest.

## Supplementary Material

CE-028-D6CE00611F-s001

CE-028-D6CE00611F-s002

## Data Availability

Supplementary information (SI): experimental section, NMR spectra, crystal structures, thermodynamic data, and photophysical data for the synthesized compounds. See DOI: https://doi.org/10.1039/d6ce00611f. CCDC 2561838–2561840 (**L2**, [**L2**Eu(hfac)_3_]·0.265CH_2_Cl_2_, [**L2**Y(hfac)_3_]·0.233CH_2_Cl_2_) contain the supplementary crystallographic data for this paper.^46*a*–*c*^
