# Supplementary material for: Generating, visualizing and tuning π–π interactions in metal-induced supramolecular assembly
Source: CrystEngComm. 2026 Sep 4;28(41):5808–22. doi: 10.1039/d6ce00611f (PMC13622079; doi:10.1039/d6ce00611f)
Supplement: Supplementary file 1 [file CE-028-D6CE00611F-s001.pdf]

Supplementary Information (SI) for CrystEngComm.
This journal is © The Royal Society of Chemistry 2026

Generating, visualizing and tuning π-π interactions in metal-induced supramolecular assembly

Giau Le-Hoang,[*a] Laure Guénée,[b] and Claude Piguet[*a]

[a] Department of Inorganic and Analytical Chemistry, University of Geneva, 30 quai E. Ansermet, CH-1211 Geneva 4, Switzerland.

Emails: Hoang.Le@unige.ch, Claude.Piguet@unige.ch

[b] *Laboratory of Crystallography, University of Geneva, 24 quai E. Ansermet, CH-1211 Geneva 4, Switzerland.*

Supporting Information

(39 pages)

Spectroscopic and analytical measurements

^{1}H and ^{13}C NMR spectra were recorded at 298 K on a Bruker Avance 400 MHz spectrometer equipped with BCU temperature control for variable temperature measurements. Electrospray (ESI-MS) mass spectra were recorded on an Applied Biosystems API 150EX LC/MS System equipped with a Turbo Ionspray source. Elemental analyses were performed by K. L. Paglia from the Microchemical Laboratory of the University of Geneva. Electronic spectra in the UV-Vis region were recorded at 293 K from solutions in CH_2Cl_2 with a Perkin-Elmer Lambda 1050. The emission spectra were recorded using a Fluorolog (Horiba Jobin-Yvon) instrument equipped with an iHR320 imaging spectrometer, a 450 W xenon lamp illuminator (FL-1039A/40A) and a Peltier-cooled photomultiplier tube (PMT Hamamatsu R928P). The emission spectra were corrected for the wavelength-dependent sensitivity of the PMT. The standard xenon lamp of the Horiba Scientific Fluorolog 3 spectrofluorometer has been used for UV excitation of the samples to record the pertinent emission spectra, where the appropriate longpass filters (Thorlabs) have been placed after the sample to remove the second-order Rayleigh scattering of the xenon lamp. The emission spectra were corrected for the instrumental response function. The mathematical analyses were performed by using Origin 2017 (OriginLab Corporation) and Excel® (Microsoft) software.

Appendix 1: Synthesis of target ligand L2.

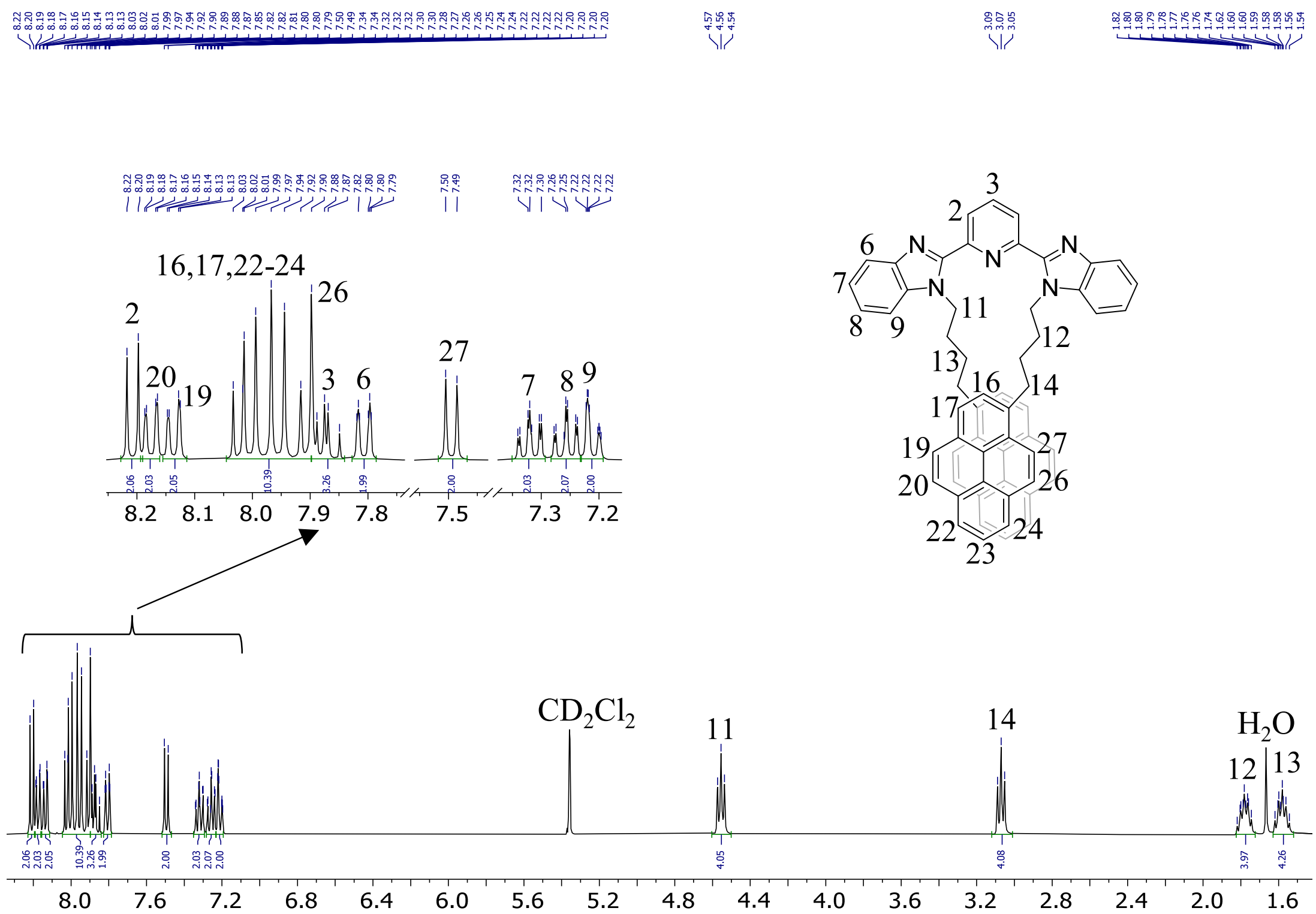

Figure A1-1. ^{1}H NMR spectrum of ligand **L2** in CD_2Cl_2.

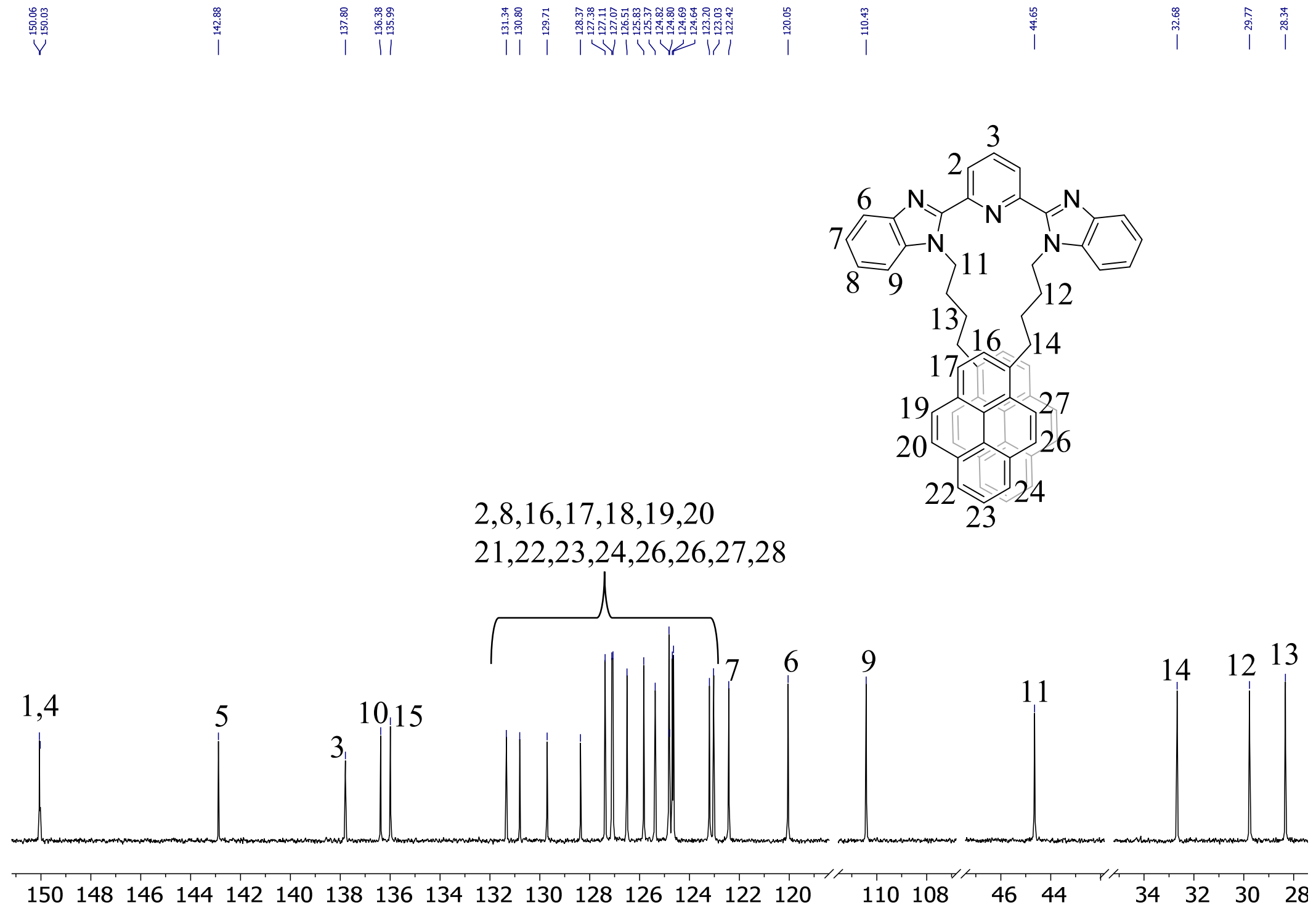

Figure A1-2. ^{13}C NMR spectrum of ligand **L2** in CD_2Cl_2.

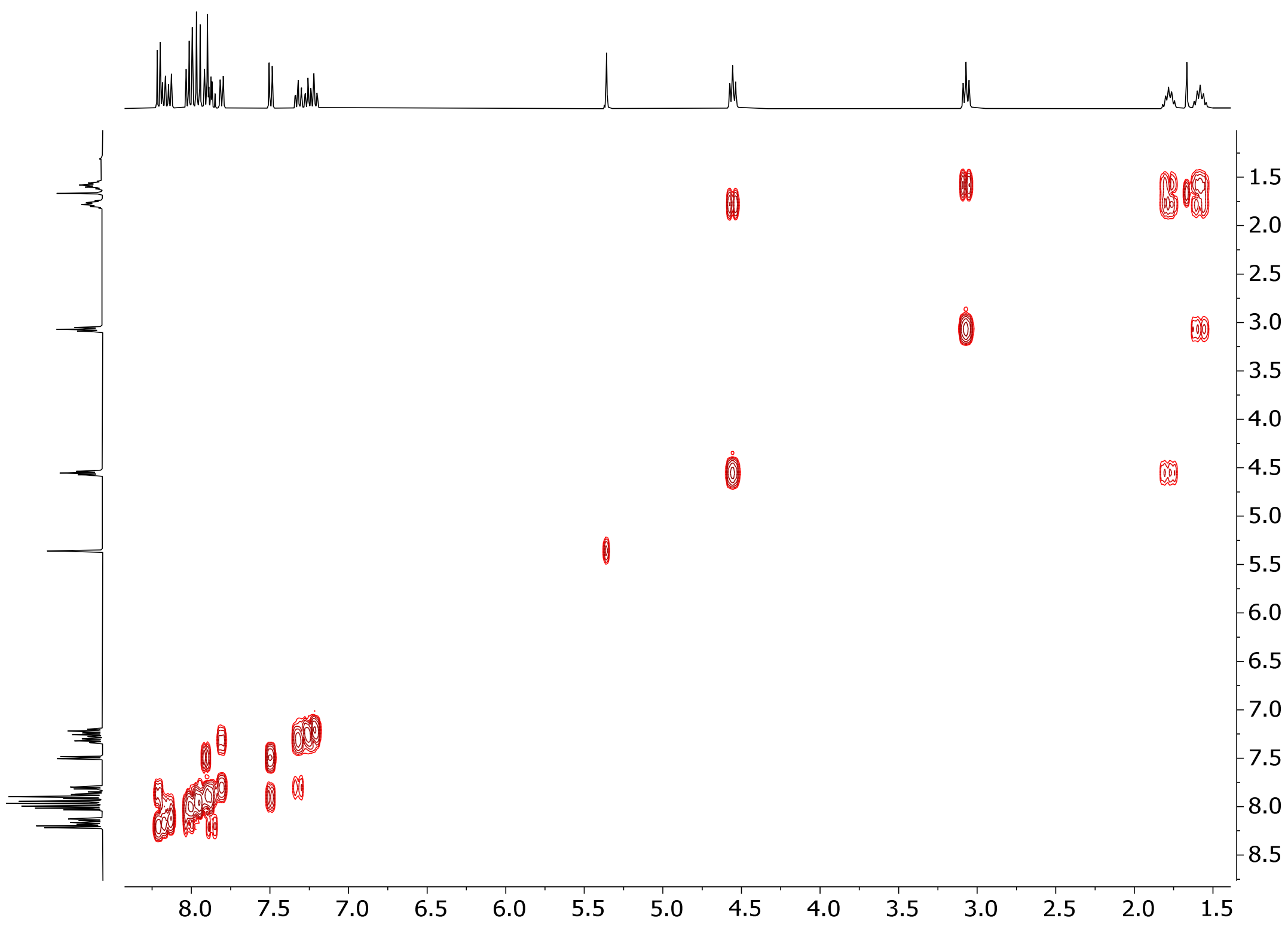

Figure A1-3. COSY spectrum of ligand **L2** in CD_2Cl_2.

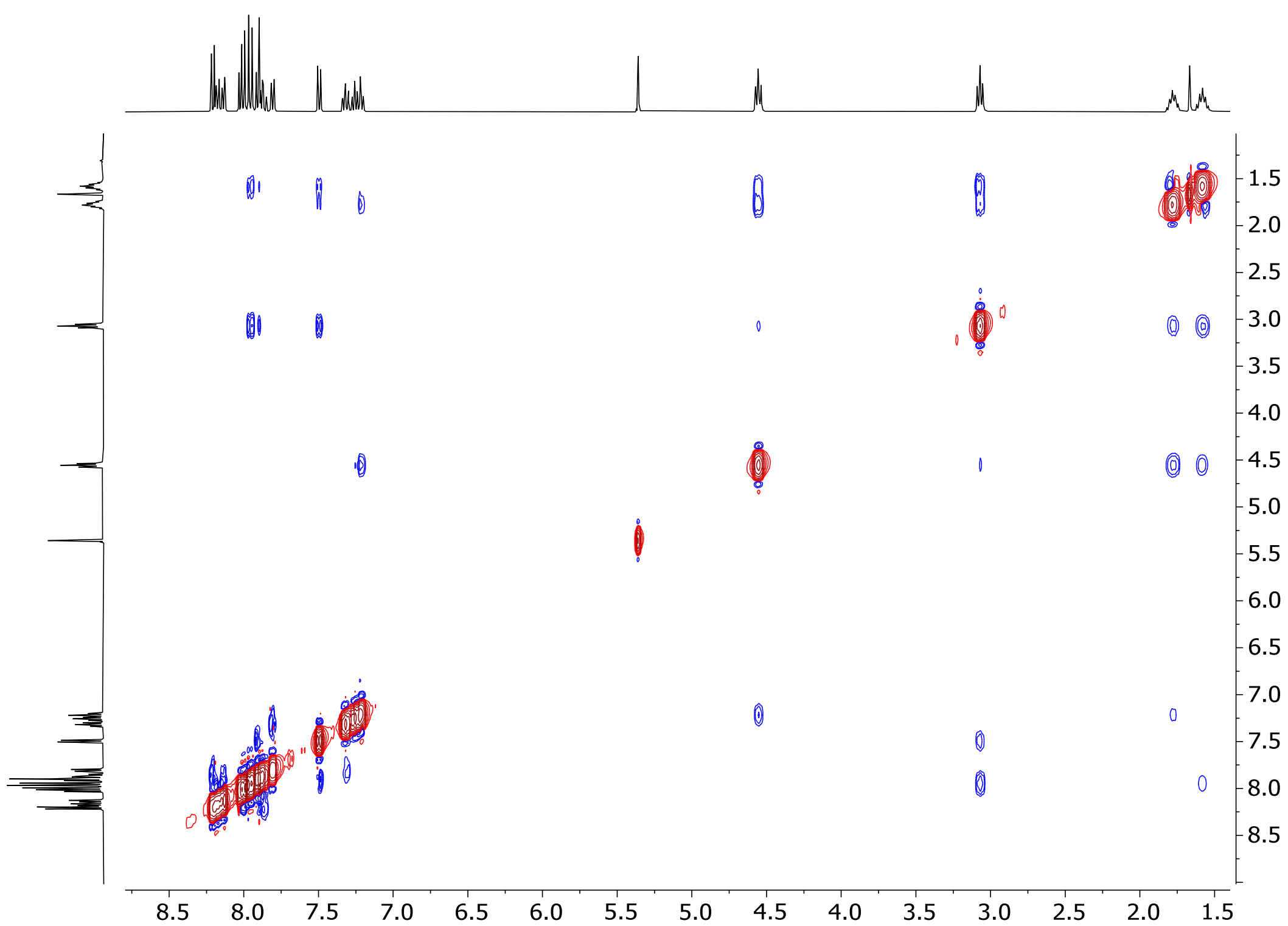

Figure A1-4. NOESY spectrum of ligand **L2** in CD_2Cl_2.

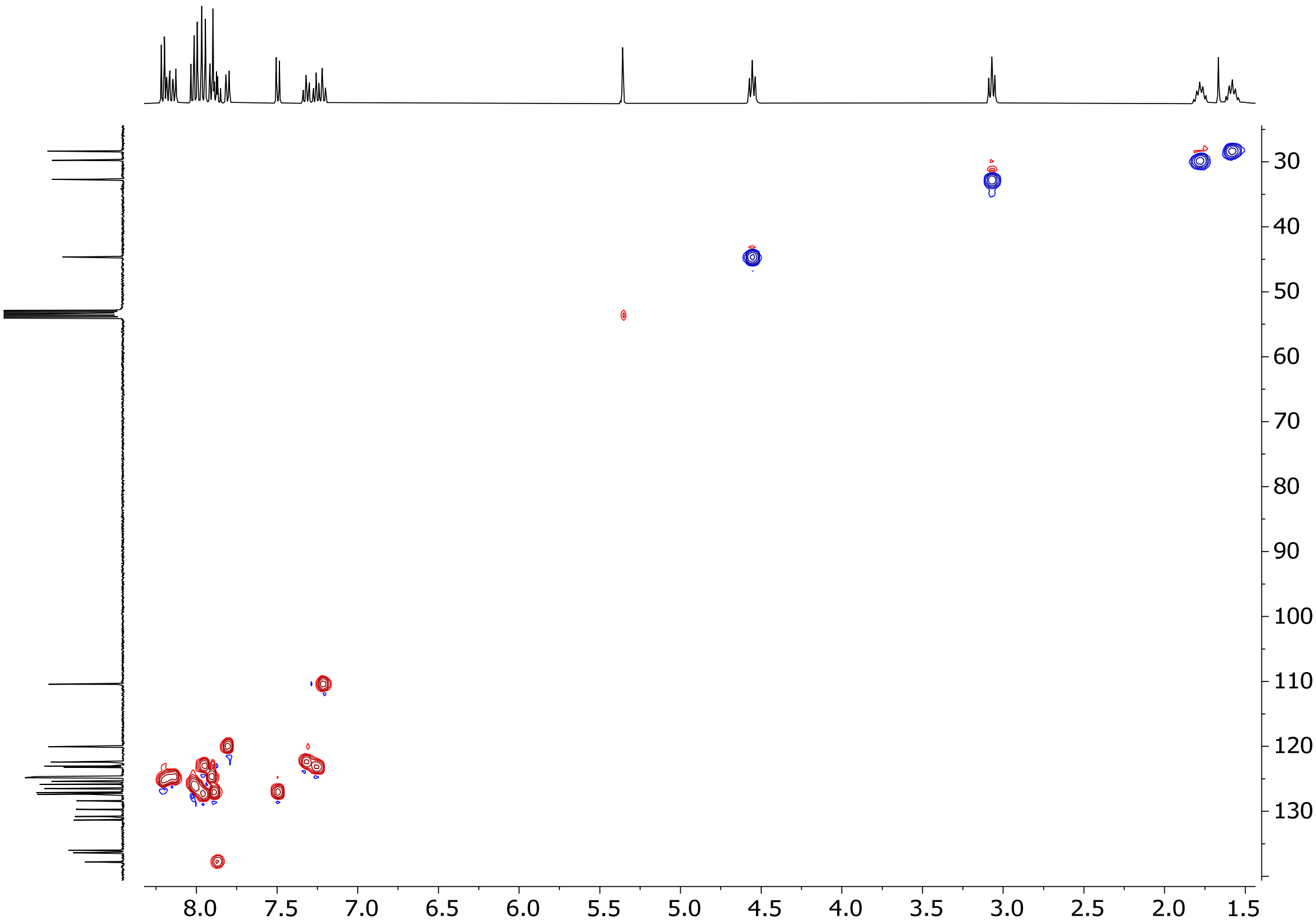

Figure A1-5. HSQC spectrum of ligand **L2** in CD_2Cl_2.

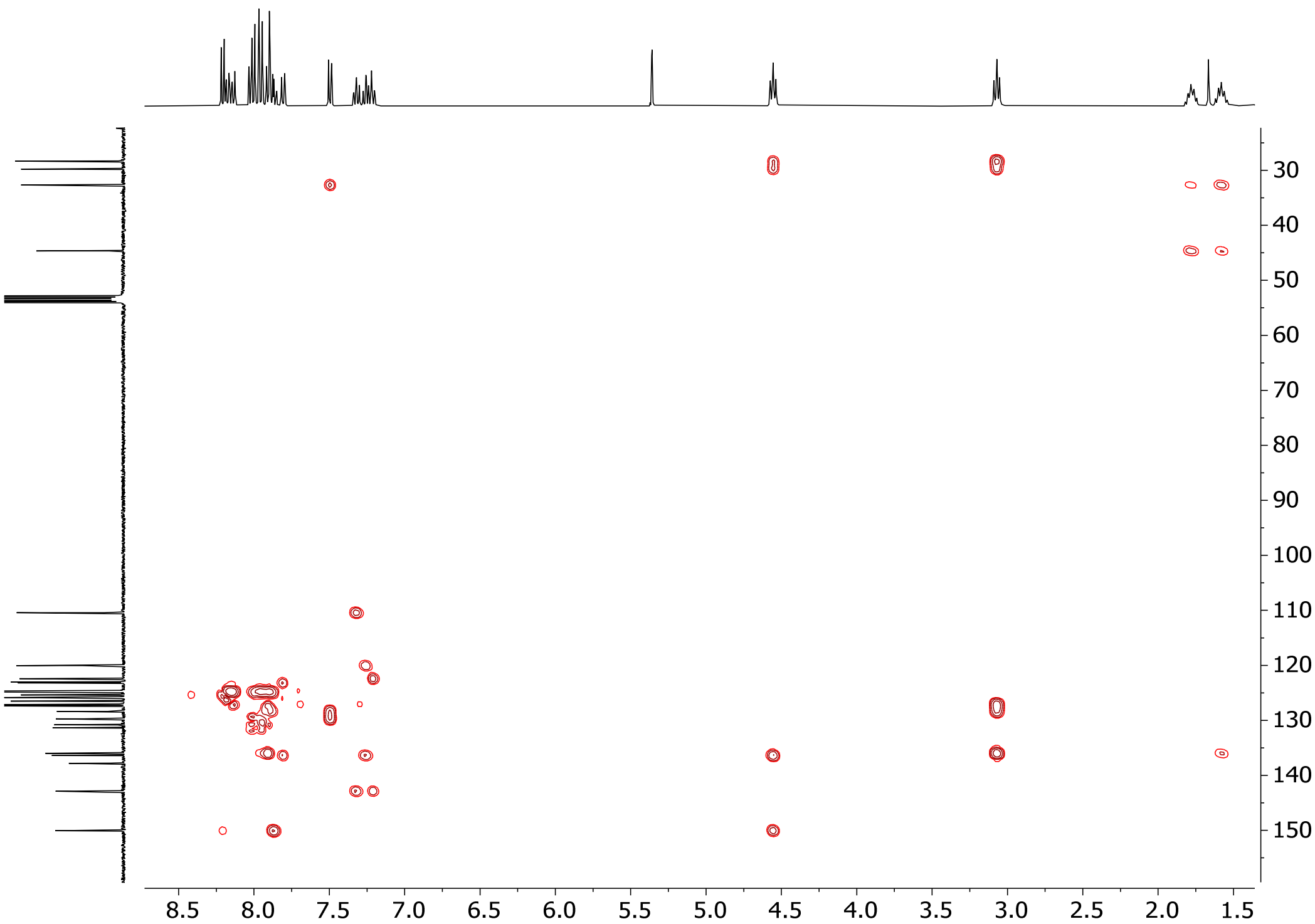

Figure A1-6. HMBC spectrum of ligand **L2** in CD_2Cl_2.

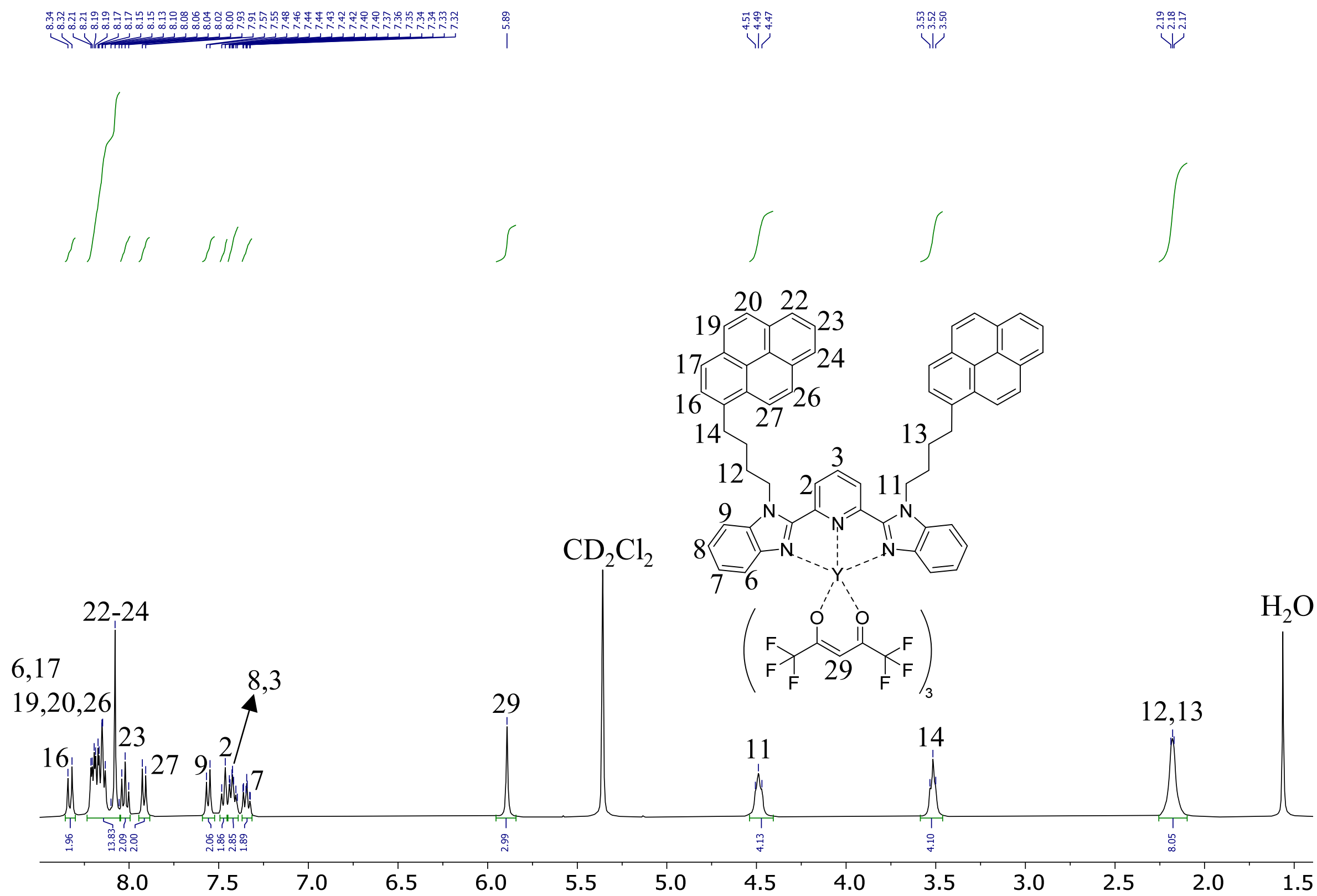

Figure A1-7. ^{1}H NMR spectrum of [**L2**Y(hfac)$_3$] in CD_2Cl_2.

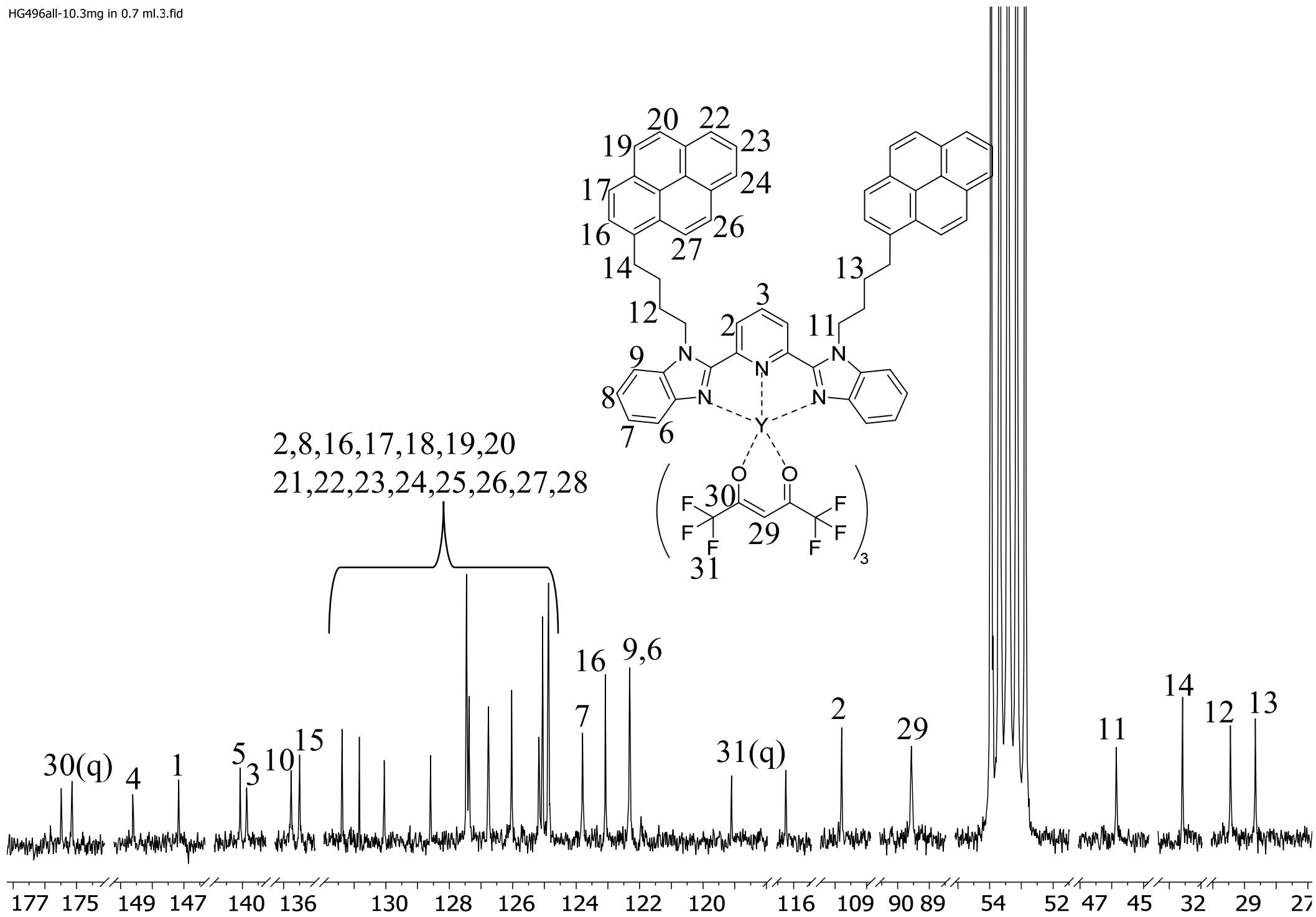

Figure A1-8 ^{13}C NMR spectrum of [**L2**Y(hfac)$_3$] in CD_2Cl_2.

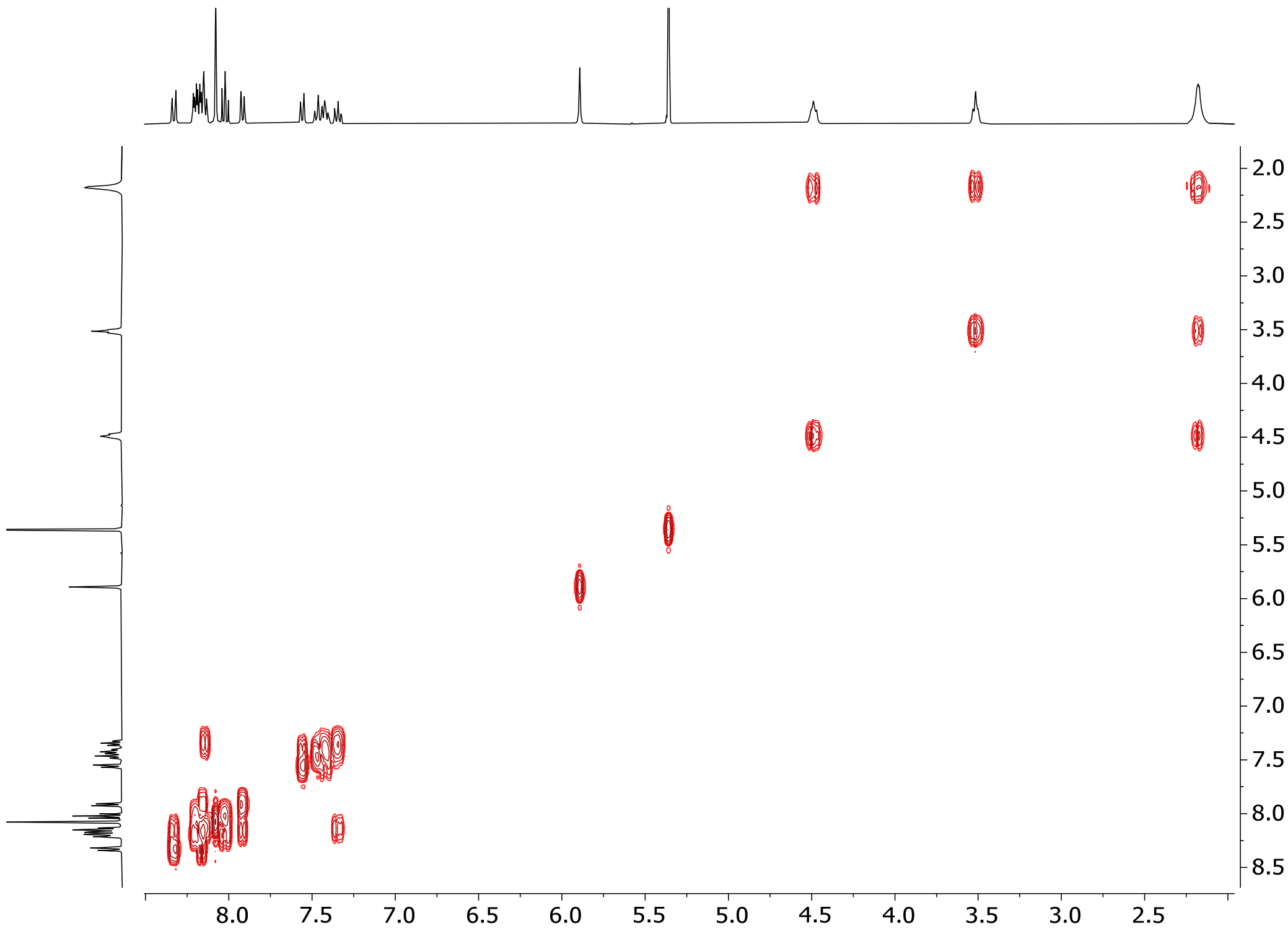

Figure A1-9. COSY spectrum of [**L2**Y(hfac)$_3$] in CD_2Cl_2.

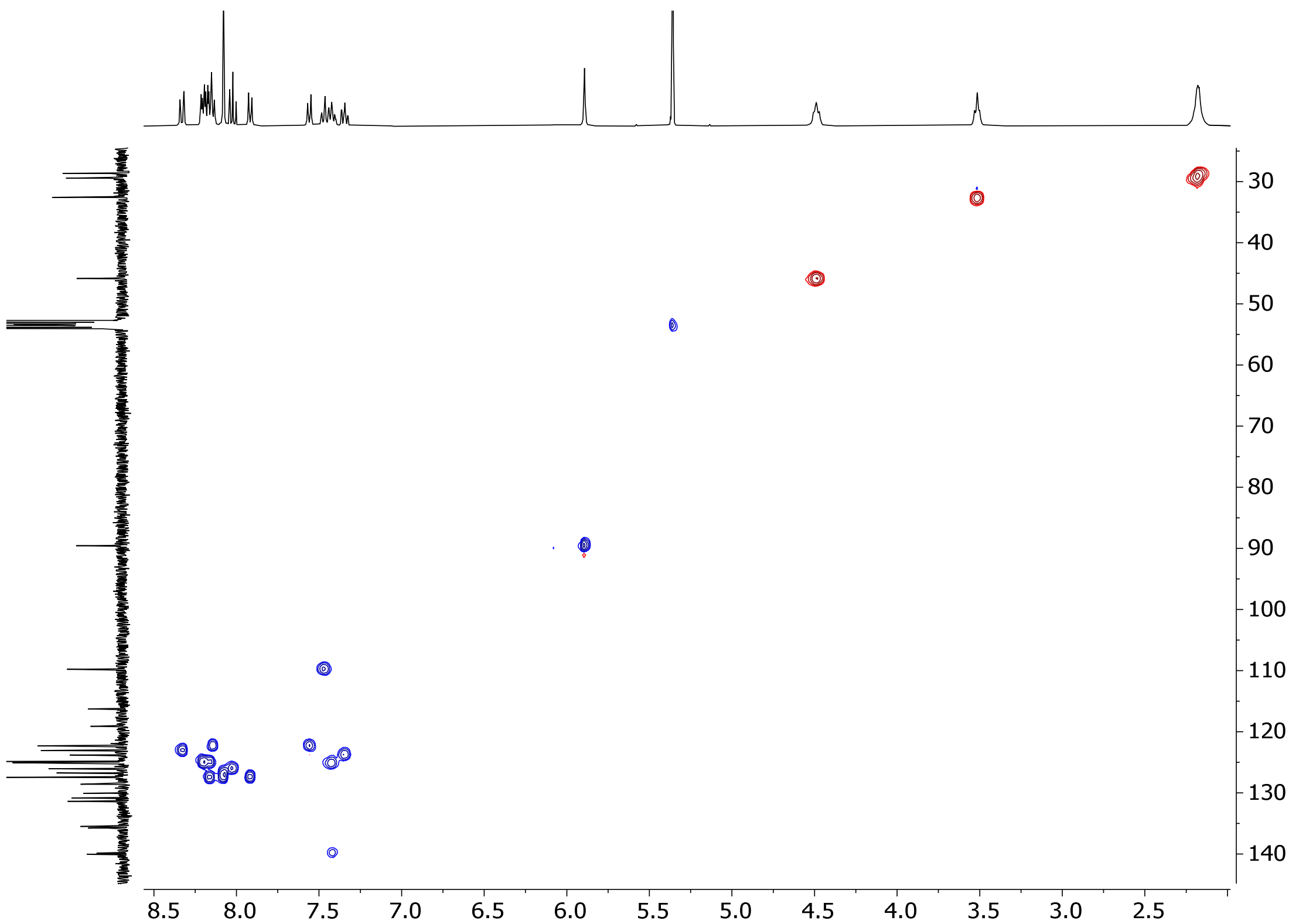

Figure A1-10. HSQC spectrum of [**L2**Y(hfac)$_3$] in CD_2Cl_2.

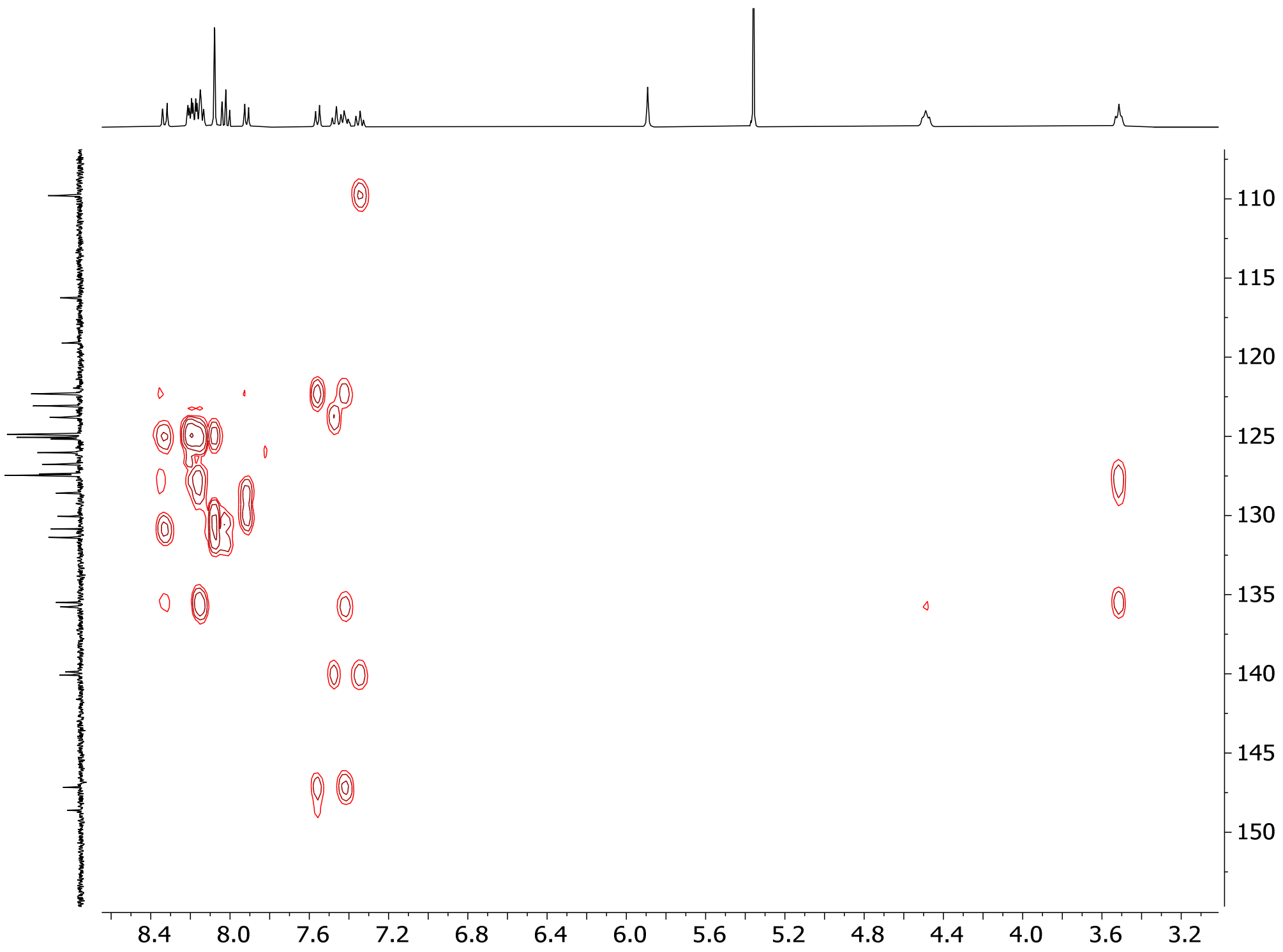

Figure A1-11. HMBC spectrum of [**L2**Y(hfac)$_3$] in CD_2Cl_2.

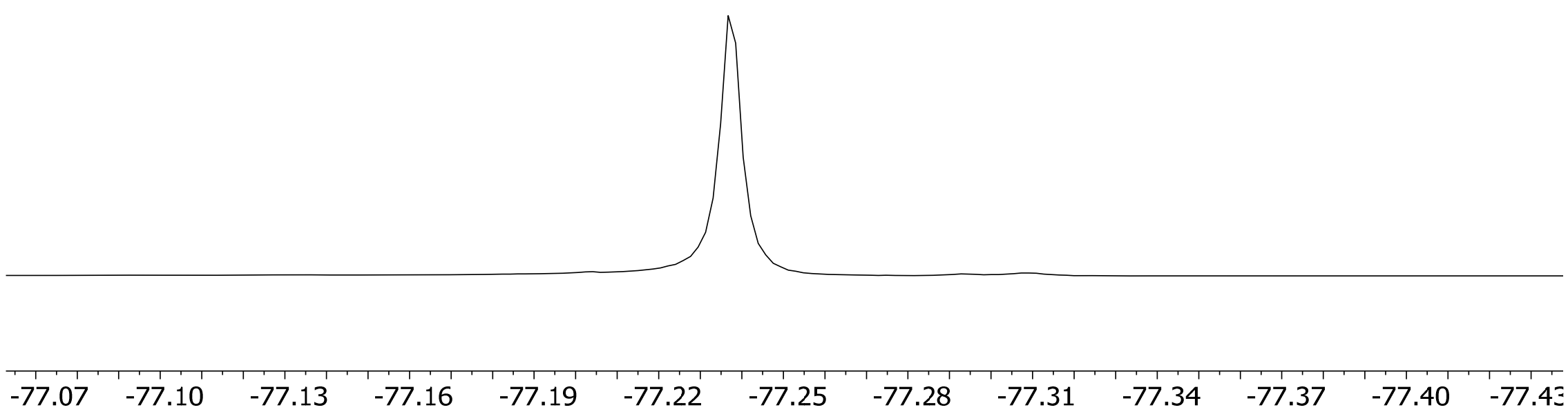

Figure A1-12. ^{19}F NMR spectrum of [**L2**Y(hfac)$_3$] in CD_2Cl_2.

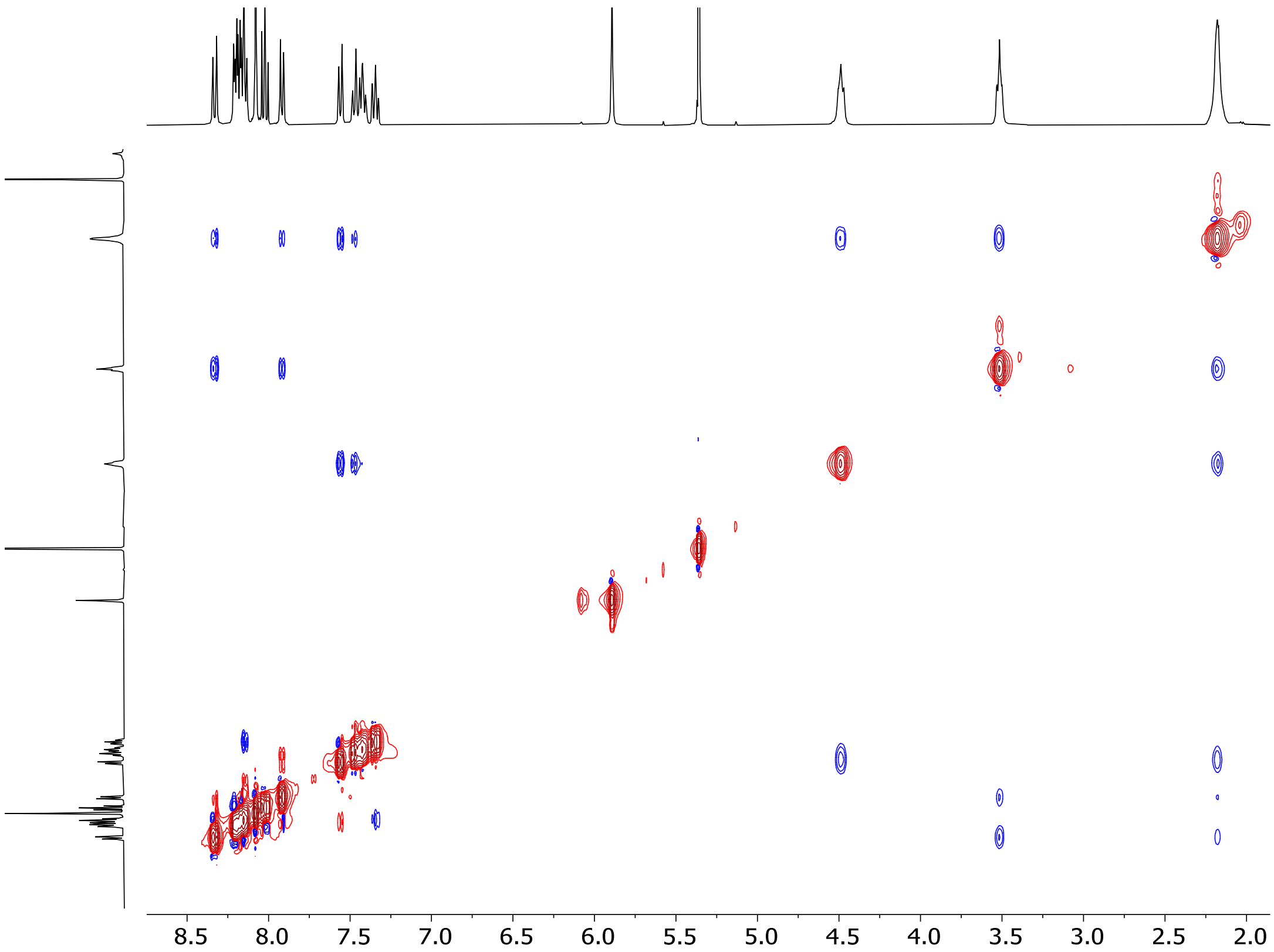

Figure A1-13. NOESY spectrum of [**L2**Y(hfac)$_3$] in CD_2Cl_2.

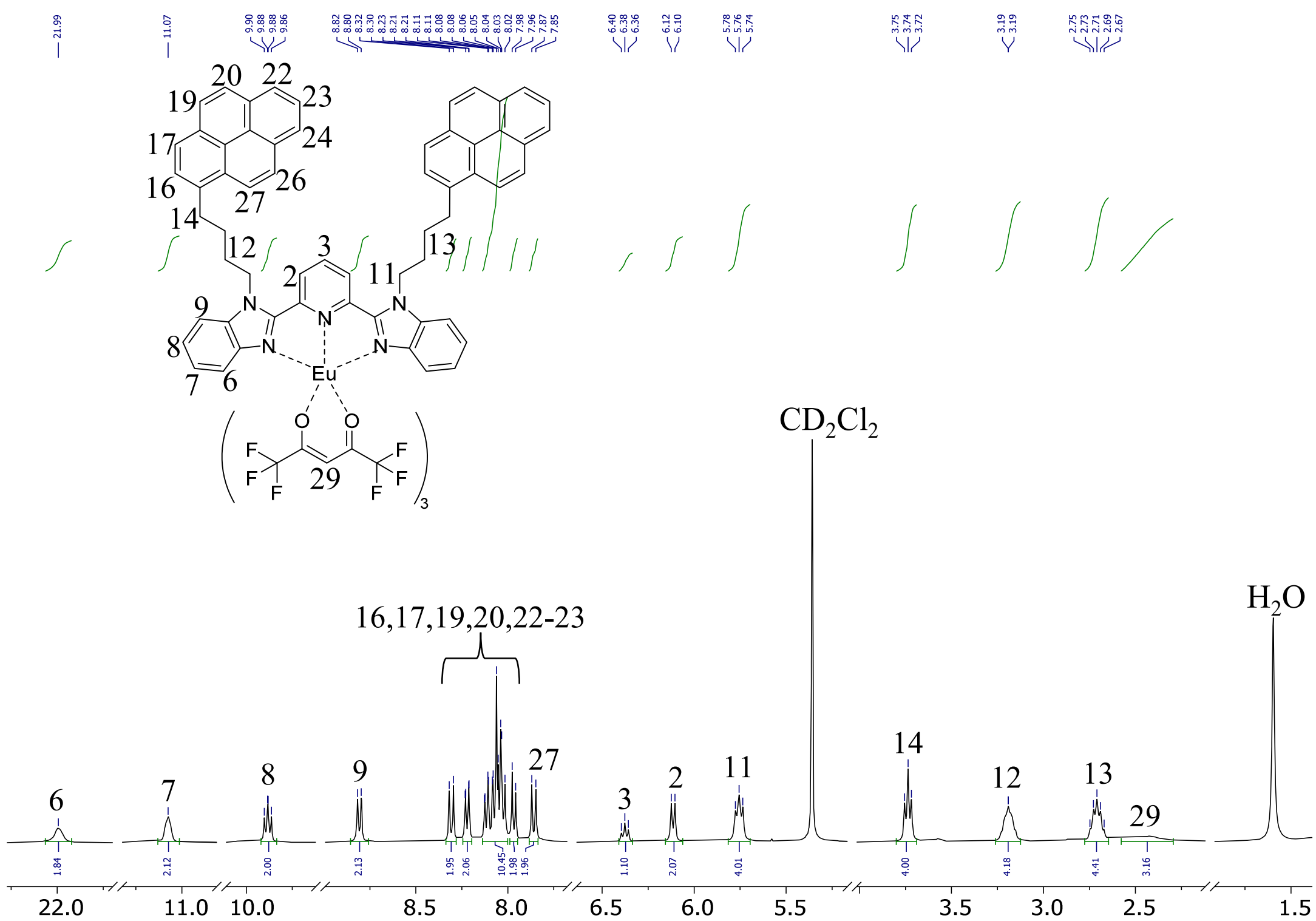

Figure A1-14. ^1H NMR spectrum of [**L2**Eu(hfac)$_3$] in CD_2Cl_2.

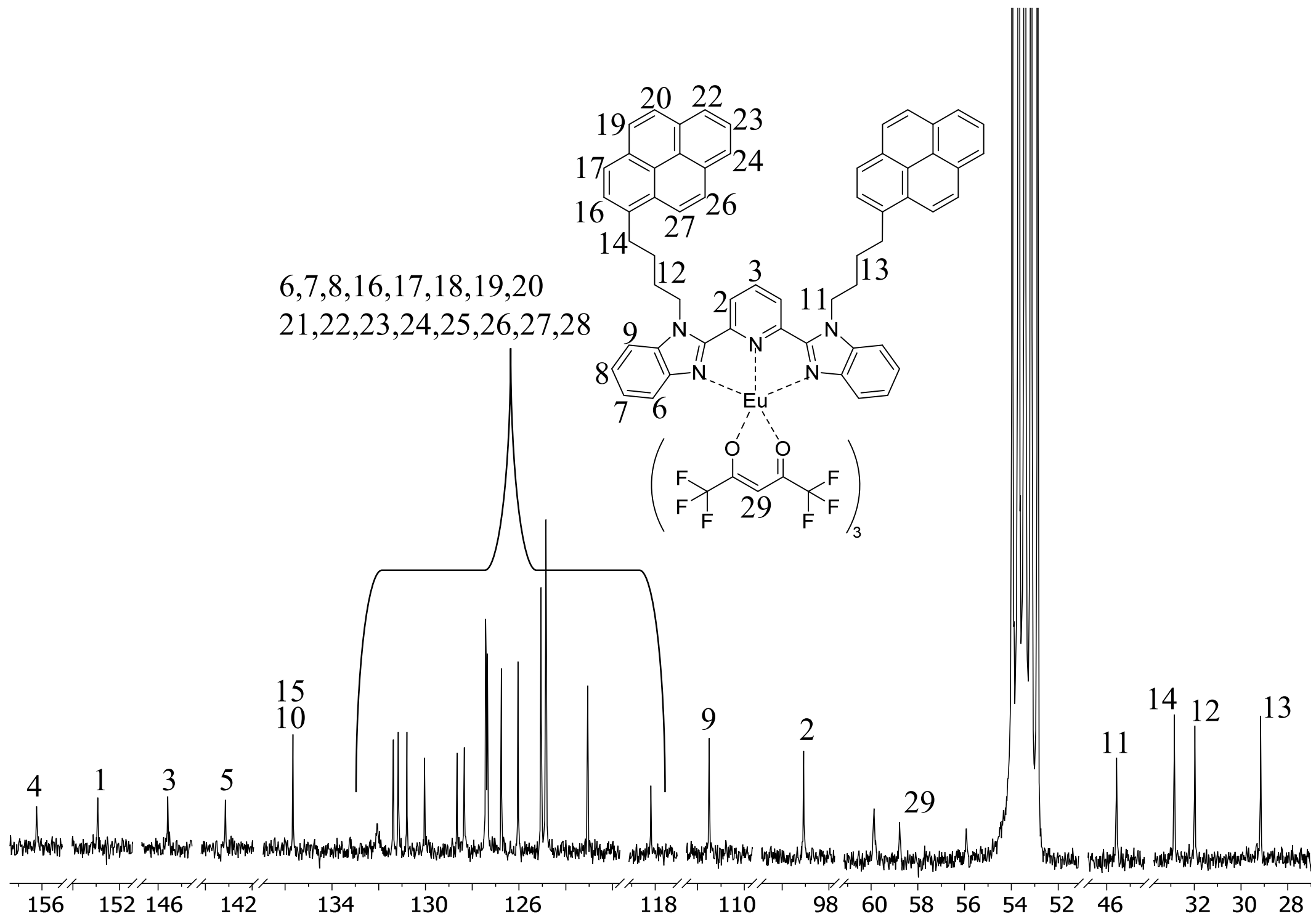

Figure A1-15. ^{13}C NMR spectrum of [**L2**Eu(hfac)$_3$] in CD_2Cl_2.

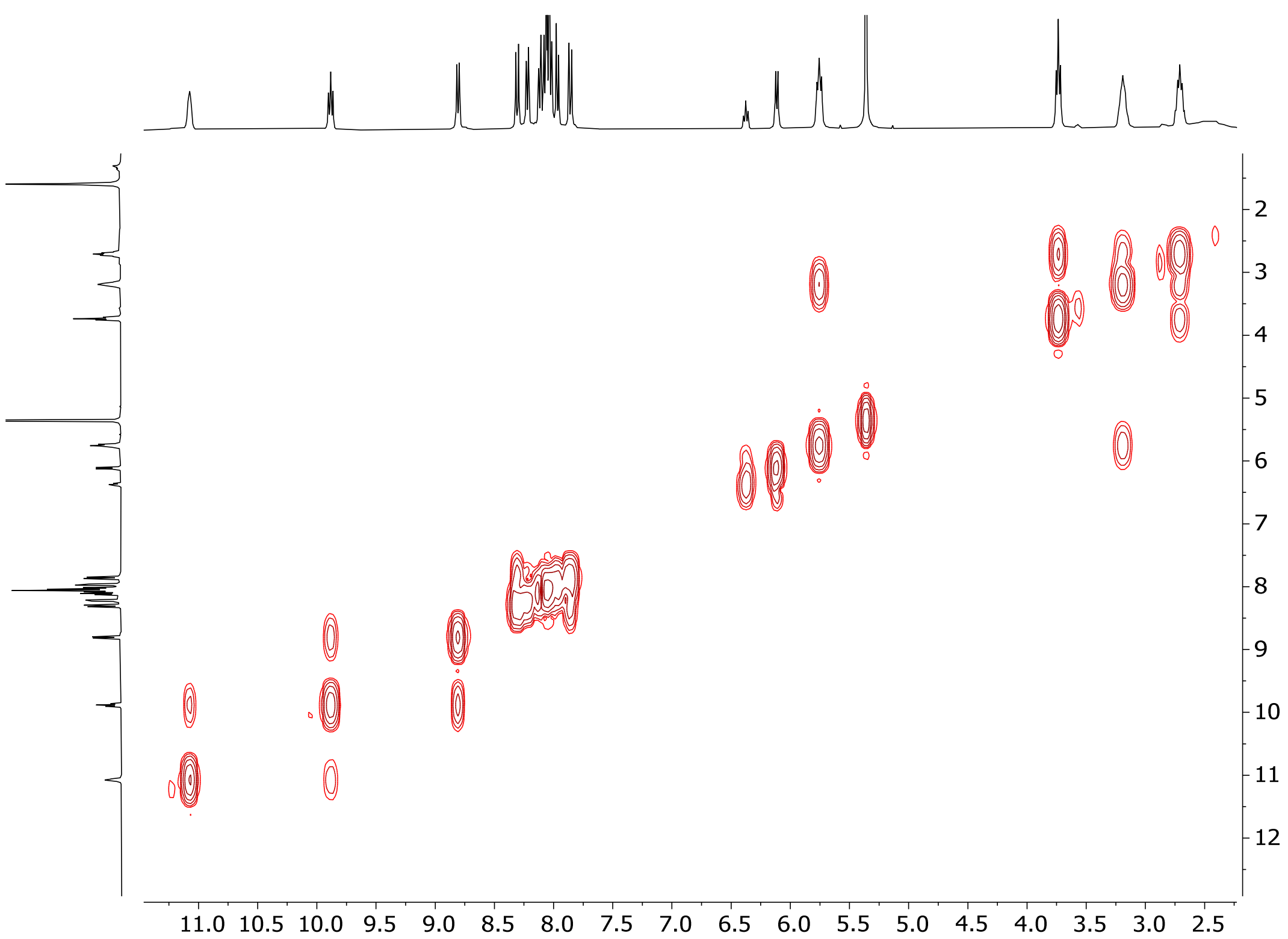

Figure A1-16. COSY spectrum of [**L2**Eu(hfac)$_3$] in CD_2Cl_2.

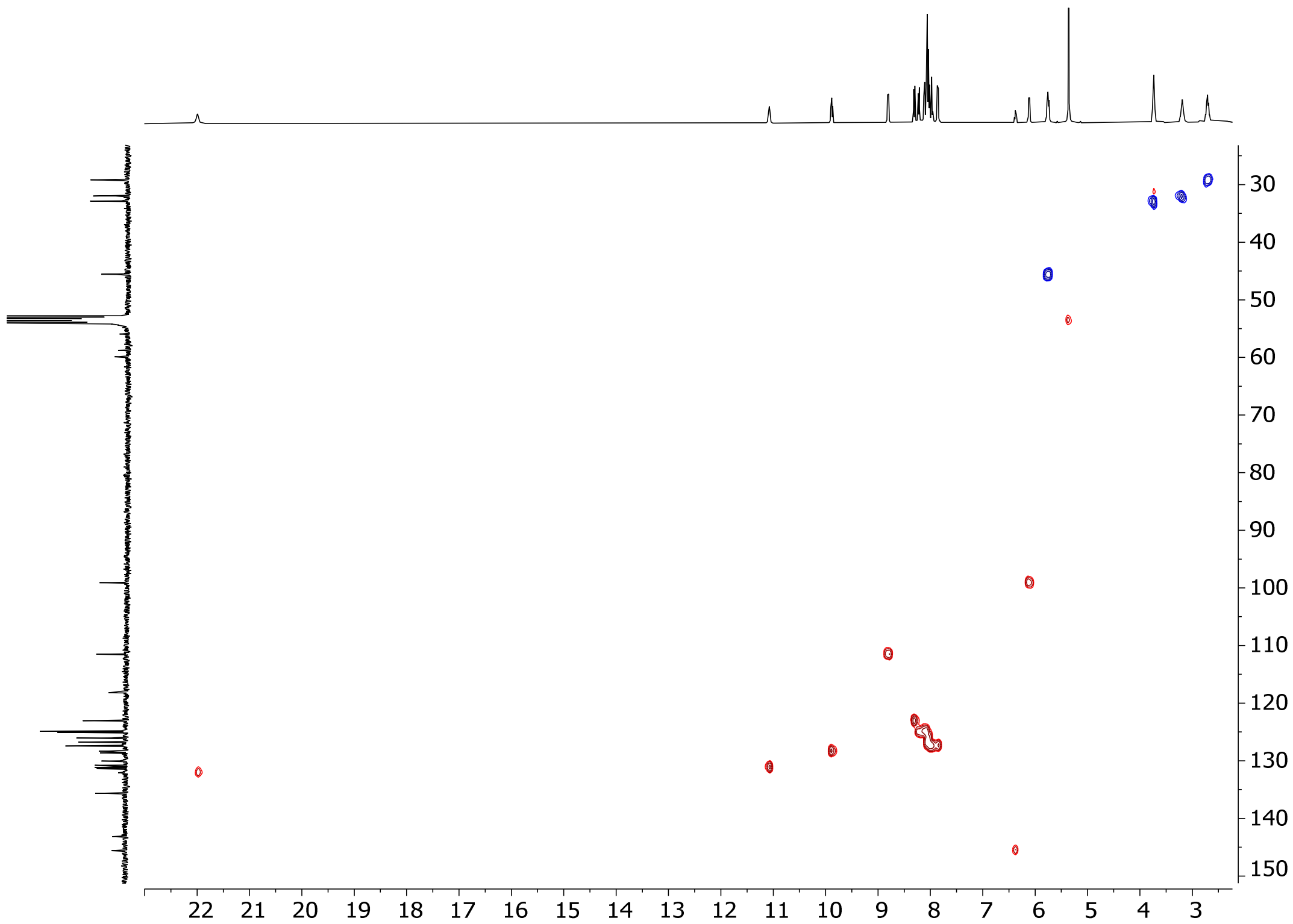

Figure A1-17. HSQC spectrum of [**L2**Eu(hfac)$_3$] in CD_2Cl_2.

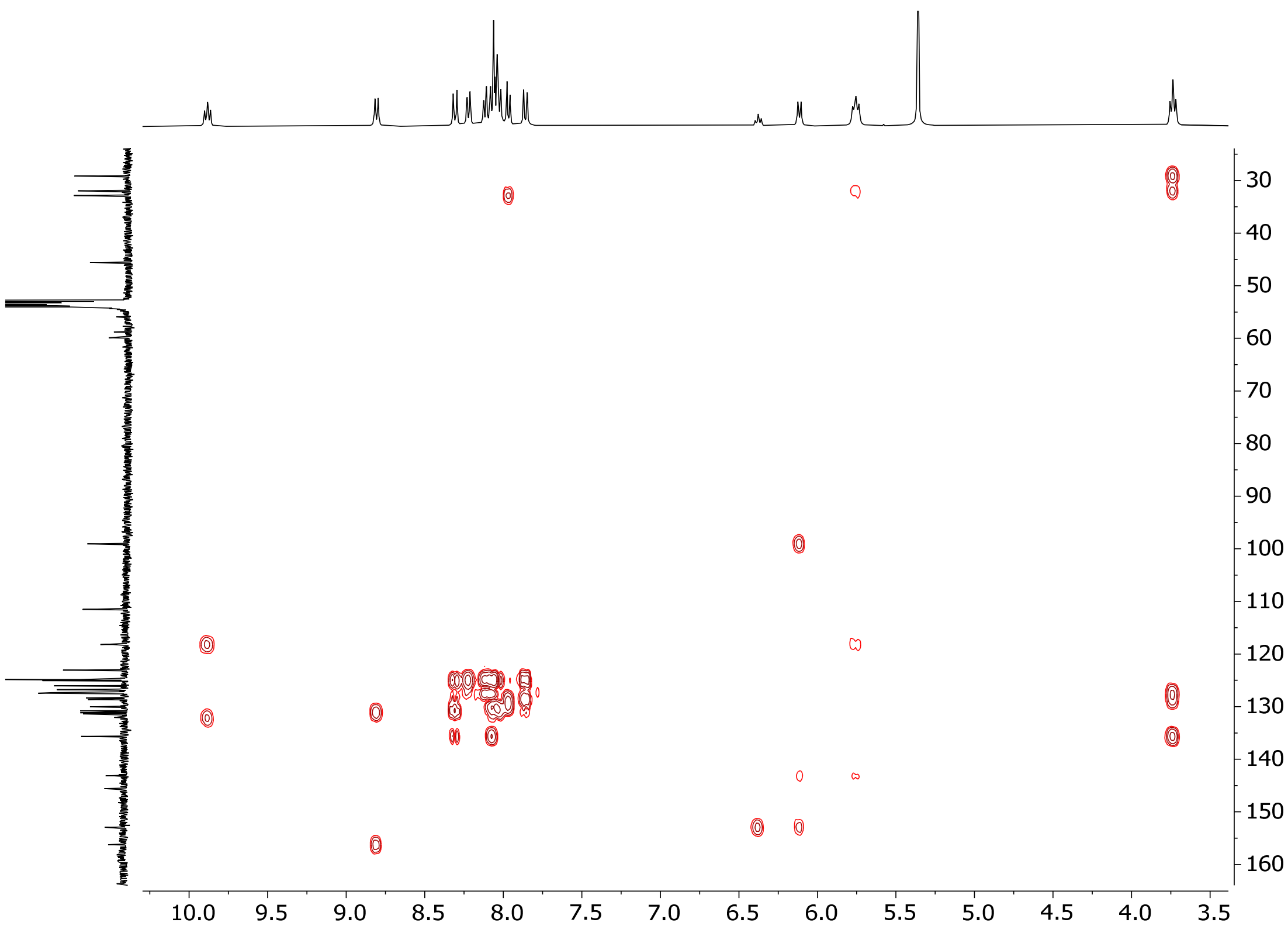

Figure A1-18. HMBC spectrum of [**L2**Eu(hfac)$_3$] in CD_2Cl_2.

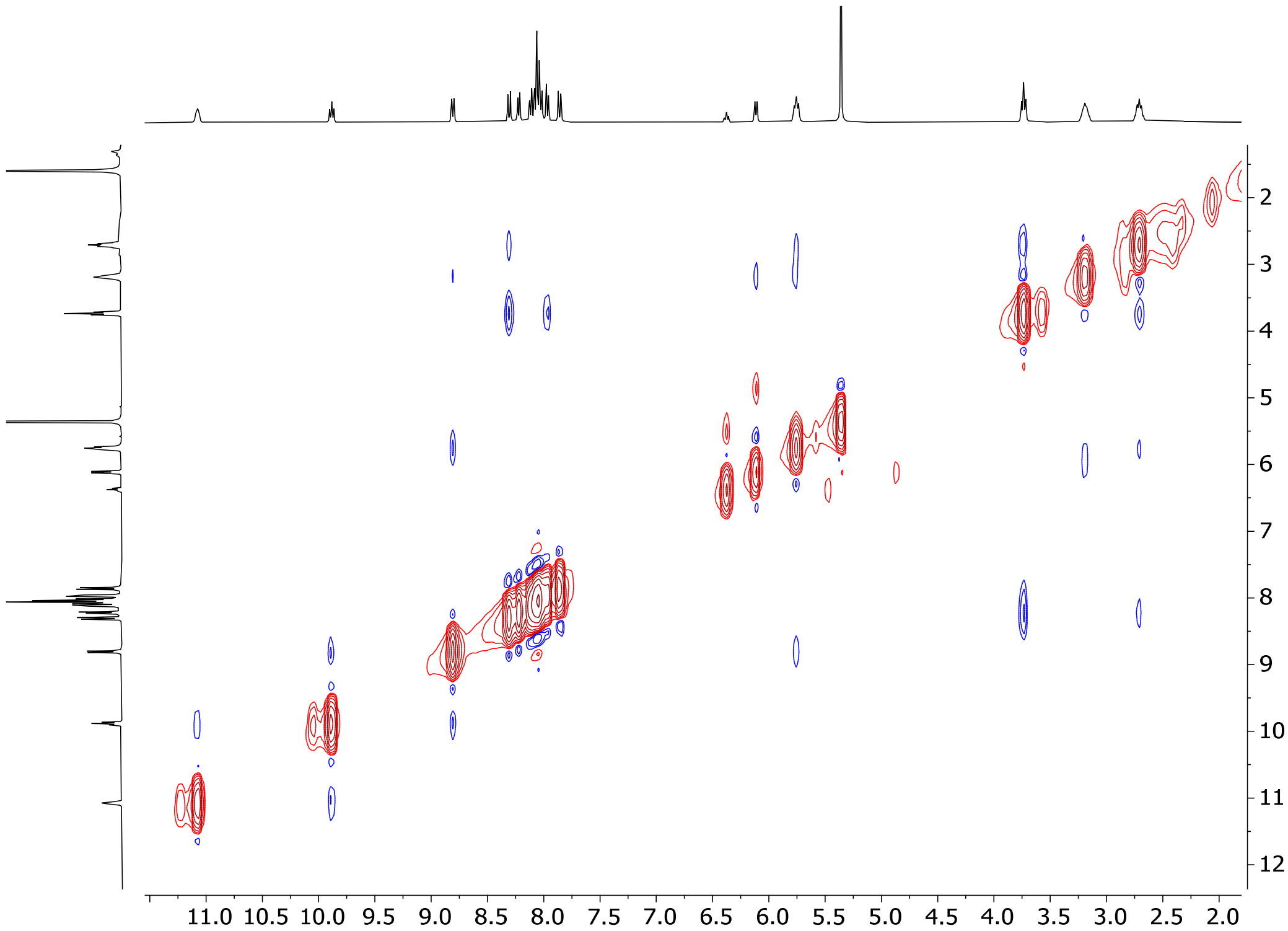

Figure A1-19. NOESY spectrum of [**L2**Eu(hfac)$_3$] in CD_2Cl_2.

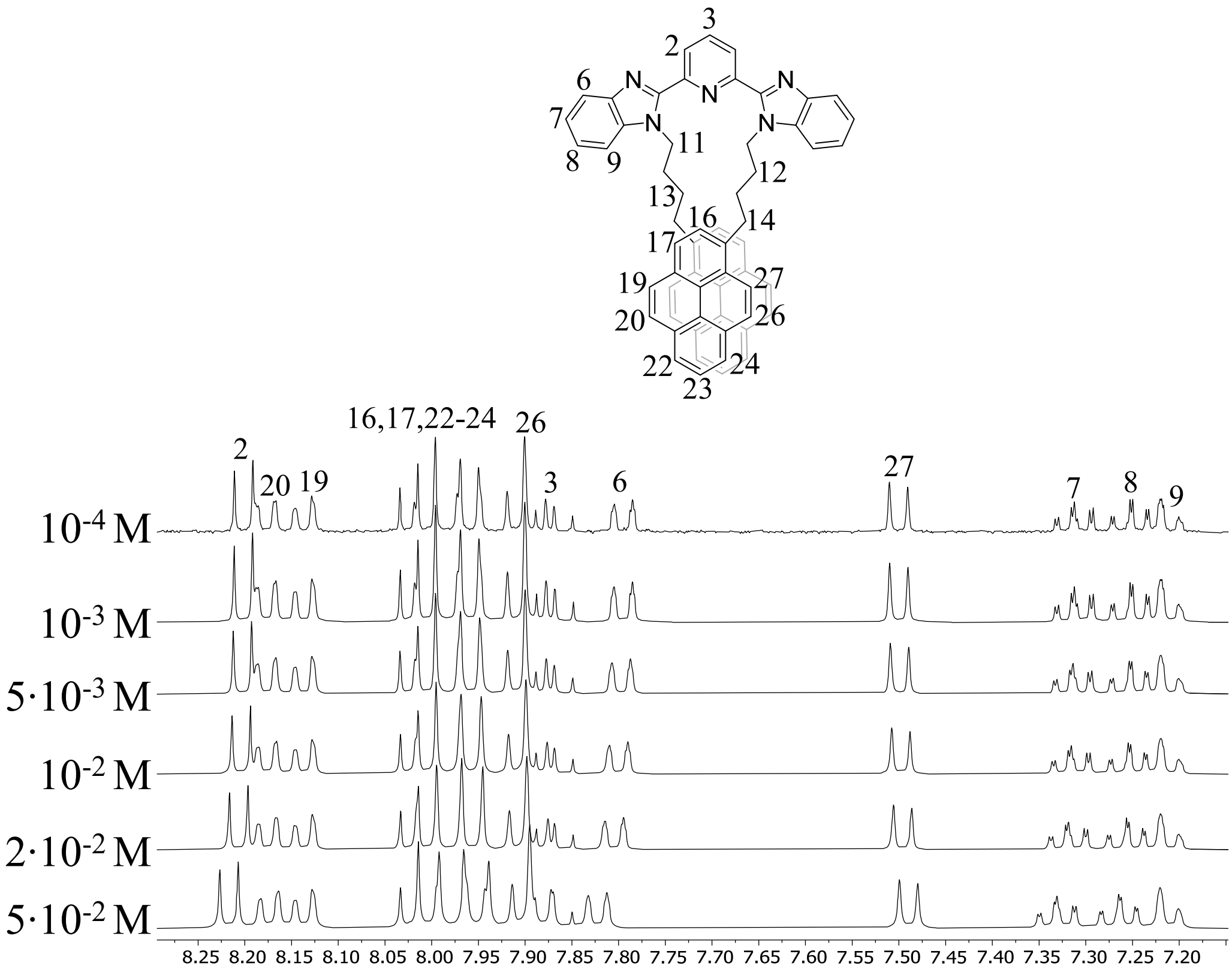

Figure A1-20. ^{1}H NMR spectrum of **L2** in CD_2Cl_2 upon increasing concentration.

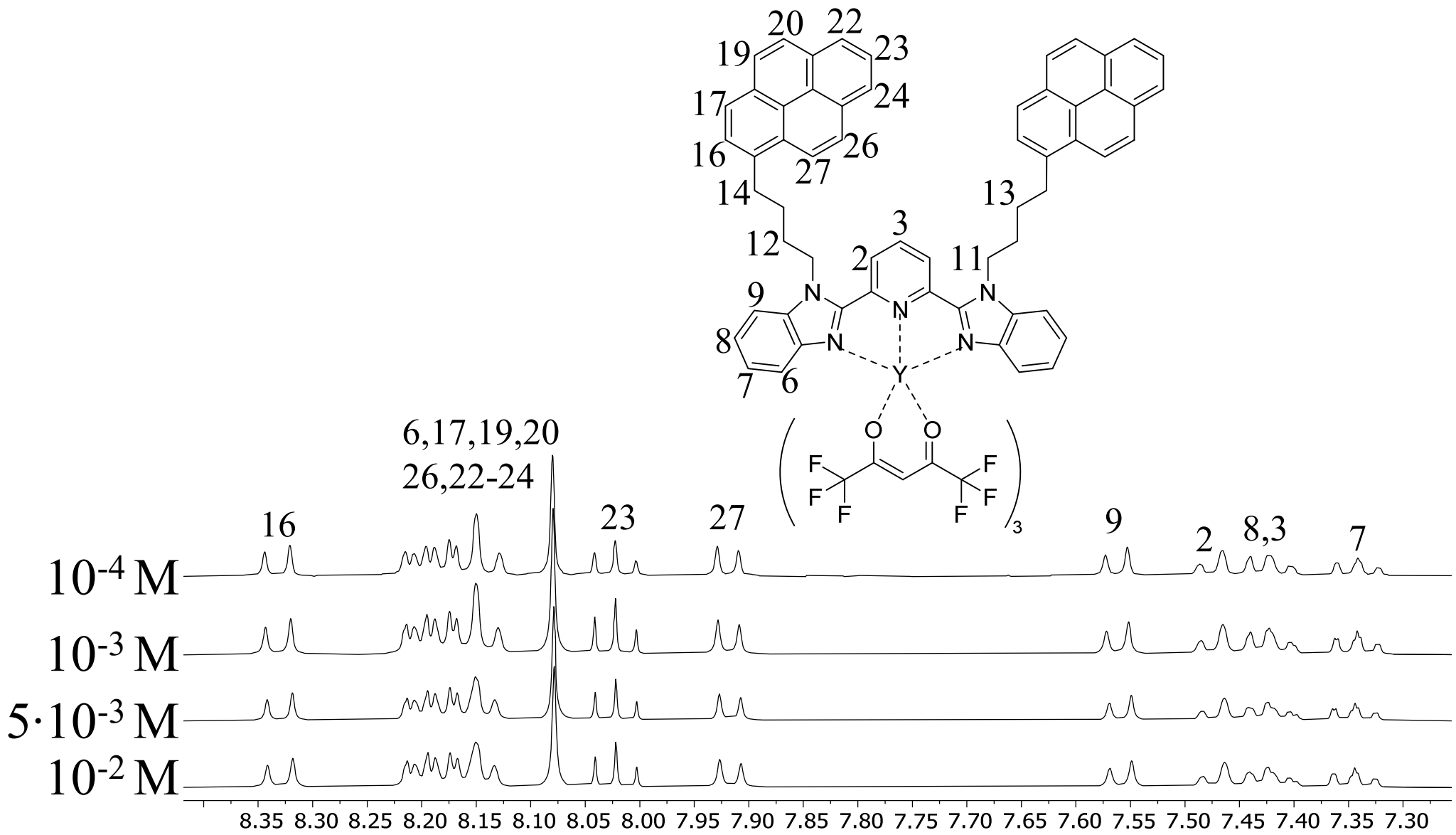

Figure A1-21. ^{1}H NMR spectrum of [**L2**Y(hfac)$_3$] in CD_2Cl_2 upon increasing concentration.

Appendix 2: Crystal structures of ligands and lanthanide complexes

Table A2-1. Summary of crystal data, intensity measurements and structure refinements for **L2**.

Empirical formula	$C_{59}H_{45}N_5$	
Formula weight	824.00	
Temperature	120.00(10) K	
Wavelength	1.54184 Å	
Crystal system	Triclinic	
Space group	P -1	
Unit cell dimensions	a = 12.68874(19) Å	α = 78.8597(10)°.
	b = 12.99923(17) Å	β= 74.9135(11)°.
	c = 13.47662(16) Å	γ = 82.1567(11)°.
Volume	2097.10(5) $Å^3$	
Z	2	
Density (calculated)	1.305 Mg/m^3	
Absorption coefficient	0.591 mm^{-1}	
F(000)	868	
Crystal size	0.39 x 0.37 x 0.13 mm^3	
Theta range for data collection	3.444 to 75.991°.	
Index ranges	-15<=h<=15, -15<=k<=16, -16<=l<=15	
Reflections collected	33426	
Independent reflections	8442 [R(int) = 0.0217]	
Completeness to theta = 67.684°	99.9 %	
Absorption correction	Analytical	
Max. and min. transmission	0.938 and 0.830	
Refinement method	Full-matrix least-squares on F^2	
Data / restraints / parameters	8442 / 0 / 577	
Goodness-of-fit on F^2	1.013	
Final R indices [I>2sigma(I)]	R1 = 0.0409, wR2 = 0.1024	
R indices (all data)	R1 = 0.0445, wR2 = 0.1049	
Largest diff. peak and hole	0.332 and -0.214 e.$Å^{-3}$	

Table A2-2. Selected least-squares planes data of **L2**.

	Abbreviation	RMSD (Å)	Max deviation (Å) (Atom)
Benzimidazole (1)<br>N2 C7 N3 C1 C6 C5 C4 C3 C2	Bz1	0.012	N3, C7 (0.016)
Benzimidazole (2)<br>N5 C13 N4 C14 C19 C18 C17 C16 C15	Bz2	0.006	N5, C18 (0.011)
Pyridine<br>N1 C8 C9 C10 C11 C12	Py	0.005	C9, C12 (0.006)

Table A2-3. Interplanar angles (°) for **L2**.

Plane	Benzimidazole (1)	Benzimidazole (2)
Pyridine	24.81(4)	20.84(4)
Benzimidazole (1)		40.75(3)

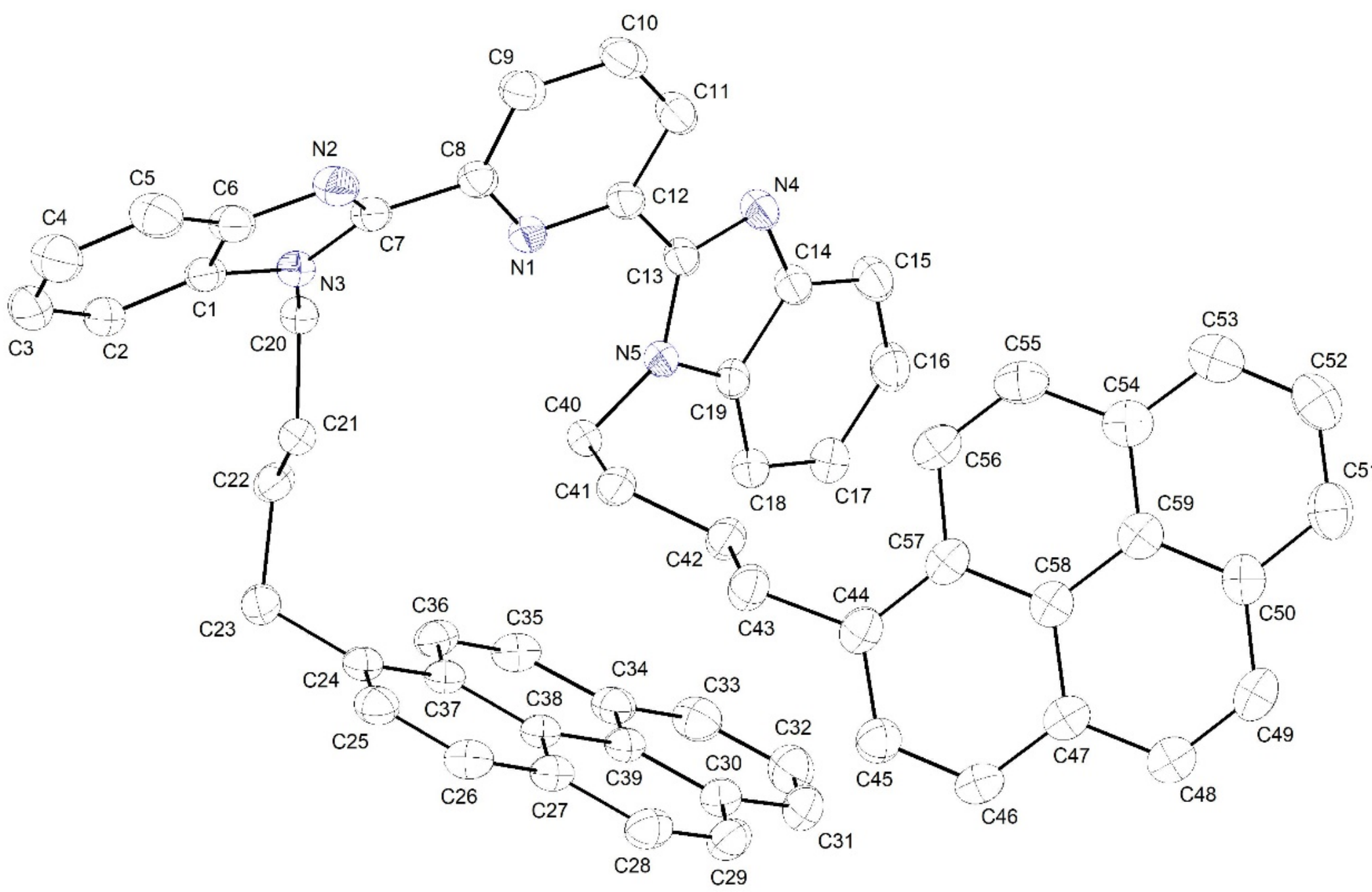

Figure A2-1. ORTEP view of molecule A in **L2** (thermal ellipsoids are drawn at 50% probability) with numbering scheme. Hydrogen atoms are omitted for clarity reasons.

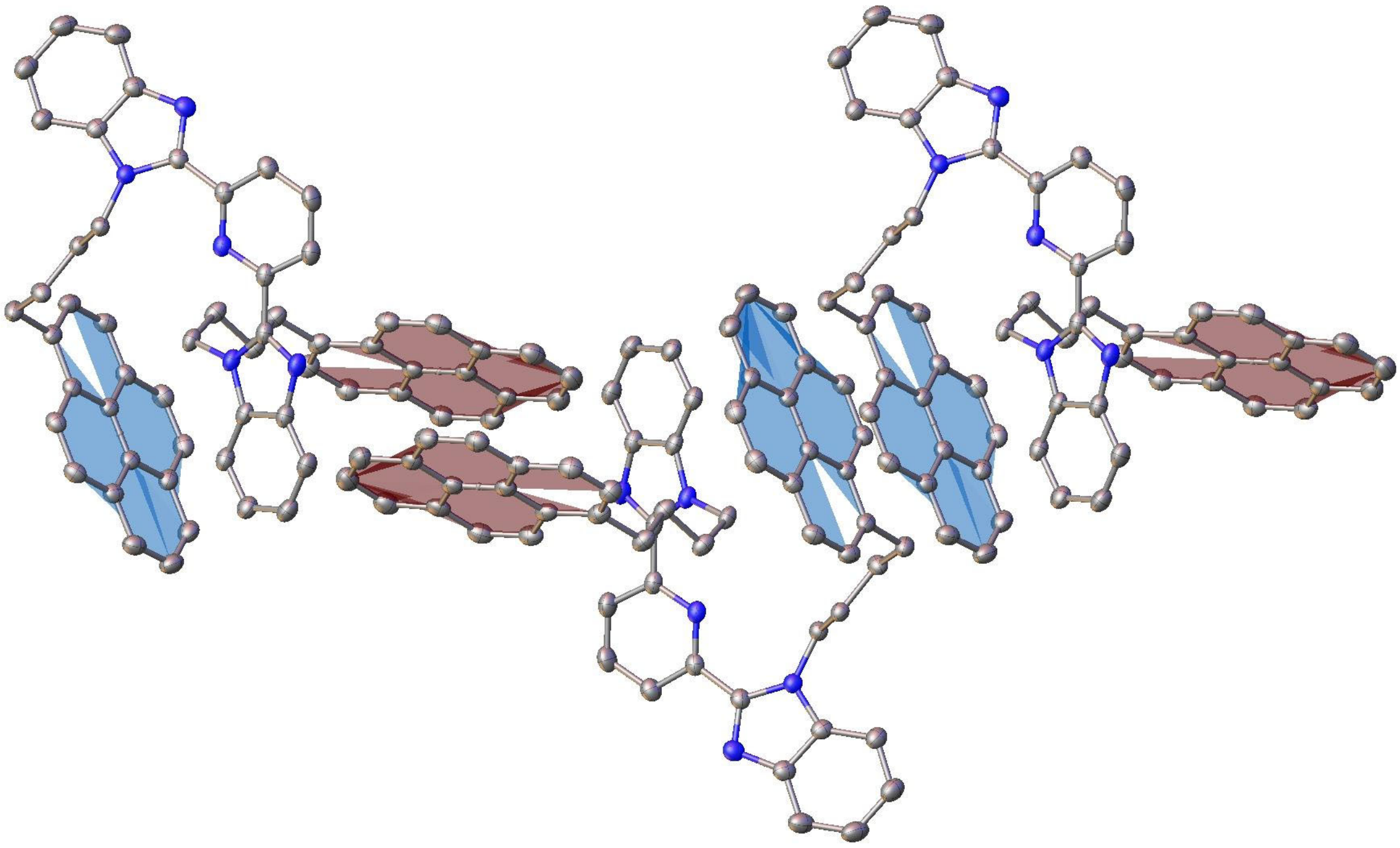

Figure A2-2. Planes and π-π stacking analysis in **L2**.

Brown planes: angle = 0°, distance centroid to plane = 3.5628(7) A.

Blue planes: angle = 0°, distance centroid to plane = 3.4118(8) A.

Table A2-4. Summary of crystal data, intensity measurements and structure refinements for [**L2**Eu(hfac)$_3$]·0.266CH_2Cl_2.

Empirical formula	$C_{74.27}H_{48.53}Cl_{0.53}EuF_{18}N_5O_6$	
Chemical formula moiety	$C_{74}H_{48}F_{18}N_6O_7Eu$·0.266$CH_2Cl_2$	
Formula weight	1619.76	
Temperature	120.00(10) K	
Wavelength	1.54184 Å	
Crystal system	Monoclinic	
Space group	P 2_1/n	
Unit cell dimensions	a = 11.40287(7) Å	α = 90°.
	b = 26.04523(19) Å	β = 99.8738(6)°.
	c = 23.04829(15) Å	γ = 90°.
Volume	6743.73(8) Å^3	
Z	4	
Density (calculated)	1.595 Mg/m^3	
Absorption coefficient	7.811 mm^{-1}	
F(000)	3245	
Crystal size	0.27 x 0.17 x 0.05 mm^3	
Theta range for data collection	2.582 to 75.896°.	
Index ranges	-14<=h<=13, -32<=k<=31, -28<=l<=26	
Reflections collected	64896	
Independent reflections	13576 [R(int) = 0.0331]	
Completeness to theta = 67.684°	99.9 %	
Absorption correction	Analytical	
Max. and min. transmission	0.689 and 0.338	
Refinement method	Full-matrix least-squares on F^2	
Data / restraints / parameters	13576 / 0 / 985	
Goodness-of-fit on F^2	1.040	
Final R indices [I>2sigma(I)]	R1 = 0.0474, wR2 = 0.1180	
R indices (all data)	R1 = 0.0531, wR2 = 0.1211	
Largest diff. peak and hole	0.761 and -0.656 e.Å^{-3}	

Table A2-5. Selected least-squares planes data of [**L2**Eu(hfac)$_3$]·0.266CH_2Cl_2.

	Abbreviation	RMSD (Å)	Max deviation (Å) (Atom)
Benzimidazole (1)<br>N2 C7 N3 C1 C6 C5 C4 C3 C2	Bz1	0.017	N3 (0.029)
Benzimidazole (2)<br>N4 C13 N5 C19 C14 C15 C16 C17 C18	Bz2	0.011	C16 (0.016)
Pyridine<br>N1 C8 C9 C10 C11 C12	Py	0.008	N1, C12 (0.011)

Table A2-6. Interplanar angles (°) for [**L2**Eu(hfac)$_3$]·0.266CH_2Cl_2.

Plane	Benzimidazole (1)	Benzimidazole (2)	Hfac1	Hfac2	Hfac3
Pyridine	28.68(12)	4.44(10)	52.78(14)	30.09(12)	78.4(3)
Benzimidazole (1)		32.99(10)	65.86(13)	20.62(12)	51.0(3)
Benzimidazole (2)			50.46(13)	32.69(11)	82.3(2)
Hfac1				46.65(17)	85.62(15)
Hfac2					52.4(2)

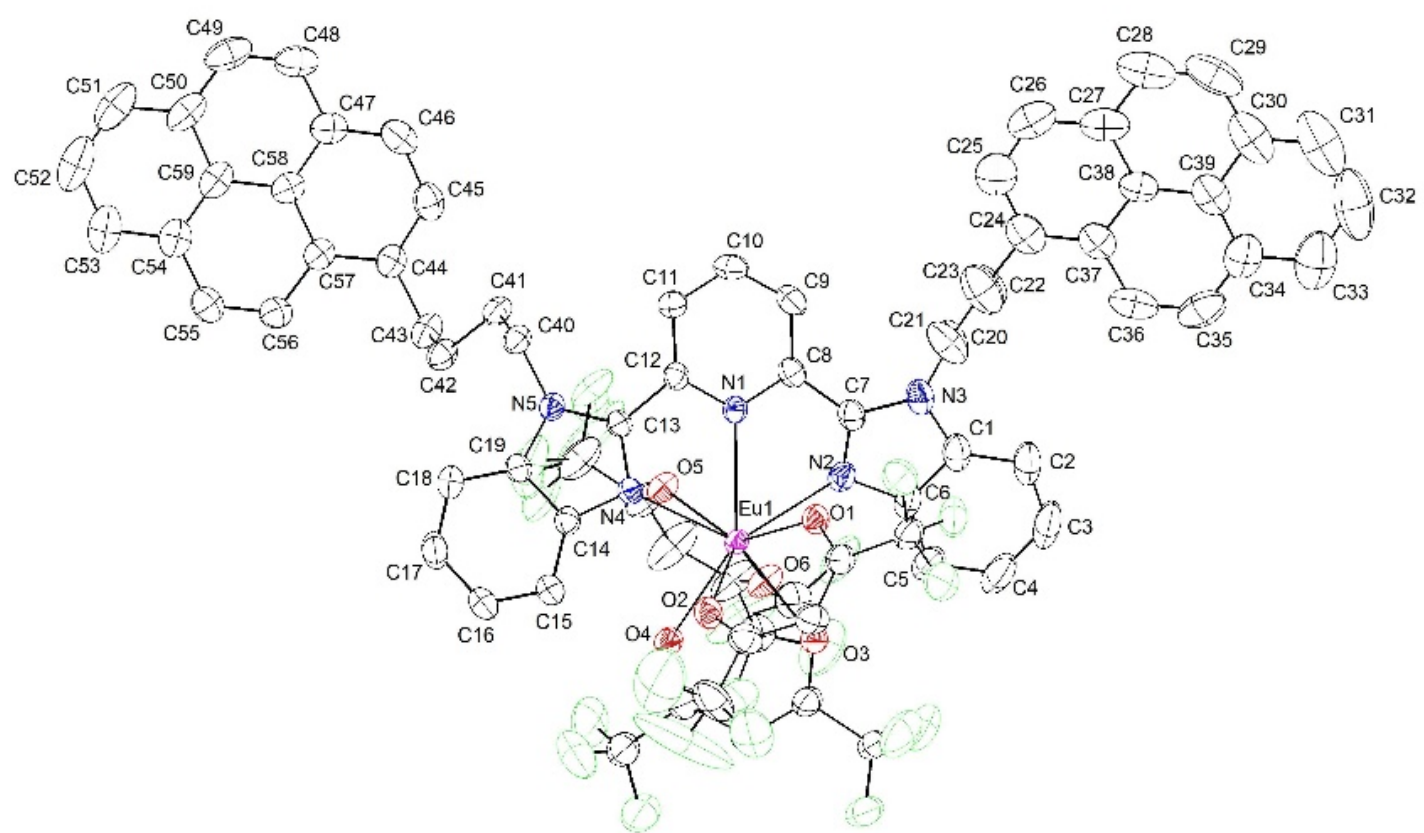

Figure A2-3. ORTEP view of [**L2**Eu(hfac)$_3$] (thermal ellipsoids are drawn at 50% probability level) with numbering scheme of the Ligand and of the Eu coordination sphere. Hydrogren atoms and CH_2Cl_2 solvent molecule are omitted for clarity reasons.

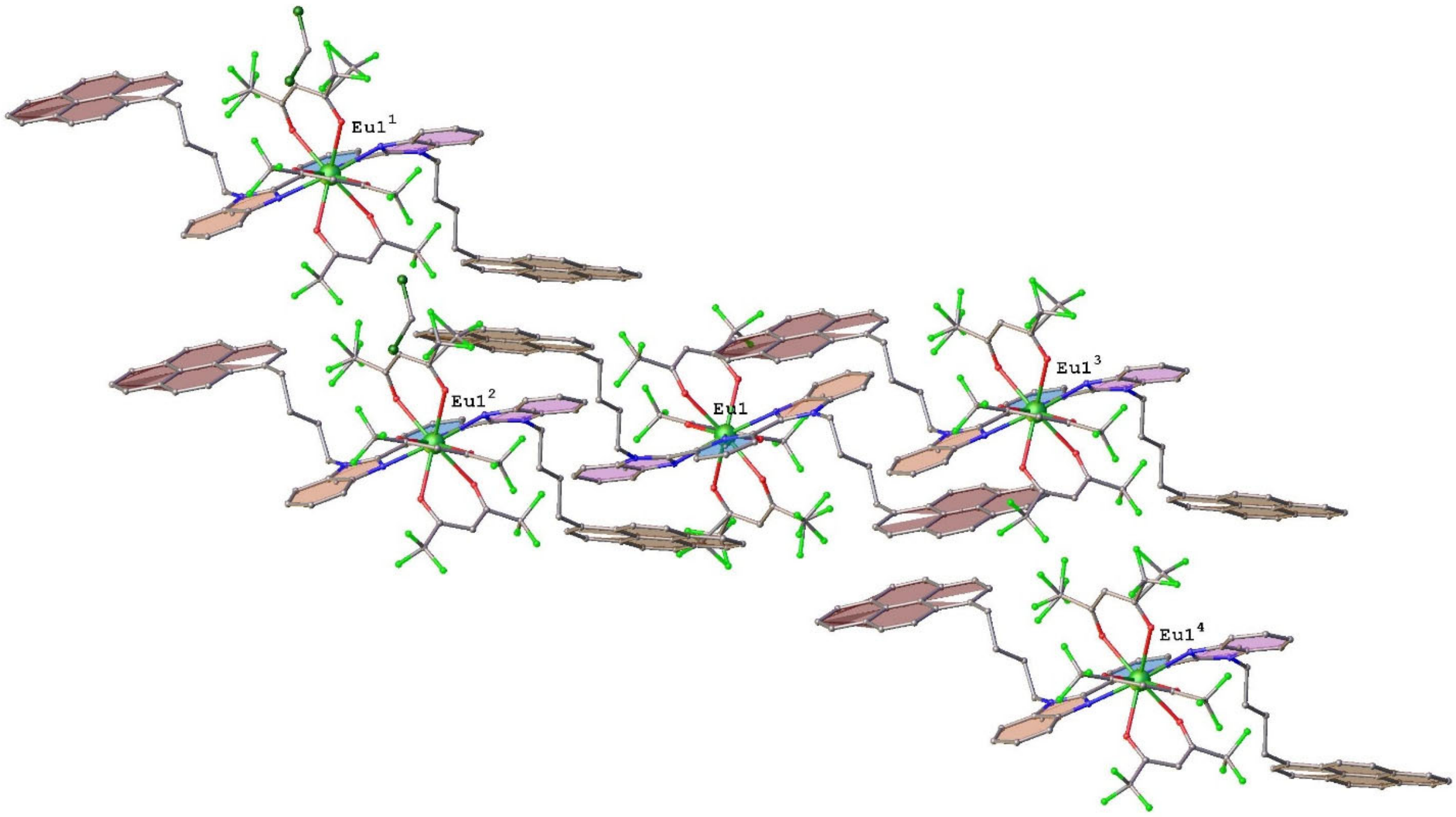

Figure A2-4. Planes and π-π stacking in [**L2**Eu(hfac)$_3$] (Brown = pyrene 1; reddish-brown = pyrene 2; pink = benzimidazole 1 (Bz1); blue = pyridine (py); orange = benzimidazole 2 (Bz2). Sym. op. (1) -x,1-y,-z ; (2) 1-x,1-y,-z ; (3) 2-x,1-y,1-z ; (4) 3-x,1-y,1-z.

Table A2-7. Angle, distance and overlapped areas between aromatic planes in π-stacking of [**L2**Eu(hfac)$_3$] described in Figure.

	Angle (°)	Distance (Å)	Overlap (%)
Pyrene1-pyrene1(1)	0	3.42	31
Pyrene1-Bz1(2)	8.83	3.22	33
Pyrene1-py(2)	30.88	3.90	33
Pyrene2-pyrene2(4)	0	3.58	31
Pyrene2-Bz2(3)	3.79	3.44	33
Pyrene2-py(3)	6.21	3.51	33
Bz1-pyrene1(2)	8.83	3.58	33
Py-pyrene1(2)	30.88	2.44	33
Bz2-pyrene2(3)	3.79	3.39	33
Py-pyrene2(3)	6.21	3.37	33

Sym. op. (1) -x,1-y,-z ; (2) 1-x,1-y,-z ; (3) 2-x,1-y,1-z ; (4) 3-x,1-y,1-z.

Table A2-8. Summary of crystal data, intensity measurements, and structure refinements for [**L2**Y(hfac)$_3$]·0.233CH_2Cl_2.

Empirical formula	$C_{74.23}H_{48.47}Cl_{0.46}F_{18}N_5O_6Y$	
Chemical formula moiety	$C_{74}H_{48}F_{18}N_5O_6Y$·0.233$CH_2Cl_2$	
Formula weight	1553.83	
Temperature	120.00(10) K	
Wavelength	1.54184 Å	
Crystal system	Monoclinic	
Space group	P 2_1/n	
Unit cell dimensions	a = 11.34229(6) Å	α = 90°.
	b = 25.99883(19) Å	β = 99.6609(5)°.
	c = 23.09298(15) Å	γ = 90°.
Volume	6713.23(7) $Å^3$	
Z	4	
Density (calculated)	1.537 Mg/m^3	
Absorption coefficient	2.357 mm^{-1}	
F(000)	3143	
Crystal size	0.22 x 0.12 x 0.06 mm^3	
Theta range for data collection	2.580 to 74.283°.	
Index ranges	-14<=h<=11, -32<=k<=31, -27<=l<=28	
Reflections collected	65557	
Independent reflections	13469 [R(int) = 0.0212]	
Completeness to theta = 67.684°	99.9 %	
Absorption correction	Analytical	
Max. and min. transmission	0.885 and 0.744	
Refinement method	Full-matrix least-squares on F^2	
Data / restraints / parameters	13469 / 8 / 996	
Goodness-of-fit on F^2	1.023	
Final R indices [I>2sigma(I)]	R1 = 0.0468, wR2 = 0.1256	
R indices (all data)	R1 = 0.0509, wR2 = 0.1286	
Largest diff. peak and hole	1.090 and -0.778 e.$Å^{-3}$	

Table A2-9. Selected least-squares planes data of [**L2**Y(hfac)$_3$]·0.233CH_2Cl_2.

	Abbreviation	RMSD (Å)	Max deviation (Å) (Atom)
Benzimidazole (1)<br>N1 C7 N2 C6 C1 C2 C3 C4 C5	Bz1	0.010	C7 (0.016)
Benzimidazole (2)<br>N4 C13 N5 C14 C19 C18 C17 C16 C15	Bz2	0.019	N4, N5 (0.031)
Pyridine<br>N3 C12 C11 C10 C9 C8	Py	0.008	C11 (0.011)

Table A2-10. Interplanar angles (°) for [**L2**Y(hfac)$_3$]·0.233CH_2Cl_2.

Plane	Benzimidazole (1)	Benzimidazole (2)	Hfac1	Hfac2	Hfac3
Pyridine	27.37(8)	4.48(7)	77.69(12)	30.26(8)	54.0(1)
Benzimidazole (1)		31.67(7)	51.89(12)	22.20(8)	66.68(9)
Benzimidazole (2)			81.37(11)	32.45(8)	51.39(9)
Hfac1				52.08(13)	84.48(10)
Hfac2					46.19(12)

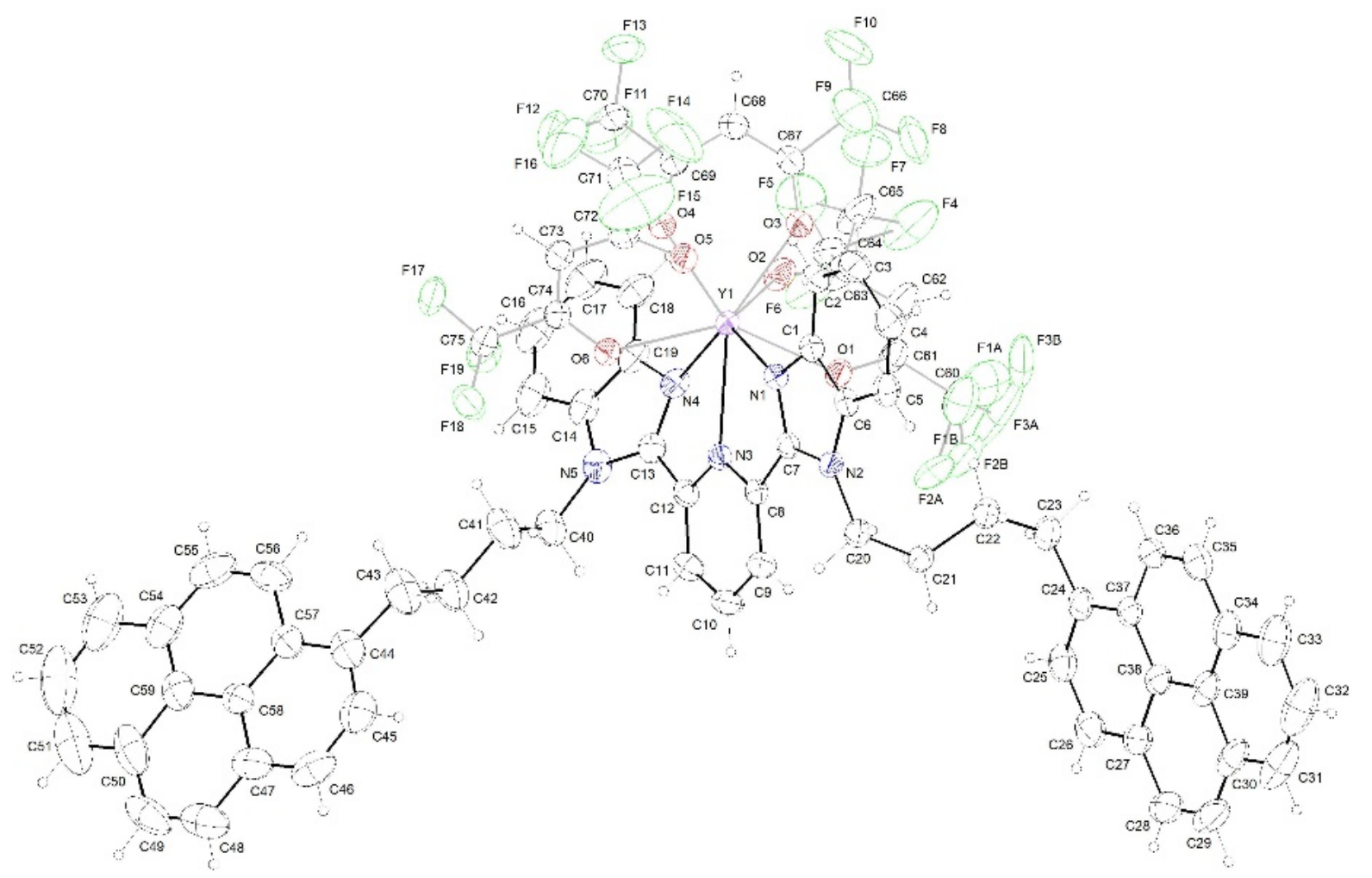

Figure A2-5. ORTEP view of [**L2**Y(hfac)$_3$] (thermal ellipsoids are drawn at 50% probability level) with numbering scheme.

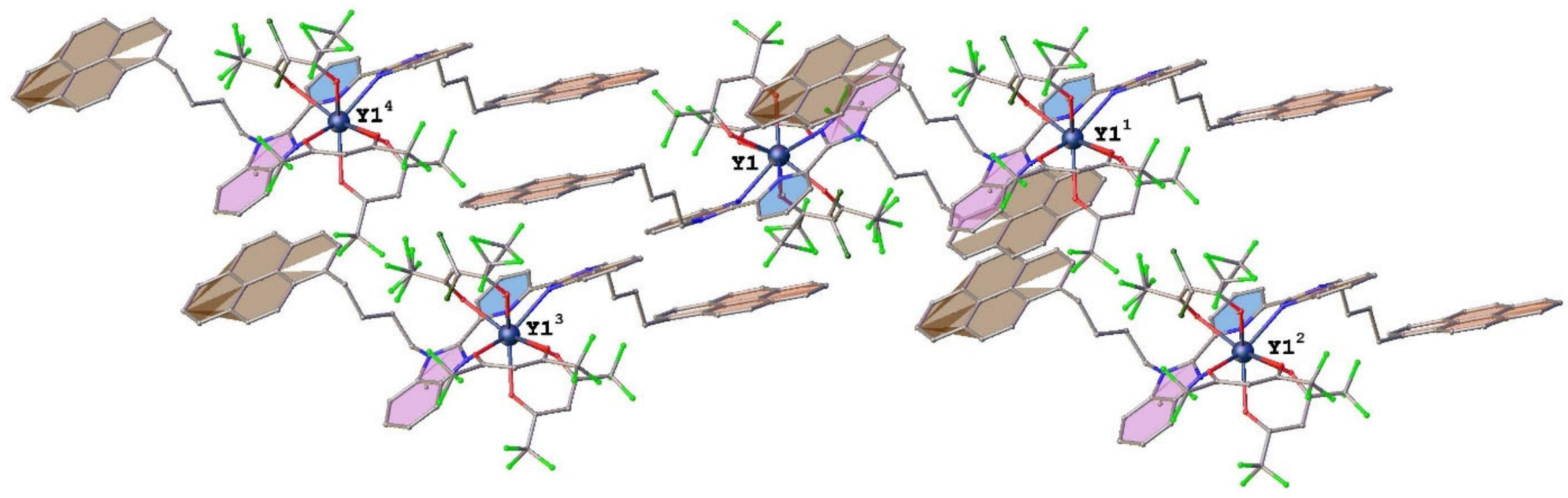

Figure A2-6. Planes and π-π stacking in [**L2**Y(hfac)$_3$] (Brown = pyrene 1; reddish-brown = pyrene 2; pink = benzimidazole 1 (Bz1); blue = pyridine (py); orange = benzimidazole 2 (Bz2). Sym. op. (1) 2-x,1-y,1-z ; (2) 3-x,1-y,1-z ; (3) 1-x,1-y,-z ; (4) -x,1-y,-z.

Table A2-11. Angle, distance, and overlap between aromatic planes in π-stacking of [**L2**Y(hfac)$_3$] described in Figure.

	Angle (°)	Distance (Å)	Overlap (%)
Pyrene1-pyrene1(2)	0	3.57	25
Pyrene1-Bz1(1)	4.35	3.44	33
Bz1-pyrene1(1)	4.35	3.38	33
Py-pyrene2(3)	30.07	3.90	33
Bz2-pyrene2(3)	9.22	3.23	33
Pyrene2-pyrene2(4)	0	3.43	31
Pyrene2-Bz2(3)	9.22	3.23	22
Pyrene2-py (3)	30.07	3.90	33
Py-pyrene1(1)	7.04	3.34	33

Sym. op. (1) 2-x,1-y,1-z ; (2) 3-x,1-y,1-z ; (3) 1-x,1-y,-z ; (4) -x,1-y,-z.

Appendix 3: The aggregation equilibrium constant K_{Dimer} of 1, L1 and [L1Y(hfac)$_3$] in dichloromethane solution.

Table A3-1. The aggregation equilibrium constant K_{Dimer} and the chemical shift δ_D of pure dimer obtained from the titrations of **1** (H^{27}) at various temperatures.

Temperature (K)	K_{Dimer} /M^{-1}	δ_D /ppm
288	4.10	7.9139
298	3.19(3.6)[a]	7.9096(8.3260)[a]
303	0.67	7.8147
308	0.50	7.7812
313	0.34	7.7282

[a]obtained from H^{16}

Table A3-2. Chemical shift of H^{27} in compound **1** upon ^{1}H NMR titration at 288 K.

Concentration (10^{-2}·M)	Observed chemical shift (ppm)
4.9407	7.9379
3.8709	7.9392
3.1738	7.9399
2.7151	7.9406
2.3579	7.9412
2.0965	7.9414
1.8803	7.9417
1.6991	7.9419
10^{-2} (pure monomer)	7.9455

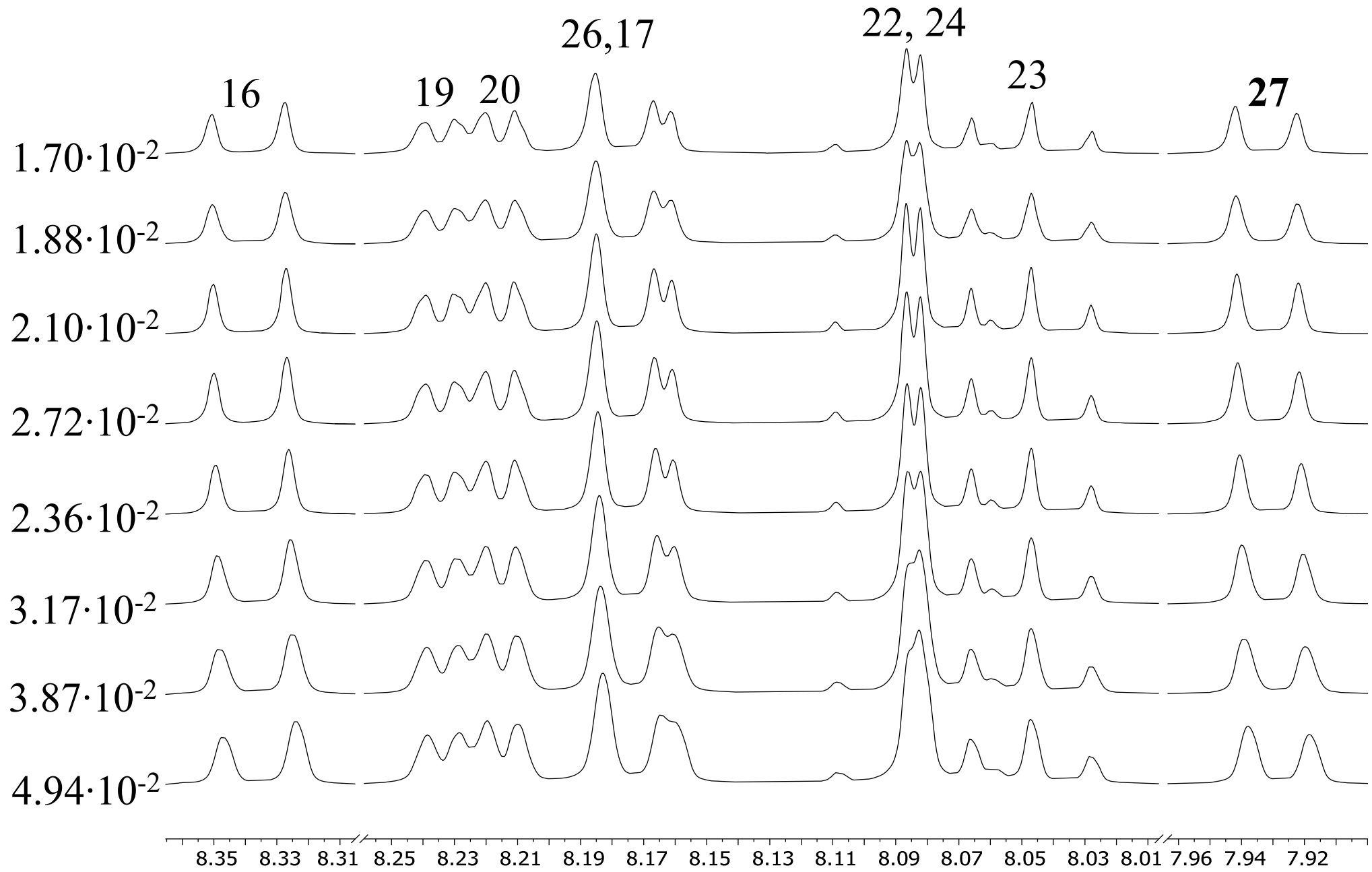

Figure A3-1. ^{1}H NMR spectrum of **1** in CD_2Cl_2 upon decreasing concentration for determining the dimerization constant at 288 K.

Table A3-3. Chemical shift of H^{27} in compound **1** upon ^{1}H NMR titration at 303 K.

Concentration (10^{-2}·M)	Observed chemical shift of H^{27} (ppm)
7.4764	7.9378
5.6867	7.9397
4.5985	7.9417
3.8441	7.9426
3.3134	7.9436
2.8964	7.9442
2.5586	7.9447
2.2885	7.9451
2.0710	7.9454
10^{-2} (pure monomer)	7.9490

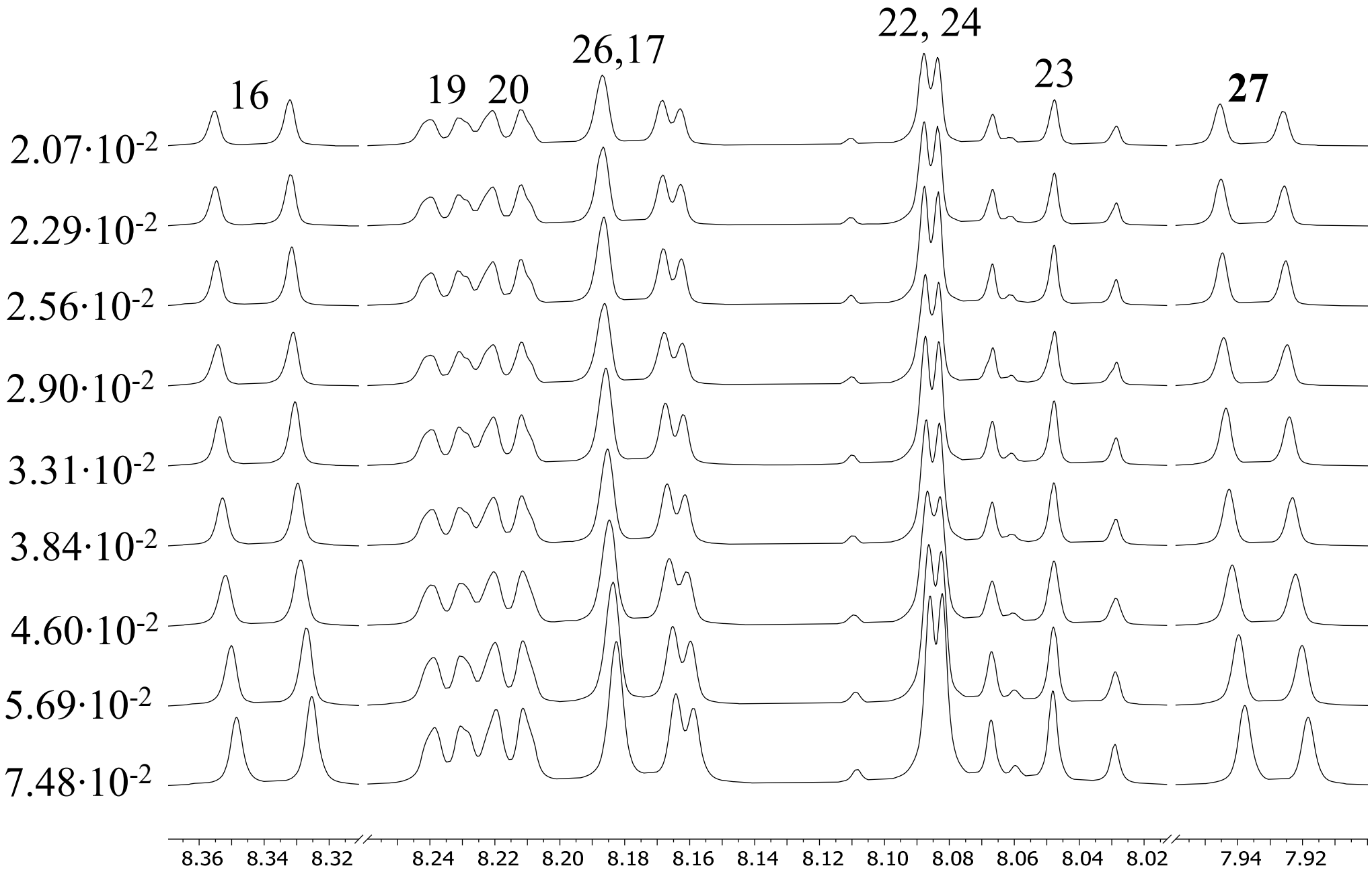

Figure A3-2. ^{1}H NMR spectrum of **1** in CD_2Cl_2 upon decreasing concentration for determining the dimerization constant at 303 K.

Table A3-4. Chemical shift of H^{27} in compound **1** upon ^{1}H NMR titration at 308 K.

Concentration (10^{-2}·M)	Observed chemical shift of H^{27} (ppm)
7.4764	7.9392
5.6867	7.9412
4.5985	7.9431
3.8441	7.9439
3.3134	7.9449
2.8964	7.9456
2.5586	7.9460
2.2885	7.9464
2.0710	7.9468
10^{-2} (pure monomer)	7.9501

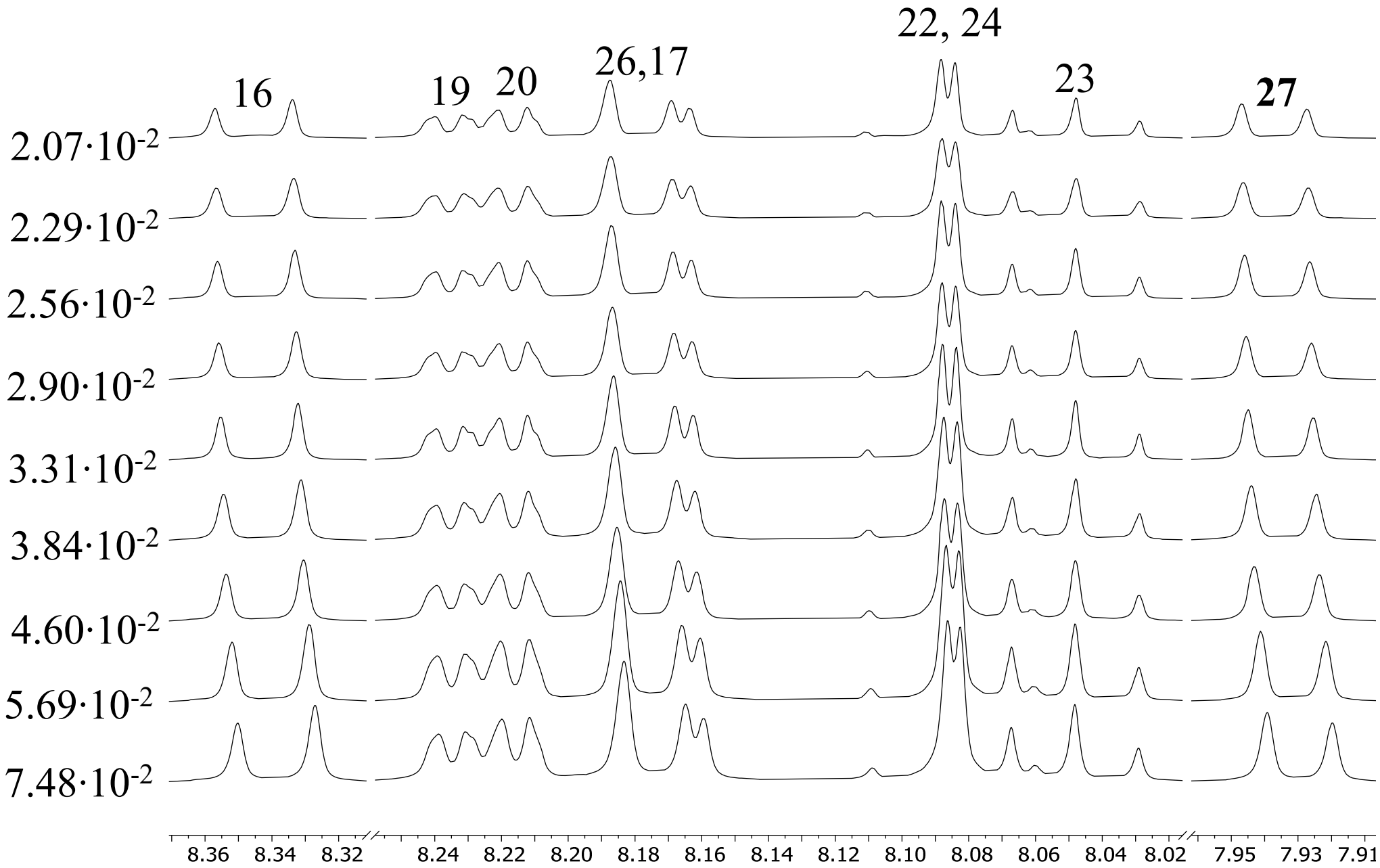

Figure A3-3. ^{1}H NMR spectrum of **1** in CD_2Cl_2 upon decreasing concentration for determining the dimerization constant at 308 K.

Table A3-5. Chemical shift of H^{27} in compound **1** upon ^{1}H NMR titration at 313 K.

Concentration (10^{-2}·M)	Observed chemical shift of H^{27} (ppm)
7.4764	7.9408
5.6867	7.9427
4.5985	7.9445
3.8441	7.9454
3.3134	7.9462
2.8964	7.9469
2.5586	7.9474
2.2885	7.9478
2.0710	7.9481
10^{-2} (pure monomer)	7.9511

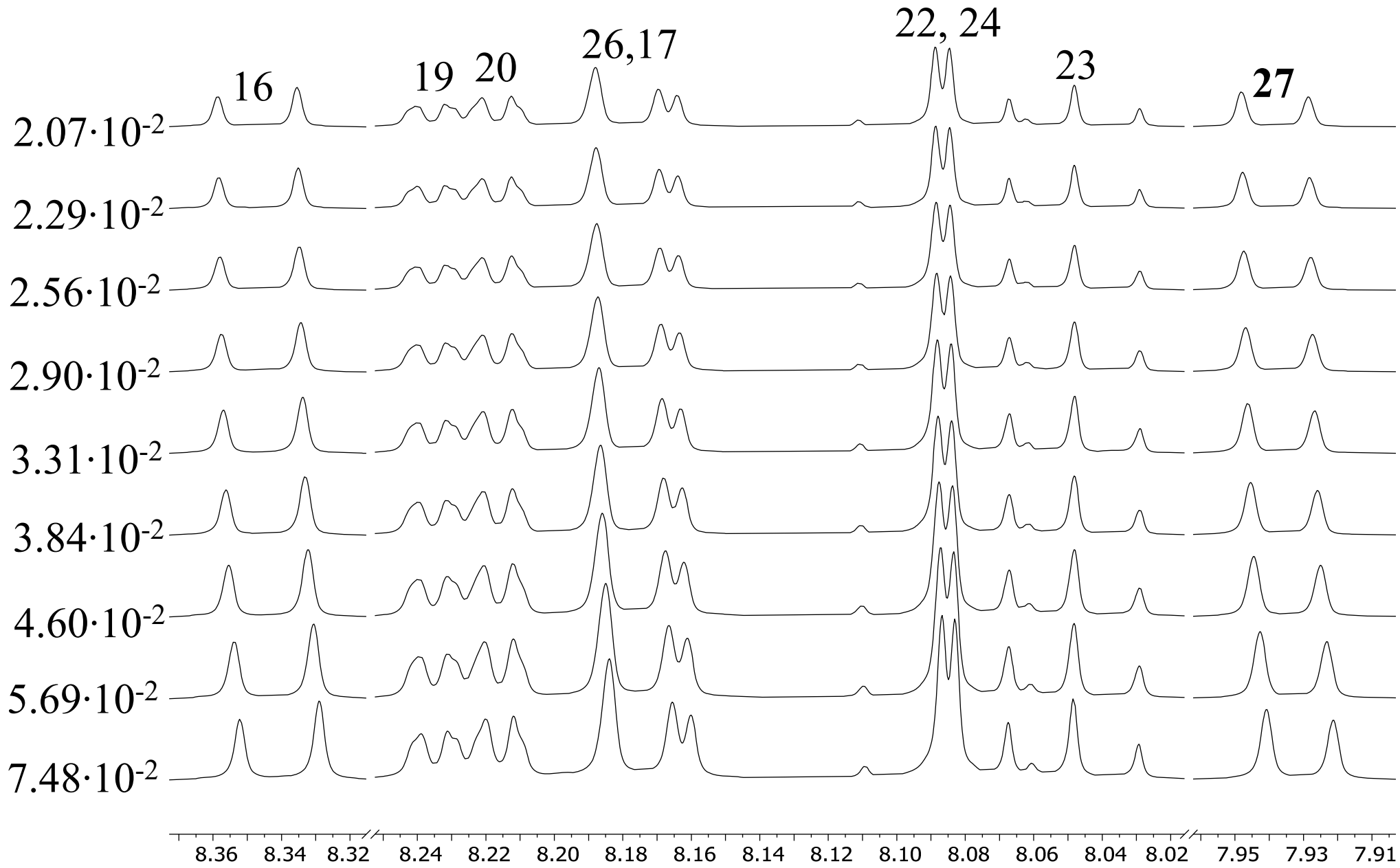

Figure A3-4. ^{1}H NMR spectrum of **1** in CD_2Cl_2 upon decreasing concentration for determining the dimerization constant at 313 K.

Table A3-6. The effective molarity EM, intramolecular aggregation constant K_{Fold} and the chemical shift δ_{Fold} of pure folded conformer obtained from the titrations of **L2** (H^{27}) at various temperatures.

Temperature (K)	K_{Fold}/M^{-1}	δ_{Fold} /ppm	EM
288	19.5125	7.4817	5
298	22.1328	7.4885	7
303	22.9982	7.4929	34
308	23.4994	7.4962	46
313	23.9998	7.4994	71

Table A3-7. Chemical shift of H^{27} in ligand **L2** upon ^1H NMR titration at 288 K.

Concentration (10^{-2}·M)	Observed chemical shift (ppm)
3.9852	7.4940
2.9340	7.4966
2.3374	7.4980
1.9269	7.4992
1.6289	7.5000
1.4238	7.5006
1.2557	7.5011
1.1210	7.5014
1.0152	7.5017

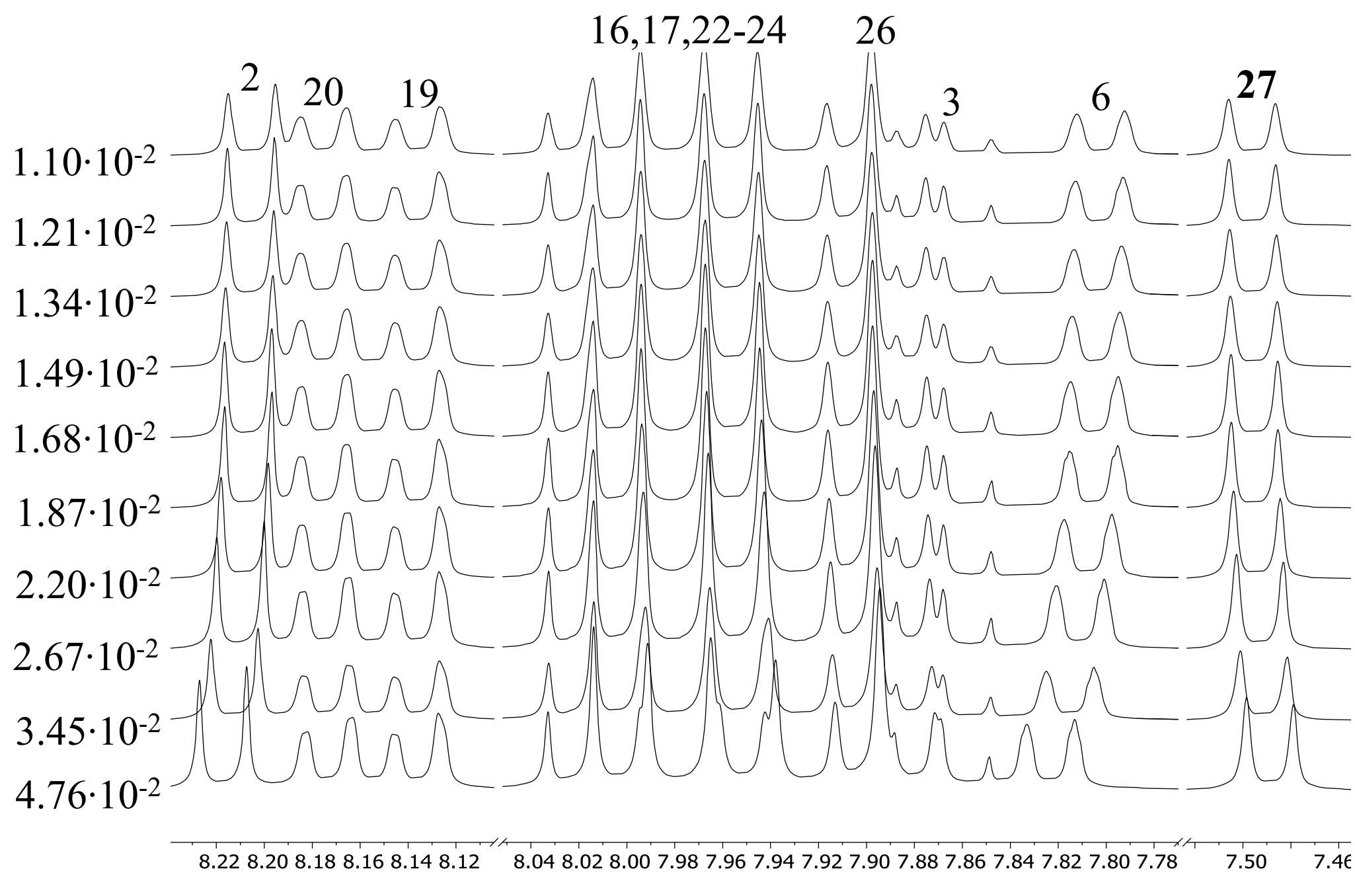

Figure A3-5. ^1H NMR spectrum of **L2** in CD_2Cl_2 upon decreasing concentration for determining the intramolecular association constant at 288 K.

Table A3-8. Chemical shift of H^{27} in ligand **L2** upon ^1H NMR titration at 298 K.

Concentration (10^{-2}·M)	Observed chemical shift (ppm)
4.7599	7.4985
3.4529	7.5010
2.6704	7.5027
2.1970	7.5039
1.8652	7.5049
1.6812	7.5050
1.4855	7.5052
1.3379	7.5055
1.2080	7.5058
1.0974	7.5060

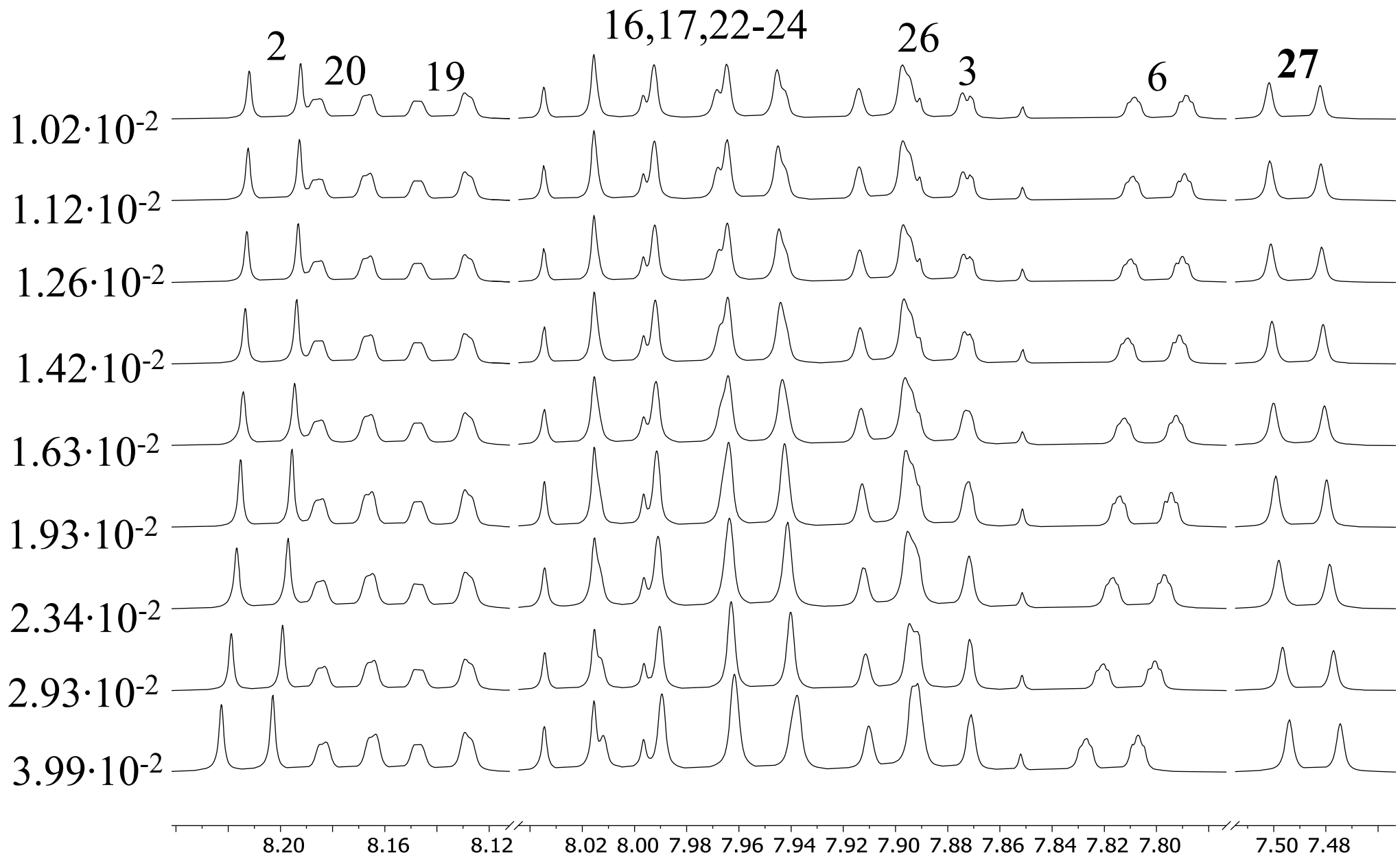

Figure A3-6. ^1H NMR spectrum of **L2** in CD_2Cl_2 upon decreasing concentration for determining the intramolecular association constant at 298 K.

Table A3-9. Chemical shift of H^{27} in ligand **L2** upon ^1H NMR titration at 303 K.

Concentration (10^{-2}·M)	Observed chemical shift (ppm)
3.9852	7.5036
2.9340	7.5058
2.3374	7.5069
1.9269	7.5080
1.6289	7.5087
1.4238	7.5091
1.2557	7.5096
1.1210	7.5099
1.0152	7.5101

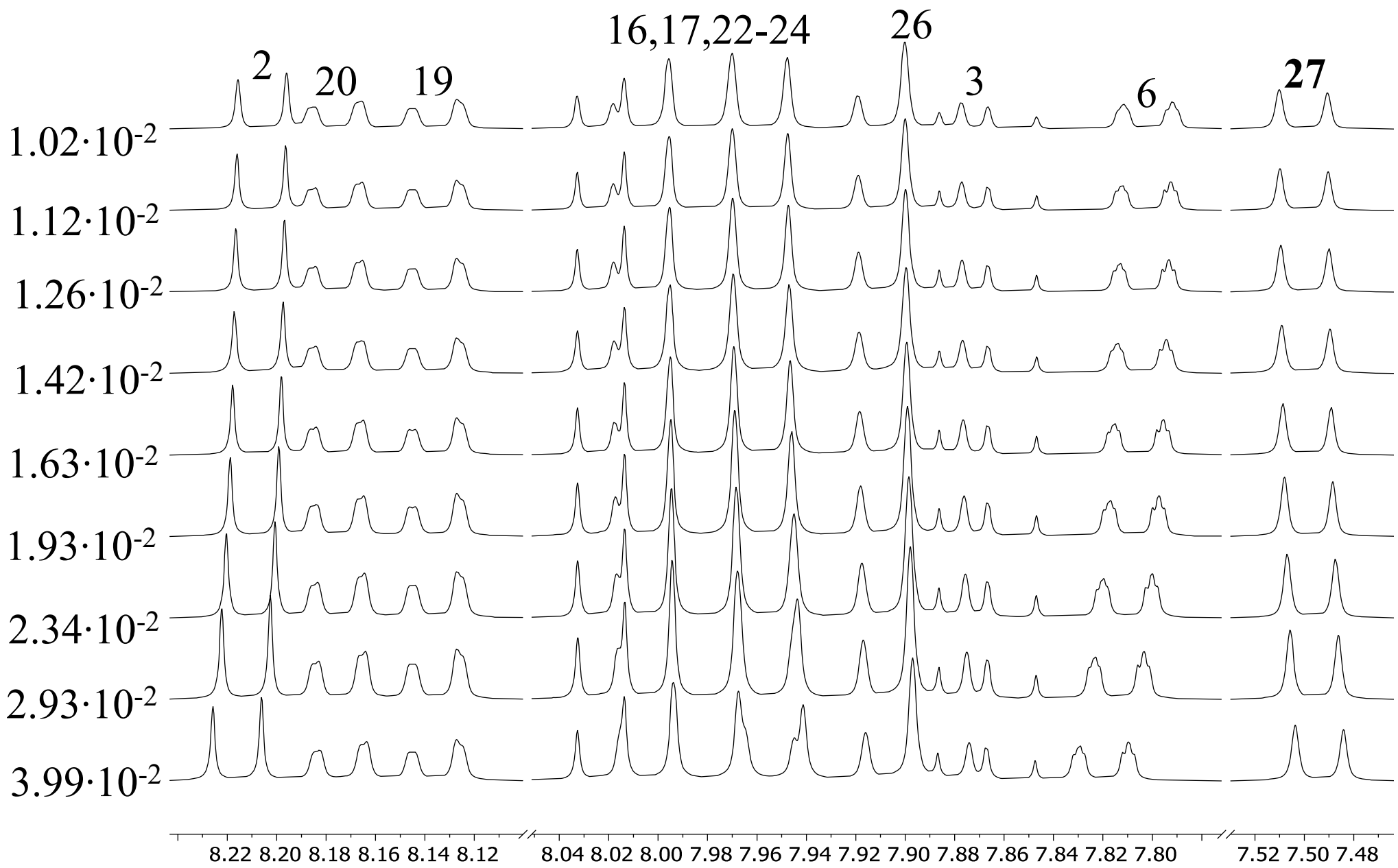

Figure A3-7. ^{1}H NMR spectrum of **L2** in CD_2Cl_2 upon decreasing concentration for determining the intramolecular association constant at 303 K.

Table A3-10. Chemical shift of H^{27} in ligand **L2** upon ^{1}H NMR titration at 308 K.

Concentration (10^{-2}·M)	Observed chemical shift (ppm)
3.9052	7.5070
2.9146	7.5089
2.3114	7.5101
1.9228	7.5109
1.6992	7.5115
1.4675	7.5120
1.2932	7.5124
1.1525	7.5127
1.0282	7.5130

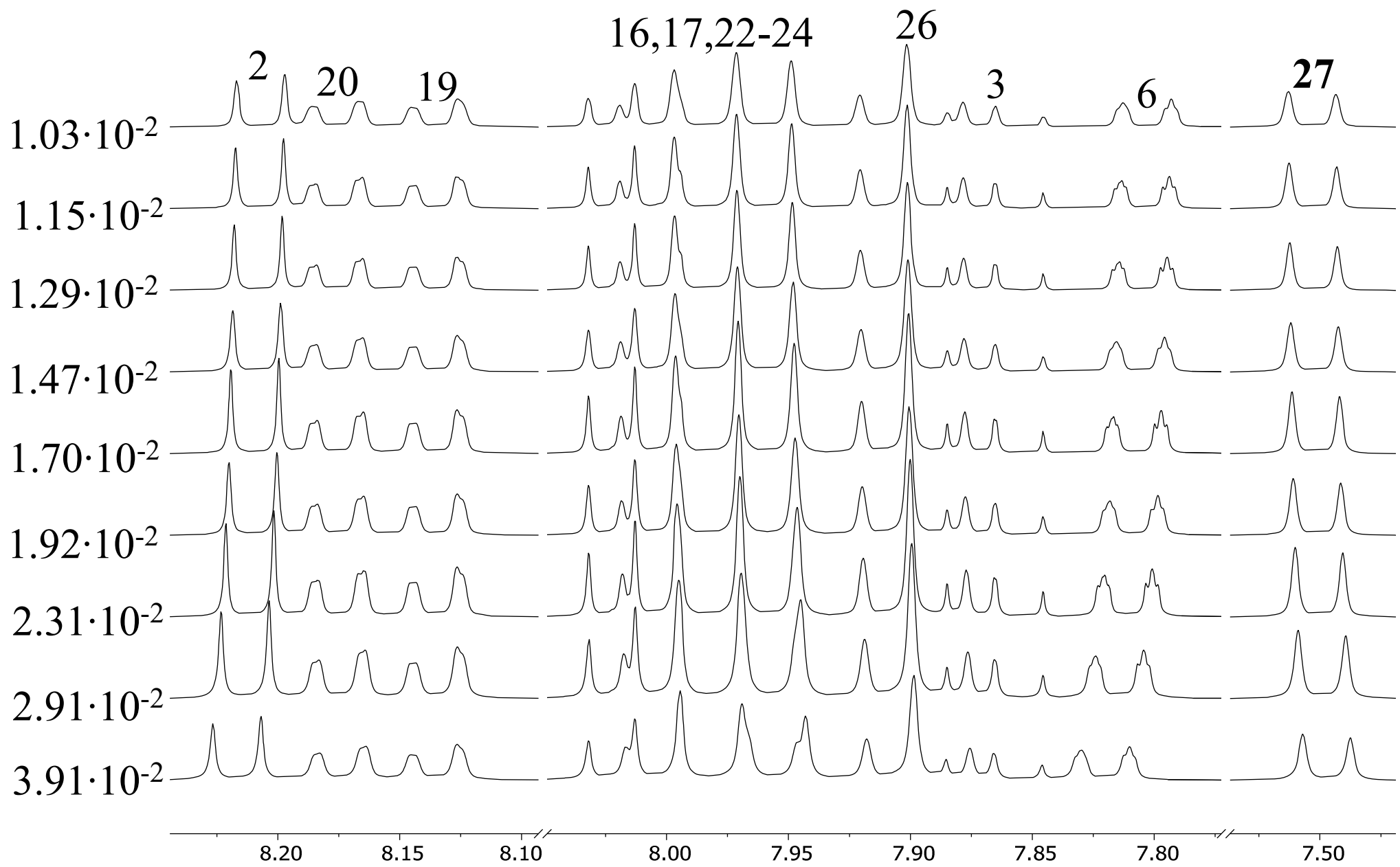

Figure A3-8. ^{1}H NMR spectrum of **L2** in CD_2Cl_2 upon decreasing concentration for determining the intramolecular association constant at 308 K.

Table A3-11. Chemical shift of H^{27} in ligand **L2** upon ^{1}H NMR titration at 313 K.

Concentration (10^{-2}·M)	Observed chemical shift (ppm)
3.9052	7.5101
2.9146	7.5120
2.3114	7.5131
1.9228	7.5139
1.6992	7.5144
1.4675	7.5148
1.2932	7.5152
1.1525	7.5155
1.0282	7.5158

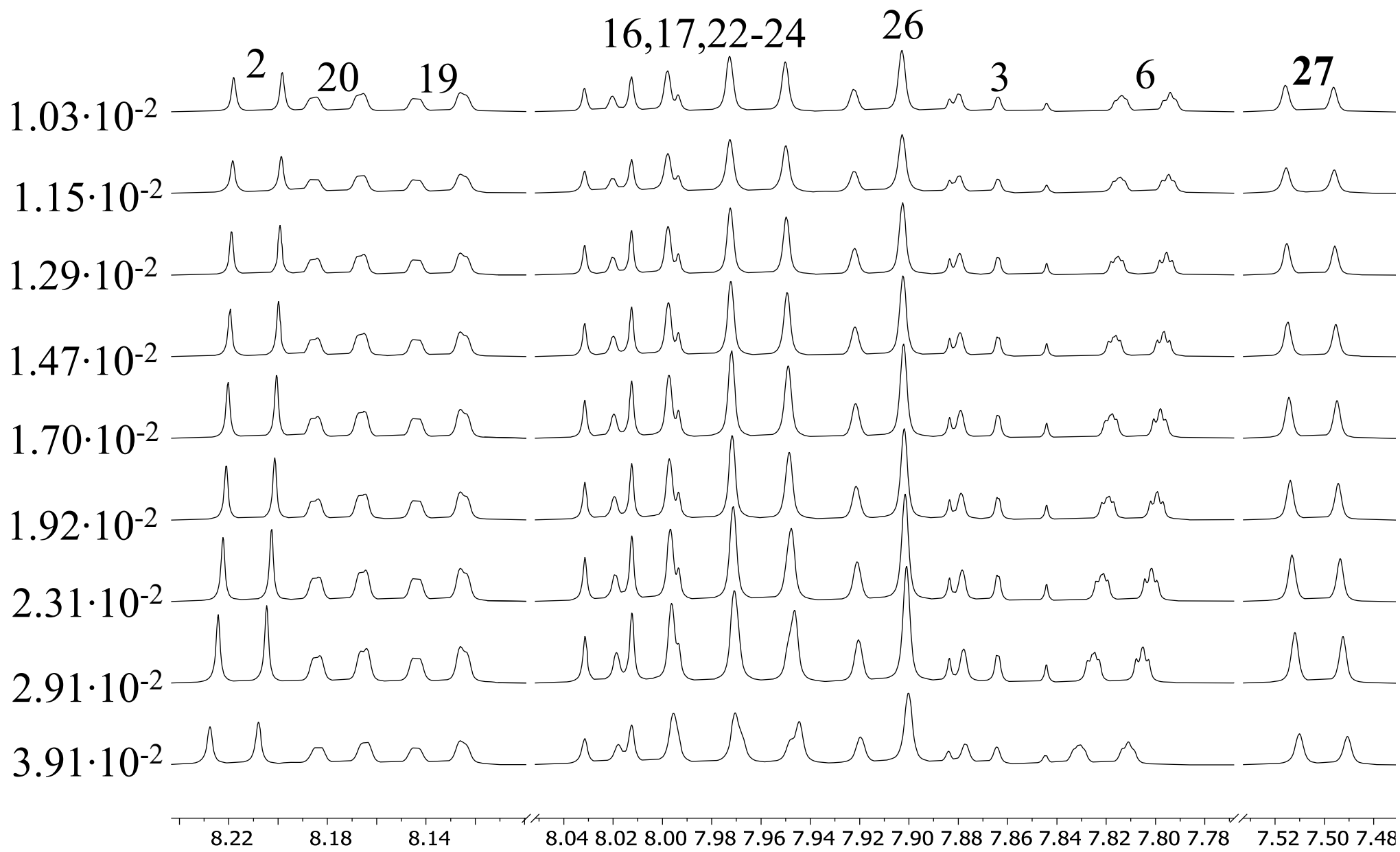

Figure A3-9. ^{1}H NMR spectrum of **L2** in CD_2Cl_2 upon decreasing concentration for determining the intramolecular association constant at 313 K.

Table A3-12. The intramolecular aggregation constant $K_{\text{P-P}}^{\mathbf{L2}\text{Y}}$, $K_{\text{P-T}}^{\mathbf{L2}\text{Y}}$, and the chemical shift $\delta_{\text{P-P}}^{\mathbf{L2}\text{Y}}$, and $\delta_{\text{P-T}}^{\mathbf{L2}\text{Y}}$ obtained from the titrations of [**L2**Y(hfac)3] (H^{27}) at various temperatures.

Temperature (K)	$K_{\text{P-P}}^{\mathbf{L2}\text{Y}}$ /M^{-1}	$\delta_{\text{P-P}}^{\mathbf{L2}\text{Y}}$ /ppm	$K_{\text{P-T}}^{\mathbf{L2}\text{Y}}$ /M^{-1}	$\delta_{\text{P-T}}^{\mathbf{L2}\text{Y}}$ /ppm
293	3.50	7.7178	1.10	8.3492
298	3.02	7.7373	1.01	8.1178
303	0.70	7.4888	0.50	8.0907
308	0.50	7.4049	0.20	8.2024
313	0.32	7.1954	0.07	8.5087

Table A3-13. Chemical shift of H^{27} in ligand [**L2**Y(hfac)3] upon ^{1}H NMR titration at 293 K.

Concentration (10^{-3}·M)	Observed chemical shift (ppm)
4.7293	7.9261
3.5608	7.9266
2.8120	7.9270
2.3190	7.9274
1.9757	7.9274
1.7168	7.9276
1.5212	7.9277
1.3590	7.9278
1.2317	7.9279
10^{-2} (pure monomer)	7.9285

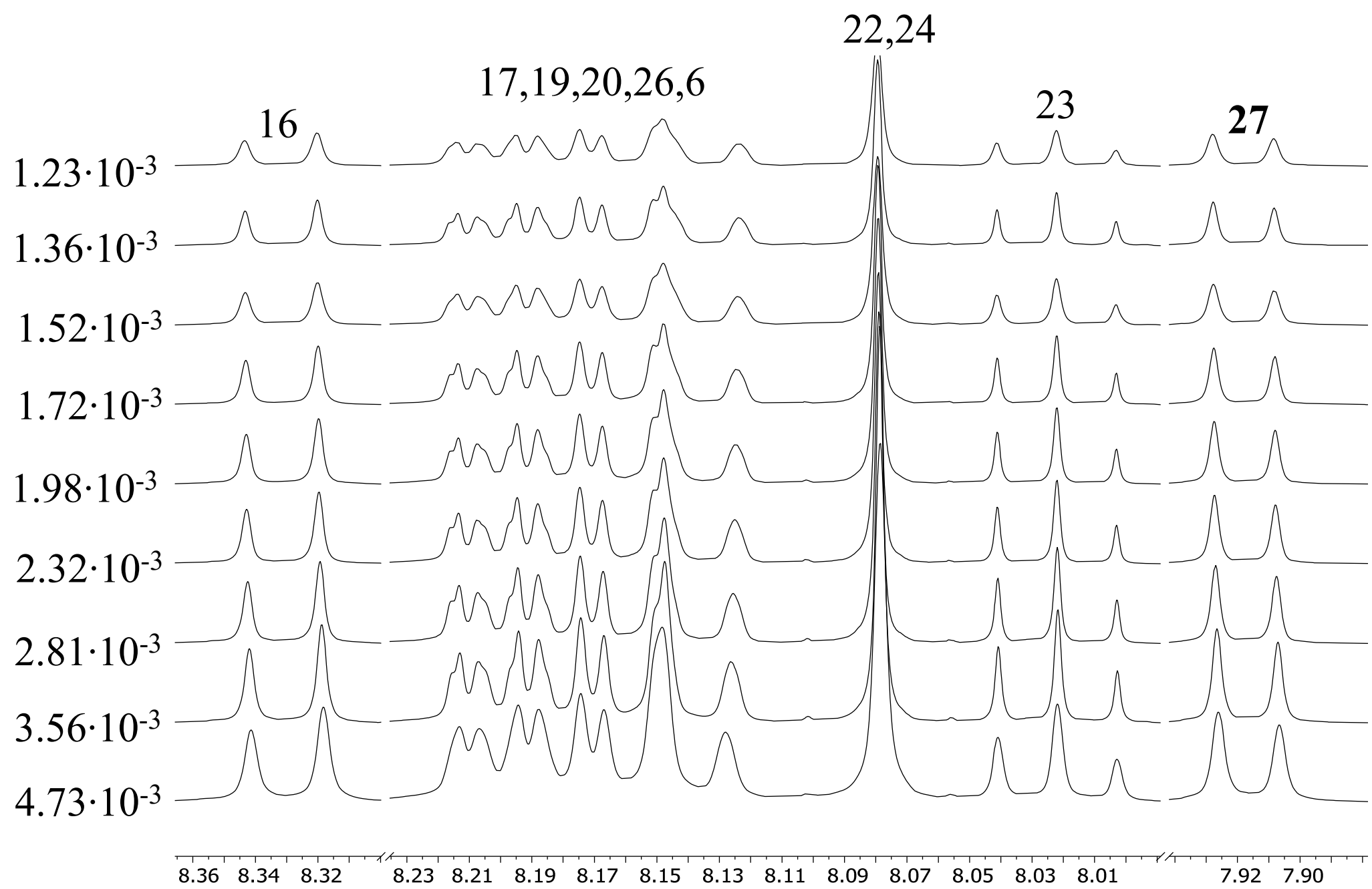

Figure A3-10. ^{1}H NMR spectrum of [**L2**Y(hfac)$_3$] in CD_2Cl_2 upon decreasing concentration for determining the dimerization constants at 293 K.

Table A3-14. Chemical shift of H^{27} in ligand [**L2**Y(hfac)$_3$] upon ^{1}H NMR titration at 298 K.

Concentration (10^{-3}·M)	Observed chemical shift (ppm)
3.7783	7.9263
3.0849	7.9265
2.5873	7.9267
2.2320	7.9268
1.9738	7.9272
1.7588	7.9274
1.5877	7.9277
1.4616	7.9279
1.3342	7.9279
10^{-2} (pure monomer)	7.9288

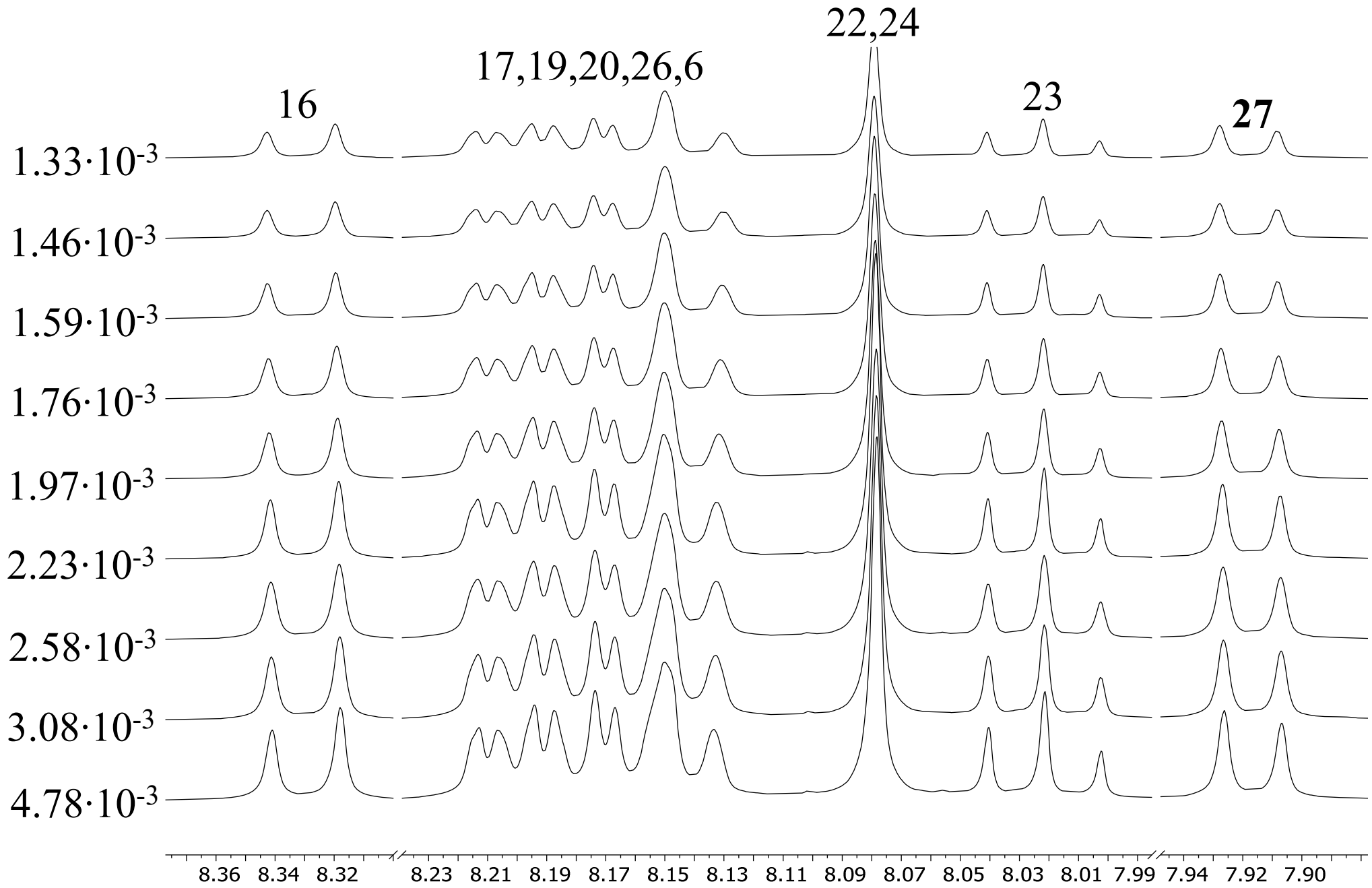

Figure A3-11. ^{1}H NMR spectrum of [**L2**Y(hfac)$_3$] in CD_2Cl_2 upon decreasing concentration for determining the dimerization constants at 298 K.

Table A3-15. Chemical shift of H^{27} in ligand [**L2**Y(hfac)$_3$] upon ^{1}H NMR titration at 303 K.

Concentration (10^{-3}·M)	Observed chemical shift (ppm)
4.7293	7.9273
3.5608	7.9276
2.8120	7.9280
2.3190	7.9283
1.9757	7.9284
1.7168	7.9285
1.5212	7.9286
1.3590	7.9286
1.2317	7.9288
10^{-2} (pure monomer)	7.9293

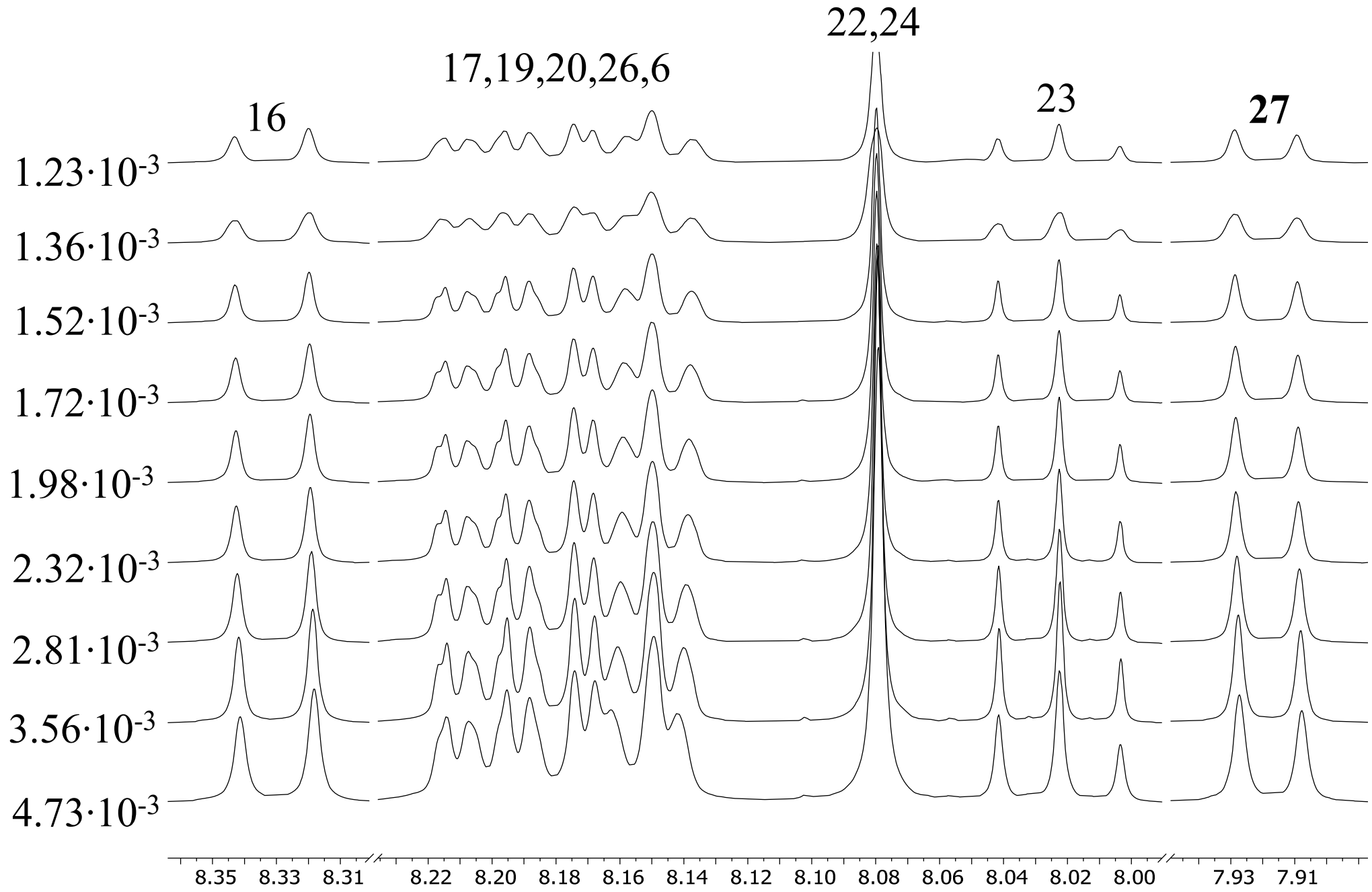

Figure A3-12. ^1H NMR spectrum of [**L2**Y(hfac)$_3$] in CD_2Cl_2 upon decreasing concentration for determining the dimerization constants at 303 K.

Table A3-16. Chemical shift of H^{27} in ligand [**L2**Y(hfac)$_3$] upon ^1H NMR titration at 308 K.

Concentration (10^{-3}·M)	Observed chemical shift (ppm)
4.7293	7.9279
3.5608	7.9281
2.8120	7.9285
2.3190	7.9288
1.9757	7.9289
1.7168	7.9290
1.5212	7.9291
1.3590	7.9290
1.2317	7.9291
10^{-2} (pure monomer)	7.9297

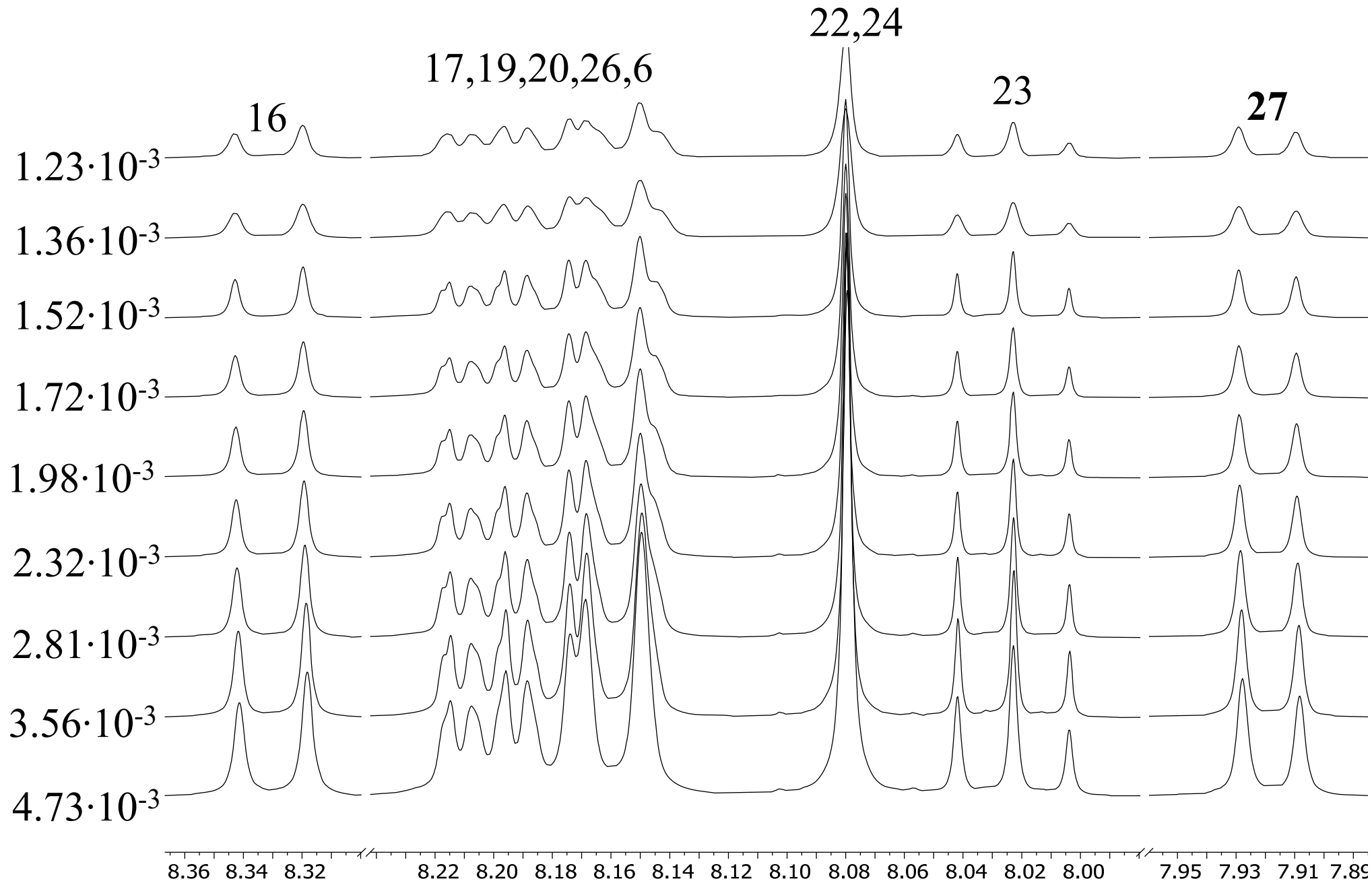

Figure A3-13. ^{1}H NMR spectrum of [**L2**Y(hfac)$_3$] in CD_2Cl_2 upon decreasing concentration for determining the dimerization constants at 308 K.

Table A3-17. Chemical shift of H^{27} in ligand [**L2**Y(hfac)$_3$] upon ^{1}H NMR titration at 313 K.

Concentration (10^{-3}·M)	Observed chemical shift (ppm)
4.7293	7.9273
3.5608	7.9276
2.8120	7.9280
2.3190	7.9283
1.9757	7.9284
1.7168	7.9285
1.5212	7.9286
10^{-2} (pure monomer)	7.9293

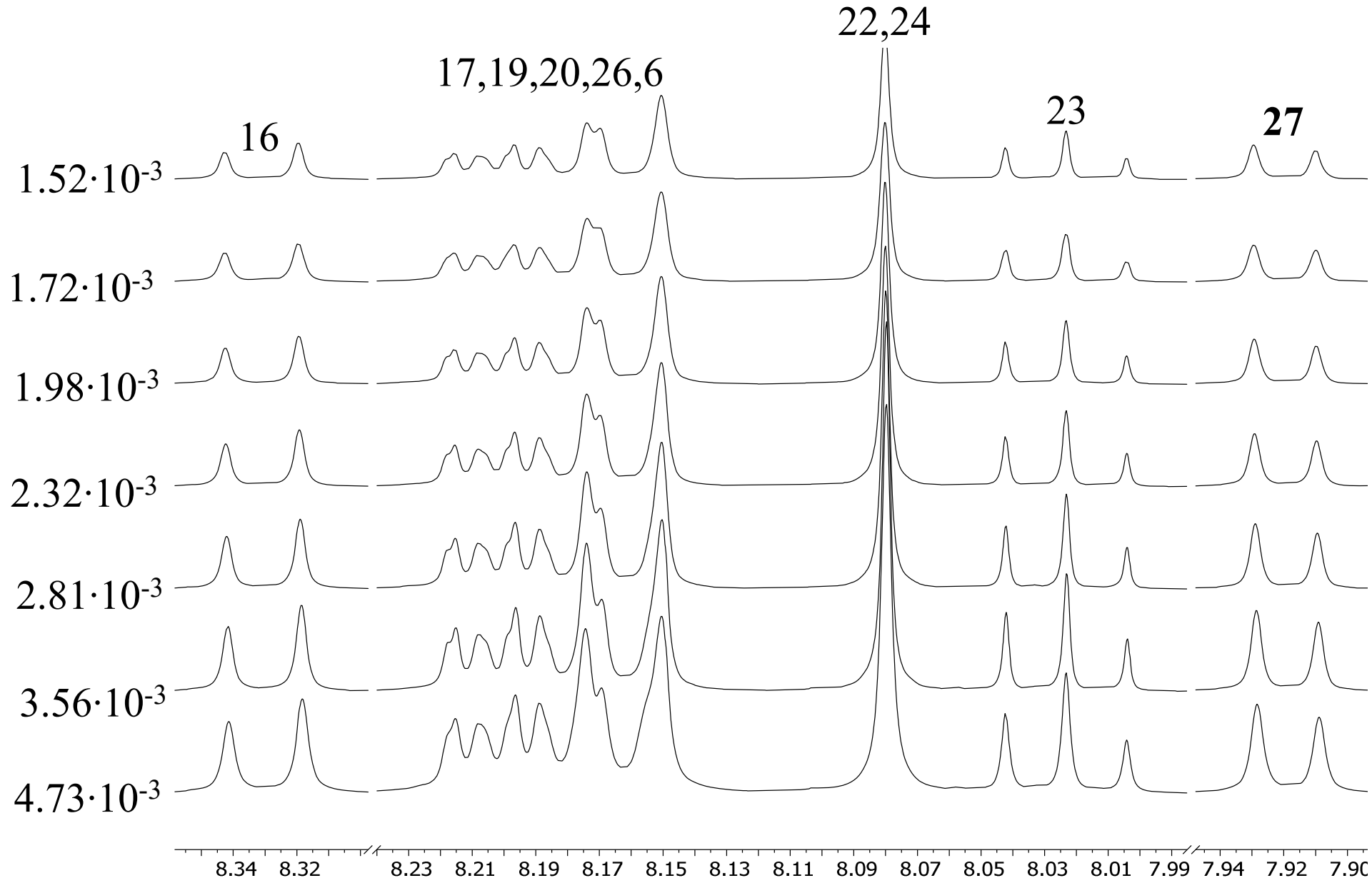

Figure A3-14. ^{1}H NMR spectrum of [**L2**Y(hfac)$_3$] in CD_2Cl_2 upon decreasing concentration for determining the dimerization constants at 313 K.

Appendix 4: Photophysical parameters

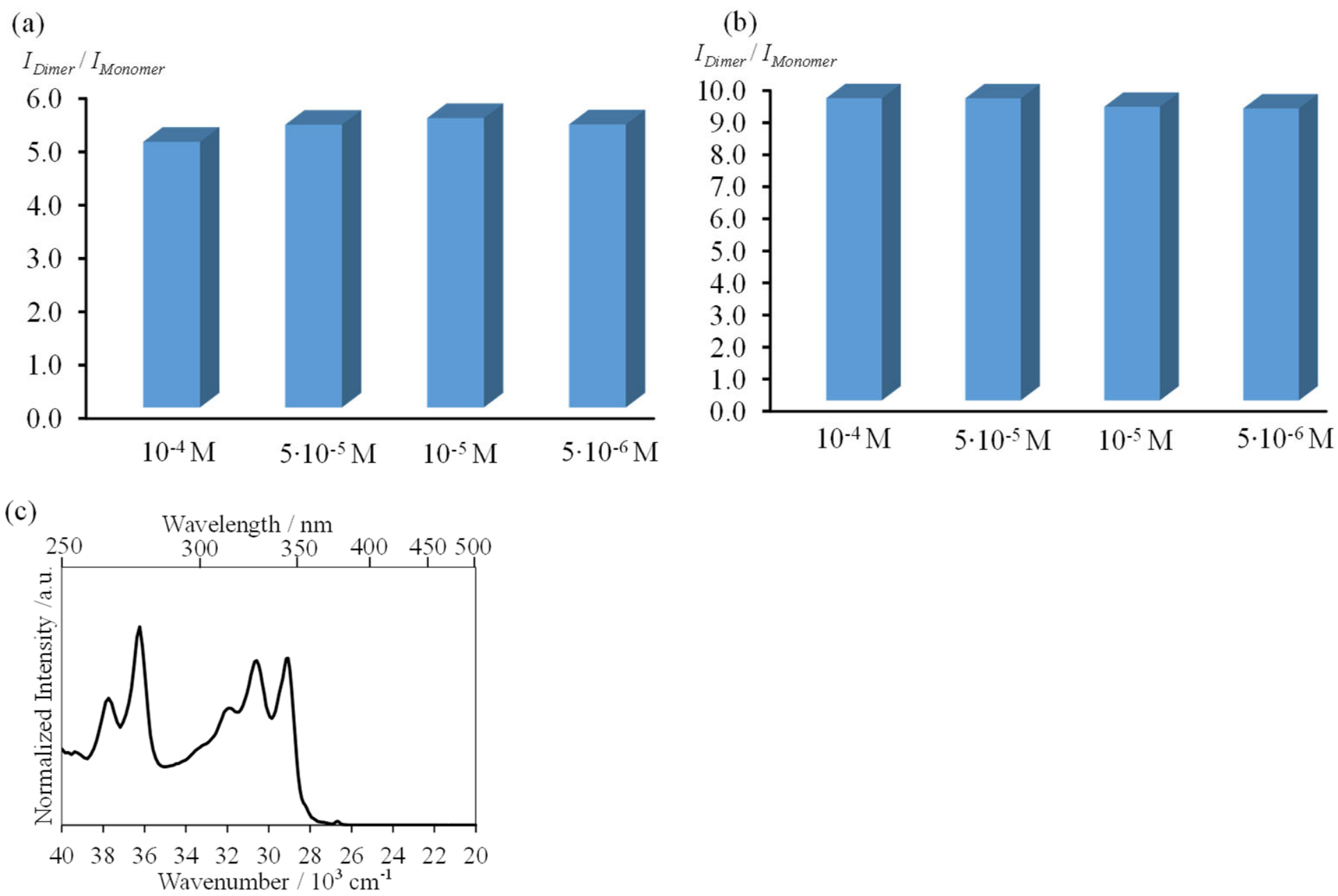

Figure A4-1. The excimer/monomer intensity ratio of (a) [**L2**Y(hfac)$_3$], and (b) **L2** at various concentrations; (c) excitation spectrum of **L2** monitored 478 nm.
